# JCS/JHRS 2024 Guideline Focused Update on Management of Cardiac Arrhythmias

**DOI:** 10.1002/joa3.70033

**Published:** 2025-06-16

**Authors:** Yu‐ki Iwasaki, Takashi Noda, Masaharu Akao, Tadashi Fujino, Teruyuki Hirano, Koichi Inoue, Kengo Kusano, Toshiyuki Nagai, Kazuhiro Satomi, Tetsuji Shinohara, Kyoko Soejima, Yohei Sotomi, Shinya Suzuki, Teiichi Yamane, Tsukasa Kamakura, Hiroyuki Kato, Arimi Katsume, Yusuke Kondo, Kenji Kuroki, Hisaki Makimoto, Hiroshige Murata, Takafumi Oka, Nobuaki Tanaka, Nobuhiko Ueda, Hiro Yamasaki, Seigo Yamashita, Ryobun Yasuoka, Kenji Yodogawa, Kazutaka Aonuma, Takanori Ikeda, Toru Minamino, Hideo Mitamura, Akihiko Nogami, Ken Okumura, Hiroshi Tada, Takashi Kurita, Wataru Shimizu, Japanese Circulation Society and Japanese Heart Rhythm Society Joint Working Group

**Abbreviations**
ACC: American College of Cardiology
ACT: activated clotting time
ADL: activities of daily living
AF: atrial fibrillation
AHA: American Heart Association
BMI: body mass index
BMS: bare metal stent
CCI: Charlson comorbidity index
CCr: creatinine clearance
CFAE: complex fractionated atrial electrogram
CKD: chronic kidney disease
CLBBB: complete left bundle branch block
CLS: closed loop stimulation
CMR: cardiac magnetic resonance
CO: cardiac output
COPD: chronic obstructive pulmonary disease
CRT: cardiac resynchronization therapy
CRT‐D: cardiac resynchronization therapy defibrillator
CRT‐P: cardiac resynchronization therapy pacemaker
CSP: conduction system pacing
CT: computed tomography
DBP: diastolic blood pressure
DDD/DDDR: dual chamber pacing, dual chamber sensing, and pacemaker activation or inhibition on a sensed event / rate‐modulated pacing
DOAC: direct oral anticoagulant
ECG: electrocardiogram
eGFR: estimated glomerular filtration rate
EPS: electrophysiological study
EV‐ICD: extra vascular‐implantable cardioverter defibrillator
FAAM: fractionated signal areas in the atrial muscle
FDA: US Food and Drug Administration
FIRM: focal impulse and rotor modulation
FNNC: filamin C
FXa: factor Xa
HBP: His bundle pacing
HFpEF: heart failure with preserved ejection fraction
HFrEF: heart failure with reduced ejection fraction
HRS: Heart Rhythm Society
ICD: implantable cardioverter defibrillator
ILAM: isochronal late activation map
INR: international normalized ratio
LBBA: left bundle branch area
LBBAP: left bundle branch area pacing
LBBB: left bundle branch block
LGE: late gadolinium enhancement
LMNA: lamin A/C
LVA: low‐voltage areas
LVAD: left ventricular assist device
LVEF: left ventricular ejection fraction
MMSE: mini‐mental state examination
MRI: magnetic resonance imaging
MRSA: methicillin‐resistant Staphylococcus aureus
NCDR: National Cardiovascular Data Registry
NSAIDs: non‐steroidal anti‐inflammatory drugs
NSVT: non‐sustained ventricular tachycardia
NYHA: New York Heart Association
PCI: percutaneous coronary intervention
PCWP: pulmonary capillary wedge pressure
PFA: pulsed field ablation
PLN: phospholamban
PVC: premature ventricular contraction
PVI: pulmonary vein isolation
PVS: pulmonary vein stenosis
QOL: quality of life
RBM20: RNA binding motif protein 20
RCT: randomized controlled trial
RVP: right ventricular pacing
SBP: systolic blood pressure
SCD: sudden cardiac death
S‐ICD: subcutaneous implantable cardioverter defibrillator
SPRM: Seattle proportional risk model
SUV: standard uptake value
V6RWPT: R wave peak time in lead V6
VDD: atrial‐synchronized ventricular pacing (ventricle pacing, dual chamber sensing, and pacemaker activation or inhibition on a sensed event)
VOM: vein of Marshall
VOM‐EI: ethanol injection into the vein of Marshall
VF: ventricular fibrillation
VT: ventricular tachycardia
VVI/VVIR: ventricular demand pacing (ventricle pacing, ventricle sensing, and pacemaker inhibition on a sensed event) / rate‐modulated pacing
6MWD: 6‐minute walking distance
18F‐FDG‐PET: 18F‐fluorodeoxyglucose‐positron emission tomography

## TABLE OF CONTENTS

**Table:** 

**Foreword**	2
**I. Implantable Cardiac Electrical Devices**	3
1. Indications for Primary Prevention of Implantable Cardioverter Defibrillators (ICDs)	3
2. ICD indications for Cardiac Sarcoidosis	6
3. Leadless Pacemaker	8
PQ1. How to Choose Between a Leadless Pacemaker and a Transvenous Pacemaker	11
4. Pacemaker Therapy for Reflex Syncope	11
5. Future Advances in Implantable Cardiac Electrical Devices	11
6. Conduction System Pacing (CSP)	12
7. CRT for Mid‐Range QRS	17
**II. Catheter Ablation**	19
1. Catheter Ablation Procedures for Atrial Fibrillation in Addition to Pulmonary Vein Isolation	19
2. Expanded Indication for Atrial Fibrillation Catheter Ablation	21
CQ1. Should Atrial Fibrillation Catheter Ablation Be Performed in Older Patients (>80 Years Old)?	26
3. New Ablation Therapy for Atrial Fibrillation: Pulsed	27
4. Advances in Ventricular Premature Contraction (PVC)/Ventricular Tachycardia (VT) Ablation	29
CQ2. What Is the Optimal Treatment for Asymptomatic Idiopathic PVC Without Evidence of Left Ventricular Dysfunction?	31
**III. Atrial Fibrillation Pharmacotherapy and Comprehensive Management**	32
1. Japanese Original Stroke Risk Assessment Tool: HELT‐E_2_S_2_ Score	32
PQ2. Which Patients Are Excluded From Anticoagulation Therapy?	35
2. Anticoagulation for High‐Risk Older Patients	36
3. Specific Neutralizers for Factor Xa Inhibitors	41
4. Digitalis and Atrial Fibrillation	43
5. Atrial Fibrillation and Lifestyle Management / Comprehensive Management	44
**References**	47
**Appendix 1 Details of Members**	63
**Appendix 2 Disclosure of Potential Conflicts of Interest**	64

## FOREWORD

### 1 On the Focus Update

The Guidelines for the Pharmacological Treatment of Arrhythmias were first published in 2004, followed by the 2009 revision,^1^ the 2013 revision of the Guidelines for the Pharmacological Treatment of Atrial Fibrillation (Drugs),^2^ and a complete revision in 2020 as the Revision of the Guidelines for the Pharmacological Treatment of Arrhythmias.^3^

During the several years since the last revision, drugs related to arrhythmia therapy have become available for clinical use, and evidence has been reported to revise the efficacy of conventional drugs. In particular, anticoagulation therapy for the prevention of cerebral infarction and systemic embolism has become widely used in the treatment of atrial fibrillation (AF), and a variety of evidence including neutralizing agents has been accumulated from Japan and overseas.

In Japan, the aging population is not only prescribed drugs for a single arrhythmic disease, but also for a variety of clinical backgrounds, such as frailty and cognitive function, which must be taken into consideration. The importance of comprehensive management, which includes not only drug therapy but also the identification and intervention of various modifiable risk factors, is now recognized worldwide.

Since the publication of the Guidelines for the Nonpharmacologic Treatment of Arrhythmias in 2000, guidelines for catheter ablation, pacemaker and implantable cardioverter‐defibrillator (ICD) therapy, and arrhythmia surgery have been revised in 2006 and 2011.^4^ In addition, the atrial fibrillation catheter ablation technique has become common practice due to remarkable progress in medical engineering technology and the establishment of treatment techniques and surgical procedures, diversifying the nonpharmacological treatment of arrhythmia. The 2019 revision^5^ was further revised as the 2021 JCS/JHRS Guideline Focus Update for Nonpharmacologic Treatment of Arrhythmias.^6^

As noted above, nonpharmacological treatment of arrhythmias has developed at an astonishing pace and is now being utilized for many patients. In Japan, where sudden cardiac deaths are estimated to be as many as 80,000 per year,^7^ and a heart failure pandemic is expected to hit in 2025, the role of nonpharmacological therapies such as catheter ablation and ICDs is extremely important, and the demand for these therapies is expected to increase in the future.

Arrhythmia treatment has traditionally been divided into pharmacological and nonpharmacological, with respective guidelines being developed. However, they are not mutually exclusive treatments and in practice, hybrid therapies are often used, with many clinical trials now being conducted to establish evidence for this. In order to emphasize consistency and uniformity in the treatment of arrhythmias and to enhance convenience for practicing physicians involved in arrhythmia treatment, the 2024 JCS/JHRS Guideline Focus Update for Arrhythmia Treatment (hereinafter referred to as the Focus Update) was developed to unify “arrhythmia pharmacotherapy” and “arrhythmia nonpharmacological treatment”.

### 2 Recommended Class and Level of Evidence

In this Focus Update, the recommended classes and levels of evidence are categorized as shown in **Table** **1** and **Table** **2**. Considering consistency with arrhythmia guidelines in the USA and Europe, the wording of the recommended classes is consistent. Arrhythmia treatment includes many treatments that have been used for a long time, and there is insufficient evidence from randomized controlled trials and other sources, making it difficult to conduct a systematic review using a uniform literature search formula. For this reason, we omitted the Minds recommended grades and Minds evidence level classification based on the Minds Clinical Practice Guideline Development Guide.

**TABLE 1:** Recommended Class Classification.

Class I	There is evidence or widespread agreement that the procedure/treatment is effective and useful
Class IIa	Likely to be effective/useful based on evidence/opinions
Class IIb	Evidence/opinion indicates that efficacy and usefulness are not so well established
Class III (No benefit)	There is evidence that the procedure/treatment is not effective or useful. Or there is a broad consensus of opinion
Class III (Harm)	There is evidence or widespread agreement that the procedure/treatment is harmful

**TABLE 2:** Levels of Evidence.

Level A	Demonstrated in multiple randomized interventional clinical trials or meta‐analysis
Level B	Demonstrated in a single randomized intervention clinical trial or a large non‐randomized intervention clinical trial
Level C	Consensus among experts and/or small clinical trials (including backward‐looking studies and registries)

### 3 Clinical Questions

The Japanese Circulation Society guidelines have introduced a format in which clinical questions (CQs) are set, a systematic review is conducted using the GRADE system, and recommendations are clearly indicated. Because this is a focused update, we did not establish a systematic review group independent of the guideline writing committee members, and instead, we developed 2 CQs that occur in daily practice. In addition, 2 themes that may be helpful in deciding on a treatment plan are also included as Practical Questions (PQs) to answer clinical questions.

### 4 Providing Information to the Public and Patients

Currently, the readers of the Japanese Circulation Society guidelines include not only specialists of the disease in question who are engaged in actual medical treatment, but also the general public and patients, in addition to nonspecialists and primary care physicians. This diversification of guideline users is due to the growing importance of the process of sharing information about diseases and treatment between patients and their families and healthcare professionals, and forming a consensus through thorough consultation. Moreover, in promoting shared decision‐making, providing information to citizens and patients is crucial, and this focus update guideline includes six topics related to arrhythmia treatment.

### 5 Publication of the Guidelines and Conflicts of Interest

This Focus Update was jointly prepared by the Japanese Circulation Society and the Japanese Heart Rhythm Society, with the participation of the Japanese Association of Cardiovascular Intervention and Therapeutics, the Japanese Heart Failure Society, and the Japanese Stroke Society. Six external reviewers were asked to review the manuscript, and revisions were made as necessary based on the opinions obtained.

Finally, the role of this 2024 Guideline Focused Update on Management of Cardiac Arrhythmias, is to provide information that will enable safe and effective implementation of the latest treatments in daily practice. However, in actual clinical practice, it is difficult to provide uniform treatment because of the variety of patients, their clinical backgrounds, and responses to treatment. The final decision regarding specific patient care and management should be made by the physician and medical staff in charge of the patient, who set individual goals with the patient and family, and share information and intentions as appropriate. We hope that this Focus Update will help in this regard.

## 1 IMPLANTABLE CARDIAC ELECTRICAL DEVICES

## 2 INDICATIONS FOR PRIMARY PREVENTION OF IMPLANTABLE CARDIOVERTER DEFIBRILLATORS (ICDS)

Several randomized controlled trials (RCTs) have investigated the role of ICDs for primary prevention in patients with reduced left ventricular ejection fraction (LVEF), and have shown efficacy in preventing sudden cardiac death (SCD) in heart failure patients with LVEF ≤35%.^8^, ^9^ On the other hand, the DANISH trial, a prospective comparative study of ICDs in 1,116 patients with nonischemic cardiomyopathy, showed no clear mortality benefit of ICDs for primary prevention in patients with nonischemic cardiomyopathy.^10^ A meta‐analysis of 6 trials for nonischemic cardiomyopathy, including DANISH,^11^ showed that ICDs significantly reduced relative mortality; however; it was unclear whether the ICD was more useful in selected patients. It is necessary to identify the patient population in which ICDs are most useful.

In the subanalysis of the Nippon Storm study, Sasaki et al. reported that the incidence of appropriate ICD therapy in nonischemic cardiomyopathy patients for primary prevention was 21%, during a mean follow‐up of 775 days.^12^ The HINODE study^13^ showed that the mortality and appropriate ICD therapy rates were similar to those in MADIT‐RIT for Japanese heart failure patients. In that study, 171 propensity‐matched patients for primary prevention from among 354 enrolled patients were compared to 985 patients in the MADIT‐RITstudy,^14^ which revealed no significant differences in annual survival rates (96.3% in the HINODE group vs. 96.9% in the MADIT‐RIT group, P=0.29) or annual appropriate ICD therapy‐free rates (94.7% vs. 96.8%, P=0.61) between the 2 groups. The incidence of fatal arrhythmias in patients with heart failure in Japan in recent years is comparable to that in Europe and the USA, but higher than previously thought.

### 2.1 Elderly Patients (Table 3)

**TABLE 3:** Recommendations and Levels of Evidence for Primary Prevention Indications for ICDs.

	COR	LOE
Assessment of comorbidities, including frailty and dementia, should be considered to identify patients at high risk of arrhythmic death and low risk of non‐arrhythmic death when considering the ICD indications in elderly patients	IIa	C
Use of the MADIT‐ICD Benefit Score*^1^ or the SPRM*^2^ scoring should be considered to assess the risk of arrhythmic and non‐arrhythmic death in HF patients when determining the ICD indication	IIa	B
ICD implantation should be considered in non‐ischemic cardiomyopathy patients with an LVEF <50%, LGE on CMR, and either pathogenic mutation in LMNA, PLN, FLNC, and RBM20 genes	IIa	B

*^1^ https://redcap.urmc.rochester.edu/redcap/surveys/index.php?s=3H888TJ8N7 (accessed November 2023).

*^2^ https://depts.washington.edu/sprm/about.php (accessed November 2023).

CMR, cardiac magnetic resonsnce; COR, Class of Recommendation; EPS, electrophysiological study; FLNC, filamin C; HF, heart failure; ICD, implantable cardioverter defibrillator; LGE, late gadolinium enhancement; LMNA, lamin A/C; LOE, Level of Evidence; LVEF, left ventricular ejection fraction; PLN, phospholamban; RBM20, RNA binding motif protein 20; SPRM, Seattle Proportional Risk Model; VT, ventricular tachycardia.

Use of an ICD as primary prevention in elderly patients is effective in preventing SCD from fatal arrhythmias; however; it is essential to carefully consider the indication for ICD because of the higher risk of non‐arrhythmic death from concomitant comorbidities compared with younger patients. Zakine et al.^15^ compared the clinical outcomes of 150 patients with ICDs aged >80 years and 150 patients with ICDs aged <80 years from among 8,333 screened patients from 15 centers. During a mean follow‐up of 3.0 years, there were no significant differences in appropriate ICD therapy (19.4% vs. 21.6%, P=0.48) or complications related to the ICD (21.2% vs. 14.0%, P=0.10), but the all‐cause mortality rate was significantly higher in the elderly patients (36.3% vs. 12.9%, P=0.005). In the MADIT‐ICD benefit score constructed from 4 MADIT studies, age (≥75 years) was associated with increased risk of non‐arrhythmic death.^16^.

The EU‐CERT‐ICD^17^ is a prospective cohort study of 2,247 patients with ischemic and non‐ischemic cardiomyopathy (1,516 in the ICD implantation group and 731 in the non‐ICD implantation group, New York Heart Association [NYHA] functional class II/III: LVEF ≤35%; NYHA functional class I: LVEF ≤30%) enrolled from 44 centers in 15 European countries. Multivariable models and propensity score matching revealed that the overall mortality rate was significantly lower in the ICD implantation group than in the control group (5.6%/year vs. 9.2%/year, hazard ratio [HR] 0.73, P=0.014), but there was no significant mortality reduction by ICD in patients aged ≥75 years (HR 1.06, P=0.821). A subanalysis of DANISH, a randomized controlled trial of ICDs in 1,116 patients with nonischemic cardiomyopathy, also found a mortality reduction with ICDs in patients aged ≤70 years (HR 0.70, P=0.03), but not in patients >70 years (HR 1.05, P=0.84). The non‐sudden death rate was 2.7 per 100 person‐years in patients ≤70 years, and 5.4 per 100 person‐years in patients >70 years, indicating a difference in modes of death.^18^.

Thus, the risk of non‐arrhythmic death is higher in the older patient than in the young, and an ICD has a limited effect on mortality reduction. Therefore, when determining the indication for primary prevention ICD in older patients, it is important to select patients in whom ICDs will be highly effective in preventing arrhythmic death and in whom the risk of non‐arrhythmic death is low.

It is important to assess frailty, dementia, and comorbidities when considering the risk of non‐arrhythmic death.^19^ In the National Cardiovascular Data Registry (NCDR), a cardiovascular disease database in the USA, 83,792 patients with primary prevention ICDs enrolled from 2006 to 2009 were reported to have frailty in 10% and dementia in 1%. The 1‐year mortality rate was 22% in patients with frailty and 27% in those with dementia, compared with 12% overall.^20^ A meta‐analysis of frailty and ICDs found a correlation between frailty and death, but the report pointed out that the definition of frailty was not consistent across studies and included the cumulative deficit model, low physical component summary score, low body weight, and 6‐minute walking distance (6MWD).^21^.

In a report on 121 elderly patients after ICD implantation,^22^ higher Charlson Comorbidity Index (CCI),^23^ a score used to assess comorbidities for the prediction of death, significantly decreased survival rates, and the 5‐year survival rates for patients with a CCI of 0 to 1, 2–3, and ≥4 were 78%, 57%, and 29%, respectively. When considering the indication for an ICD in the older patient, it is necessary to consider frailty, cognitive function, and comorbidities for each individual case.

### 2.2 Risk Factors for Fatal Arrhythmias and ICD Indications (Table 3)

Personalized assessment of the risk of SCD from fatal arrhythmias and the risk of non‐arrhythmic death should be considered when determining the indication for primary‐prevention ICD. Recently, a score that predicts fatal arrhythmias and non‐arrhythmic deaths was reported. The MADIT‐ICD benefit score is based on 8 predictors of life‐threatening arrhythmias (LVEF ≤25%, atrial arrhythmia, heart rate >75 beats/min, systolic blood pressure <140 mmHg, myocardial infarction, age <75 years, male sex, and prior non‐sustained ventricular tachycardia [NSVT]), and 7 predictors of non‐arrhythmic death (ICD or implantable cardioverter‐defibrillator with biventricular pacing [CRT‐D], NYHA class >II, diabetes mellitus, body mass index [BMI] <23 kg/m^2^, atrial arrhythmia, LVEF <25%, age >75 years) (https://is.gd/madit).^16^ In the same score, the risk of fatal arrhythmia is approximately 3‐fold higher than the risk of non‐arrhythmic death in the group with the highest score (76–100) (20% vs. 7%, P<0.001). Dauw et al. also examined the usefulness of the MADIT‐ICD benefit score in 475 cardiac resynchronization therapy (CRT) patients and found that the rates of fatal arrhythmia were 1.8% in the lowest benefit score group, 4.1% in the intermediate benefit score group, and 14.4% in the highest benefit score groups while arrhythmic deaths were 19.4%, 14.6%, and 3.3%, respectively.^24^ Thus, the MADIT‐ICD benefit score may be useful for identifying the need for defibrillation function in CRT patients. However, Fukuoka et al. pointed out that this scoring system has the limitations that few Asian patients were included in the RCTs, and they are considered to be at low risk of SCD,^25^, ^26^, ^27^, ^28^ and that patients with multiple comorbidities and relatively preserved cardiac function were not included.^29^.

An ICD indication using the Seattle Proportional Risk Model (SPRM, http://depts.washington.edu/sprm/about.php) to predict risk of arrhythmic death and all‐cause death has also been proposed. The SPRM is a predictive model of sudden and non‐sudden death based on the clinical backgrounds of 9,885 heart failure patients without ICD implantation, and it uses NYHA functional class, diabetes mellitus, digoxin use, age, BMI, LVEF, systolic blood pressure, serum sodium level, and serum creatinine level as assessment factors.^30^ Bilchick et al.^31^ examined the validity of the SPRM using the NCDR registry, which is the American College of Cardiology's suite of cardiovascular data registries. They performed overall survival analysis in quintiles of 98,846 patients with heart failure with reduced ejection fraction (HFrEF, LVEF ≤35%; 87,914 with ICD implantation and 10,932 without ICD implantation) using the SPRM. Fukuoka et al. validated the SPRM in a Japanese registry of heart failure patients, and reported that a 30% reduction in mortality could be expected with an ICD in 667 Japanese patients with LVEF ≤35% and at high risk of SCD as assessed by the SPRM.^32^.

The usefulness of scoring systems regarding ICD indication that includes both the risk of fatal arrhythmias and the risk of non‐arrhythmic death has been reported, however; these risk stratification scores need to be validated in Japanese cohorts. In determining the indication for ICD, the benefit of an ICD should be assessed comprehensively, taking into account not only scores, but also the benefits of the ICD, including the patient's life expectancy, and comorbidities. It is also important to provide sufficient information to the patient to enable comprehensive patient‐centered decision‐making.

### 2.3 Ischemic Cardiomyopathy

It was believed that patients with ischemic cardiomyopathy in Japan had a low risk of SCD after myocardial infarction,^33^, ^34^ and the use of ICD in patients with an ICD indication was reportedly low in real‐world clinical practice.^35^.

It is well‐known that primary‐prevention patients have a lower rate of ICD therapy than secondary‐prevention patients.^36^ However, in a subanalysis of the Nippon Storm study of 493 patients with ischemic cardiomyopathy and an ICD, in which propensity score matching was used for selecting 133 patients for primary prevention and 133 patients for secondary prevention, there was no significant difference regarding the 2‐year appropriate ICD therapy rates (15.3% in the primary‐prevention group and 23.9% in the secondary‐prevention group, P=0.114).^37^ In the JID‐CAD registry of 392 patients with ischemic cardiomyopathy (165 for primary prevention and 227 for secondary prevention), the rate of appropriate ICD therapy in the primary‐ and secondary‐prevention groups was similar (P=0.576).^38^ An et al. also reported that the rate of appropriate ICD therapy was 37% at 3 years in patients with ischemic cardiomyopathy (LVEF ≤30%) who met the MADIT‐II inclusion criteria.^39^ Thus, in Japanese patients with ischemic cardiomyopathy, the rate of appropriate ICD therapy currently does not differ between primary and secondary prevention patients, and it is suggested that the use of ICDs for primary prevention in patients with ischemic cardiomyopathy may not be sufficient.

In the 2022 European Society of Cardiology (ESC) guidelines for the management of patients with ventricular arrhythmias and the prevention of SCD, primary‐prevention ICD therapy for heart failure patients with NYHA functional class II and LVEF ≤35% is recommended Class I, and the presence of NSVT is not included.^40^ On the other hand, NSVT has been reported as a predictor of SCD and a risk factor for appropriate ICD therapy. A previous meta‐analysis reported that the specificity of NSVT for predicting SCD in patients with heart failure with reduced ejection fraction (HFrEF) was as high as 89–97%.^41^ Makimoto et al. indicated that NSVT after ICD implantation was documented in 32% of patients with a primary‐prevention ICD, and those with documented NSVT had a higher risk of subsequent appropriate ICD therapy.^42^ In a Japanese multicenter observational prospective study (JANIES‐SHD, 66% ischemic heart disease), it was shown that low LVEF and documented NSVT on Holter ECG were independent predictors of fatal arrhythmic events.^43^.

Although the risk of fatal arrhythmias is clearly higher in patients with NSVT, the use of an ICD should be proactively considered, even in the absence of NSVT, in patients with ischemic cardiomyopathy and severely impaired left ventricular function.

### 2.4 Non‐Ischemic Cardiomyopathy (Table 3)

Late gadolinium enhancement (LGE) on cardiac magnetic resonance (CMR) and genetic testing have been reported as useful in risk stratification of fatal arrhythmias in non‐ischemic cardiomyopathy. In a meta‐analysis of 2,948 patients with non‐ischemic considering cardiomyopathy, LGE was present in 42% of patients with a primary‐prevention ICD, and the annual incidence of fatal arrhythmias was significantly higher in patients with LGE compared with those without LGE (17.2% vs. 2.1%, HR 7.8, P=0.007), but LGE did not correlate with LVEF (P=0.22).^44^ Furthermore, in a study of 1,020 patients with non‐ischemic cardiomyopathy, the occurrence of fatal arrhythmias was significantly associated with LGE on CMR, but there was no significant association between LVEF ≤35% and SCD.^45^ These results may indicate the limitations of using LVEF alone to predict the risk of fatal arrhythmias in non‐ischemic cardiomyopathy.

Regarding genetic testing, in a study of 487 individuals with non‐ischemic cardiomyopathy, pathogenic gene variants were identified in 37% of patients, and the patients with *LMNA*, a nuclear membrane lining protein, had a significantly higher risk of SCD and fatal arrhythmias.^46^.

In light of these findings, the 2022 ESC guidelines recommend an ICD in patients with non‐ischemic cardiomyopathy and left ventricular dysfunction (LVEF <50%) who have ≥2 of the following 4 risks: (1) syncope, (2) LGE on CMR, (3) monomorphic VT induced during electrophysiological study (EPS), and (4) pathogenic mutations of the *LMNA*/phospholamban [*PLN*]/filamin C [*FLNC*]/RNA binding motif protein 20 [*RBM20*] genes.^40^ It is important to consider LGE on CMR and genetic testing in assessing the risk of lethal arrhythmias when considering the indication for primary prevention ICD.

## 3 ICD INDICATIONS FOR CARDIAC SARCOIDOSIS

Sarcoidosis is a systemic inflammatory disease characterized by non‐caseating granulomas of unknown cause.^47^ Among the affected organs, pulmonary involvement is the most common, but cardiac involvement (cardiac sarcoidosis) is observed in ≈5% of patients, and cardiac involvement is responsible for about half of all deaths due to sarcoidosis.^48^, ^49^ In recent years, isolated cardiac sarcoidosis with lesions only in the heart^50^ and a poor prognosis^51^ as been reported, which has increased the importance of differential diagnosis.

### 3.1 Clinical Features of Cardiac Sarcoidosis

The clinical presentation of cardiac sarcoidosis is characterized by heart failure due to left ventricular dysfunction and SCD due to advanced atrioventricular block or fatal ventricular arrhythmias (VT/ventricular fibrillation [VF]).^52^ Complete atrioventricular block is often attributed to granulomatous inflammation,^53^ and VT/VF to scar formation due to tissue fibrosis, but it has been suggested that inflammation may directly cause VT/VF.^54^ Because inflammation is the main pathophysiology in cardiac sarcoidosis, the mainstay of treatment is immunosuppressive therapy, including steroids.^55^, ^56^ Therefore, even in cases in which an ICD is indicated, adequate immunosuppressive therapy should be administered.

Recent, relatively large‐scale epidemiological studies showed a high incidence of VT/VF and SCD in patients with cardiac sarcoidosis.^57^, ^58^ In a study of patients who received an ICD for primary prevention, VT/VF more frequently occurred early after ICD implantation in those with cardiac sarcoidosis compared with those having dilated cardiomyopathy^59^ In a multicenter retrospective study of 351 patients in Finland, SCD occurred in ≈14%, accounting for ≈80% of deaths.^57^ A multicenter retrospective study of 512 patients in Japan also reported an incidence of VT/VF or SCD of ≈20% in the first 5 years after diagnosis.^58^, ^60^.

### 3.2 Factors Associated With the Development of VT/VF

Typical factors associated with the development of VT/VF are (1) LVEF ≤35%,^60^, ^61^ (2) advanced atrioventricular block,^62^, ^63^ (3) delayed enhancement on the right or left ventricle in CMR,^64^, ^65^, ^66^, ^67^ and (4) residual myocardial inflammation. VT/VF and SCD may occur in patients with preserved LVEF, and in those patients, the usefulness of risk stratification by cardiac EPS,^68^, ^69^ delayed enhancement on CMR,^67^ cardiac^18^ F‐fluorodeoxyglucose‐positron emission tomography (^18^ F‐FDG‐PET) and 67 gallium (Ga) scintigraphy has been reported.^67^, ^70^, ^71^.

Mehta et al. investigated the prognostic value of EPS in 76 asymptomatic patients with histologically diagnosed extracardiac sarcoidosis and abnormal findings on cardiac^18^ F‐FDG‐PET and CMR. In 8 of the 76 (11%) patients, VT/VF was induced, and during follow‐up (median 5 years), 6 of 8 had VT/VF or SCD. In contrast, only 1 death occurred among the 68 patients in whom VT/VF was not induced.^72^ Therefore, it is reasonable to use an EPS for risk stratification.

### 3.3 Usefulness of ICD for Cardiac Sarcoidosis

There are many reports on the usefulness of ICDs for cardiac sarcoidosis, both for primary and secondary prevention.^73^, ^74^, ^75^ ICD implantation for primary prevention is particularly useful in patients with LVEF ≤35%,^61^, ^73^ and is strongly recommended in the guidelines (Class I Recommendation).^40^, ^76^ In addition, as noted earlier, the risk of VT/VF or SCD is significantly increased in cardiac sarcoidosis patients with advanced atrioventricular block, indicating the need for a permanent pacemaker (especially when NSVT is present^77^) or when CMR shows a large extent of delayed enhancement^67^, ^74^, ^78^ and an ICD should be considered for these patients.^40^, ^76^.

Recently, quantitative evaluation of delayed enhancement in CMR has become possible, and its usefulness in predicting VT/VF and SCD has been reported.^65^, ^79^, ^80^ However, no standard quantitative methods with adequate consensus have been established. Among the studies of risk stratification using CMR, most studies defined a large extent of delayed enhancement for high risk of adverse events as >20% of myocardial weight, even after inflammation was controlled by immunosuppressive therapies.^58^, ^65^, ^79^, ^80^.

Regarding^18^ F‐FDG‐PET and 67Ga scintigraphy, not only are there reports suggesting their usefulness in risk stratification,^70^, ^71^, ^81^ but also negative reports,^82^ making it difficult to assess the risk using these modalities alone. In particular,^18^ F‐FDG‐PET imaging is difficult to use for judging the indication for ICD implantation as primary prevention, because the standardized uptake value (SUV) varies widely among centers and is difficult to quantify.

However, patients with right ventricular FDG accumulation or residual inflammation in abnormal areas on perfusion scintigraphy are at particularly high risk,^81^ and in patients with residual inflammation that has been determined to be refractory to immunosuppressive drugs, risk assessment for SCD should be performed by cardiac function, delayed enhancement on CMR, and EPS. Kazmirczak et al.^83^ validated these risk factors in a retrospective cohort of 290 patients using the American College of Cardiology (ACC)/American Heart Association (AHA)/Heart Rhythm Society (HRS) guidelines. The guidelines were validated with an annual incidence of SCD or VT/VF ranging from 19.4% to 81.7% and from 2.1% to 19.6%, respectively, in patients with Class I or IIa ICD indication. In a Japanese retrospective cohort of 188 patients, 3.9–6.8% and 2.4–2.5% per year of SCD or VT/VF, respectively, occurred in patients assigned a Class I or IIa Recommendation for ICD indication in the ACC/AHA/HRS guidelines, generally supporting the validity of the guidelines.^84^.

**FIGURE 1:**
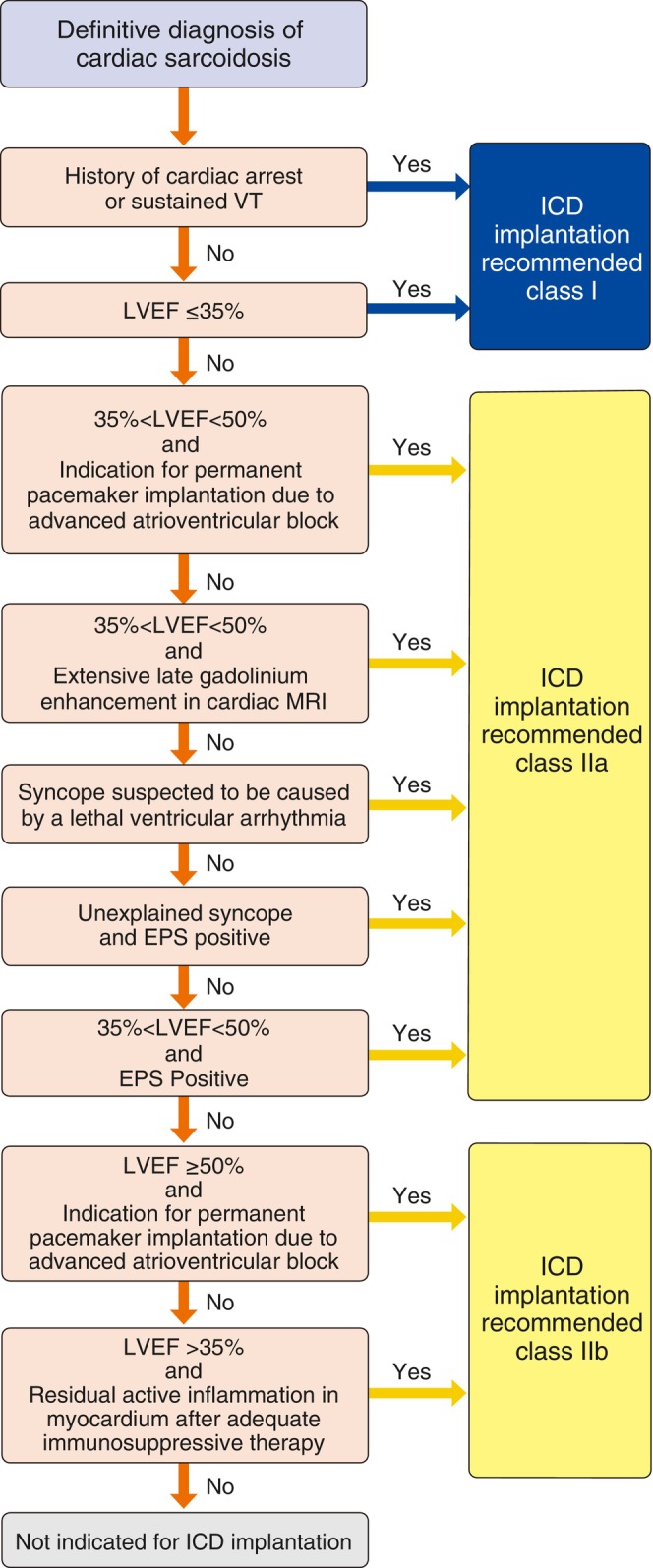
Algorithm for ICD implantation. CMR, cardiac magnetic resonance imaging; EPS, electrophysiological study; ICD, implantable cardioverter defibrillator; LVEF, left ventricular ejection fraction; VF, ventricular fibrillation; VT, ventricular tachycardia.

Therefore, the evidence‐based decision on the indication for ICD treatment in Japanese patients with cardiac sarcoidosis is basically consistent with the European and American guidelines. **Table** **4** shows the recommendations and level of evidence for ICD for cardiac sarcoidosis, and **Figure** **1** shows the algorithm for determining the indication for ICD based on the recommendations.

**TABLE 4:** Recommendations and Levels of Evidence for ICD Treatment in Cardiac Sarcoidosis.

	COR	LOE
ICD implantation is recommended in patients with history of cardiac arrest or sustained VT	I	B
ICD implantation is recommended in patients with LVEF ≤35%	I	B
ICD implantation (CRT‐D) should be considered in patients with 35%<LVEF<50% and with an indication for permanent pacing due to advanced AV block	IIa	B
ICD implantation should be considered in patients with 35%<LVEF<50% and with large extent of LGE on CMR	IIa	B
ICD implantation should be considered in patients with syncope possibly caused by lethal ventricular arrhythmias (e.g., VT/VF)	IIa	B
ICD implantation should be considered in patients with unexplained syncope and sustained VT or VF was induced in an EPS	IIa	B
ICD implantation should be considered if 35%<LVEF<50% and sustained VT or VF was induced in an EPS	IIa	C
ICD implantation may be considered in patients with LVEF ≥50% and with an indication for permanent pacing due to advanced AV block	IIb	C
ICD implantation may be considered if LVEF >35% and cardiac PET or gallium scintigraphy shows residual active inflammation after adequate immunosuppressive therapy, including steroids	IIb	C

AV, atrioventricular; CMR, cardiac magnetic resonsnce; COR, Class of Recommendation; CRT‐D, cardiac resynchronization therapy defibrillator; EPS, electrophysiological study; ICD, implantable cardioverter defibrillator; LGE, late gadolinium enhancement; LOE, Level of Evidence; LVEF, left ventricular ejection fraction; PET, positron emission tomography; VT/VF, ventricular tachycardia/ventricular fibrillation.

Some patients with cardiac sarcoidosis cannot be evaluated by CMR because of permanent pacemaker implantation, intrabody metallic devices, or renal impairment. Although the evidence is not sufficient for recommendation, fragmented QRS on 12‐lead ECG,^85^, ^86^ the T‐peak to T‐end interval to QT interval ratio,^87^ and thinning of the ventricular septal base on echocardiography have been reported as significantly associated with the risk of VT/VF.^88^ Notably, Nordenswan et al. showed that the 5‐year incidence of SCD in patients with cardiac sarcoidosis who do not meet the US guideline recommendations for a Class I or IIa indication is relatively high at 4.8% (95% confidence interval [CI] 1.2‐19.1),^89^ and more precise risk stratification is warranted.

## 4 LEADLESS PACEMAKER

**FIGURE 2:**
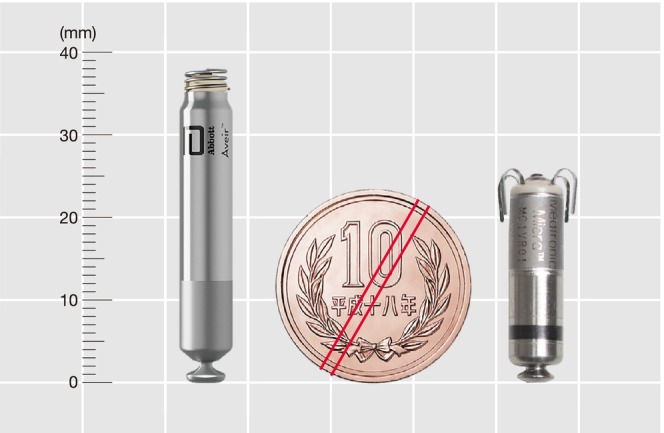
Leadless pacemakers available in Japan (as of November 2023). (**Left**) VVI type, (**Right**) VVI or VDD type.

**FIGURE 3:**
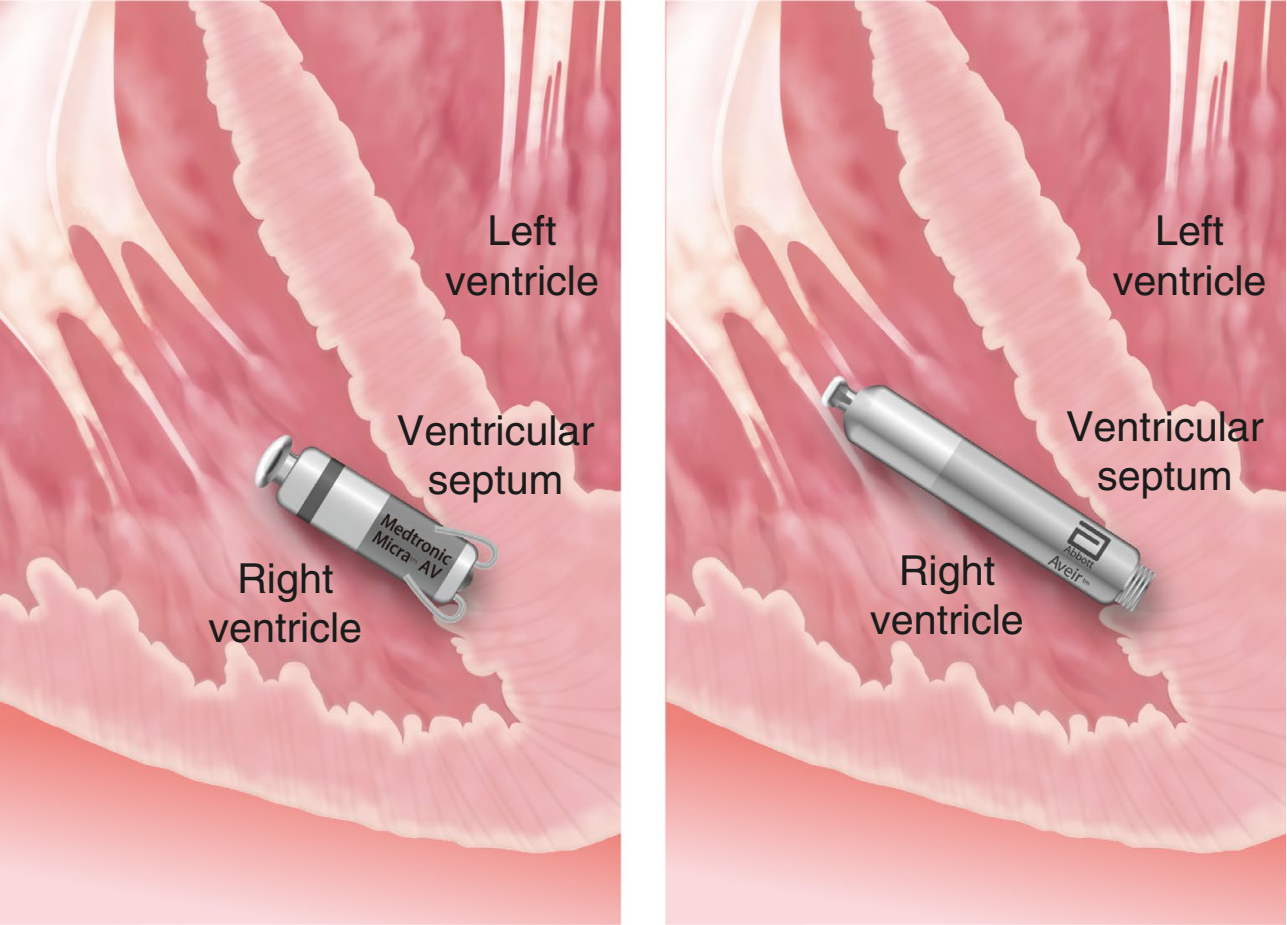
Schematic diagram of leadless pacemaker implantation in the heart.

The indications for leadless pacemakers (**Figures** **2**,**3**) were discussed in the 2021 JCS/JHRS Guideline Focus Update for Non‐pharmacologic Treatment of Arrhythmias^6^ regarding venous obstruction and stenosis, and the need for preservation of venous access. Since then, the indications for leadless pacemakers have continued to expand, and various evidences have emerged. This Focus Update describes the new models and modes that have become available, as well as new findings on efficacy and safety. Recommendations for leadless pacemaker implantation are listed in **Table** **5**.

**TABLE 5:** Recommendations and Levels of Evidence for Leadless Pacemaker Implantation.

	COR	LOE
Implantation of a leadless pacemaker is recommended for patients with the following conditions: (1) high risk of infection, (2) endstage renal failure, (3) history of device infection, (4) anatomic cause of difficulty in transvenous lead implantation such as congenital heart disease, (5) on drug therapy such as steroids or immunosuppressive drugs, (6) under radiation therapy, (7) under long‐term intravascular catheter placement or a past history of long‐term intravascular catheter placement.	I	B
Evaluation of the following risk factors for cardiac perforation and effusion is recommended: (1) age ≥85 years, (2) BMI <20 kg/m^2^, (3) female sex, (4) HF, (5) old myocardial infarction, (6) pulmonary hypertension, (7) COPD, and (8) dialysis	I	B

BMI, body mass index; COPD, chronic obstructive pulmonary disease; COR, Class of Recommendation; HF, heart failure; LOE, Level of Evidence.

### 4.1 Applicable Age

Leadless pacemakers have tended to be used primarily in patients with bradycardic atrial fibrillation and older patients due to the difficulty in achieving atrioventricular synchronization such as VVIR or VDDR modes (Micra^TM^ AV). However, concerns that transvenous pacemaker implantation in younger patients may result in prolonged exposure to infection risk and that high activity may increase the risk of lead damage have led to a reconsideration of the usefulness of leadless pacemakers in these populations.

Although no conclusions have been reached regarding the management of leadless pacemaker at the end of life (i.e., whether the pacemaker should be removed at the time of new implantation), 78% of experts in the UK Expert Consensus^90^ said that leadless pacemaker implantation in patients aged <40 years is reasonable. Patients recommended for leadless pacemaker implantation include those at high risk of infection, endstage renal failure, with a history of device infection, anatomic constraints that prevent transvenous lead implantation, on medications such as steroids or immunosuppressive drugs, under radiation therapy, congenital heart disease, <40 years of age, and with or in need of an intravascular catheter.

In a multicenter, retrospective, observational study of 35 patients aged 18–40 years, the safety endpoint (no major complications attributable to the system or procedure) was achieved 100% of the time with an average of 26 months of observation and was stable at a pacing threshold of <2 V at 0.24 ms pulse width. The efficacy endpoint (threshold increase ≤1.5 V from the time of implantation) was 94%.^91^.

Although implantation of a leadless pacemaker in younger patients is expected to expand in the future, the 2021 ESC guideline comments that careful decisions should be made for patients whose life expectancy is expected to exceed 20 years.^92^ The possibility of multiple implantations and the decision at the time of battery depletion (i.e., choice between removal and turning it off, etc.) should be considered.

### 4.2 Safety

The Micra CED study used U.S. Medicare data to evaluate the mid‐term outcomes at 3 years after implantation of the Micra^TM^ VR (6,219 patients) and transvenous VVI leadless pacemaker (10,212 patients). Compared with transvenous VVI pacemakers, the Micra^TM^ VR had a similar all‐cause mortality rate (HR 0.97, 95% CI 0.92–1.03), but lower rates of remote complications (HR 0.68, 95% CI 0.59–0.78), device re‐intervention (HR 0.59, 95% CI 0.44‐0.78), infection (<0.2% vs. 0.7%, P<0.0001), and heart failure hospitalization. A significant reduction in the heart failure hospitalization rate was found (HR 0.90, 95% CI 0.84–0.97).^93^ These results indicate that the benefits of leadless pacemakers are maintained in the medium term perspective.

### 4.3 New Leadless Pacemaker

The Aveir^TM^ VR (Abbott) was approved in Japan in December 2022 and implantation has begun. This leadless pacemaker, an improvement on its predecessor Nanostim^TM^, is characterized by the ability to measure electrical information before fixation and the possibility of upgrading to DDD in the future (DDD is not approved in Japan as of January 2024). In a prospective DDD leadless study of 300 patients published in June 2023, the implantation success rate was 98.3%, device‐ and procedure‐related complications (e.g., intraoperative and postoperative dislodgement in 1.7% and 0.7% of patients, respectively) were ≈9.7%, and >70% atrioventricular synchronization at 3 months after implantation was observed in 90.2% of patients.^94^ The Aveir^TM^ VR was implanted in 200 patients in LEADLESS‐II Phase 2, and a mean follow‐up of 3.9 months showed that both safety (96.0%, 95% CI 92.2–98.2%) and efficacy endpoints (95.9%, 95% CI 92.1–98.2%) were favorable. The most common complications were tamponade (3 patients, 1.5%; open chest in 2 cases) and incomplete implantation (1.5%), which were considered generally similar to those for Micra^TM^.^95^ For Nanostim^TM^, the predecessor of Aveir^TM^, an extraction system was available and it has been reported that extraction is possible up to 9 years after implantation.^96^ However, the degree of difficulty of removal after long‐term implantation has not been fully investigated, leaving many aspects still unknown.

### 4.4 VDD Mode

The Micra^TM^ AV uses an accelerometer to sense atrial contraction and enables atrial‐synchronized ventricular (VDD) pacing. The MARVEL2 study of patients with normal sinus node function and complete atrioventricular block showed a higher rate of synchronized atrioventricular pacing in the VDD mode compared with VVI mode, but the evaluation was limited to relatively short periods of time at rest.^97^.

In the Accel AV study, 152 patients with atrioventricular block were implanted with the Micra^TM^ AV, and their atrioventricular synchrony in daily life was evaluated at 1 month. The VDD mode of the Micra^TM^ AV demonstrated an average of 85.4% atrioventricular synchrony at rest and improved quality of life.^98^ On the other hand, atrioventricular synchrony during activity was low at 74.8%, but careful reconfiguration resulted in an additional improvement of >10%. The fusion of A3 the period between ventricular diastole and A4 (atrial contraction) due to increased heart rate, and decreased sensing of atrial contraction due to body movement were considered to be responsible for loss of atrioventricular synchrony. In highly active patients, patients with atrioventricular block and preserved sinus function, and in patients with atrial dysfunction, Micra^TM^ AV may decrease the rate of atrioventricular synchrony. Optimization of the outpatient program setting is considered important once patients return to daily activities.^99^.

### 4.5 Risk Assessment of Complications

Leadless pacemakers have no lead or pocket‐related complications than transvenous pacemakers, but myocardial perforation and pericardial effusion occur in 1–2% of cases.^100^, ^101^ Preoperative risk assessment is important because some patients require open chest surgery for myocardial perforation.

Piccini et al.^102^ collected data on 2,817 patients from 3 international clinical trials of Micra^TM^, and validated a risk score for pericardial effusion on implantation in 32 patients with pericardial effusion. Among many clinical characteristics, the investigators ultimately determined that age >85 years, BMI <20 kg/m^2^, female sex, heart failure, old myocardial infarction, pulmonary hypertension, chronic obstructive pulmonary disease (COPD), and dialysis were risk enhancing factors, and age <85 years, atrial fibrillation, post open heart surgery, and coronary artery disease were risk‐reducing factors. They developed a risk score with 1 point for enhancing factors (2 points for COPD) and minus 1 point for reducing factors, and found that intermediate‐risk (1 point) and high‐risk (2 or more points) patients had significantly more pericardial effusions (0.4%, 1.5%, and 4.8% predictive value for each) than low‐risk (0 point) patients. This high‐risk group of patients should be especially cautioned because repeated Micra^TM^ deployments increase the risk of pericardial effusions.

In the Micra VR Acute Performance registry in Japan that includes 300 patients, major complications at 1 and 6 months postoperatively were similar to those in the international registry, but Japanese patients were older, had lower BMI, were more likely to be female, and had more risk factors for pericardial effusion.^103^.

### 4.6 Leadless Pacemaker as a Replacement After Device Removal

There have been several reports on leadless pacemaker implantation as an alternative to temporary pacing in pacing‐dependent patients after device removal due to infection. Beccarino et al.^104^ reported the results of 86 patients, including 65 patients with bacteremia, who underwent device removal and simultaneous leadless pacemaker implantation. The patients were followed for 163 postoperative days, and there was no recurrence of infection. They reported that 25 deaths (29%), 88% of which were not causally related to infection; however, 9 patients had methicillin‐resistant *Staphylococcus aureus* (MRSA) or *Candida* infection, and 3 patients had persistent infection despite lead removal.

Breeman et al. performed leadless pacemaker implantation before (4 patients), simultaneously (5 patients) or after (20 patients) device removal. During the 32 months of follow‐up, no cases of re‐infection were observed, but bleeding from the femoral artery occurred in 2 patients.^105^ Bicong et al. also analyzed 39 patients who underwent leadless pacemaker implantation after device removal due to infection, and reported no cases of re‐infection but 3 of complications (puncture site hematoma, femoral arteriovenous fistula, and pacemaker syndrome) after a mean follow‐up of 2 years.^106^.

A multicenter study comparing leadless pacemaker implantation after device removal (184 patients) with initial leadless pacemaker implantation (995 patients) found no significant differences in implantation‐related complications (1.6% in the lead removal group vs. 2.2% in the initial implantation group) or all‐cause death (5.4% vs. 7.8%, respectively) during a 33‐month follow‐up period.^107^.

Implantation of a leadless pacemaker after device removal may be useful, but further studies are needed to determine long‐term outcomes.

**PQ 1. How to Choose Between a Leadless Pacemaker and a Transvenous Pacemaker**

As of October 2023, there are 2 types of leadless pacemakers approved in Japan: the VVI type (Micra^TM^ VR: tine type; Aveir^TM^ VR: screw type) and the VDD type (Micra^TM^ AV). The VVI and VDD types have been implanted mainly in patients with bradycardia and atrial fibrillation, and in older patients. However, leadless pacemakers are now being reconsidered for use in younger patients to reduce or avoid the risk of device infection, lead damage, and venous obstruction. The decision for indication is often based on (1) venous access and infection risk, (2) the need for atrial pacing, and (3) the need for high atrioventricular synchrony.^90^.

Leadless pacemakers are implanted in the right ventricle, eliminating the need for intravenous leads or anterior thoracic subcutaneous pockets, which eliminate the most common causes of pacemaker complications. The Micra Coverage with Evidence Development (CED) Study using U.S. Medicare data showed a 30% reduction in complications over 3 years for leadless pacemakers.^93^ The risk of infection is thought to be reduced because the leadless pacemaker itself is implanted in the right ventricle, where blood flow is rapid, and is endothelialized relatively early, plus the surface area of the leadless pacemaker is much smaller than that of a venous lead implanted in a slow‐flowing vein. Therefore, the risk of infection is considered to be reduced in patients treated with steroids or immunosuppressive drugs, on hemodialysis, with a history of device infection, with congenital heart disease or with a narrowed or obstructed subclavian vein for any reason, those undergoing radiation therapy, and patients with an implanted or planned endovascular catheter or subcutaneous port. Note that Micra is considered difficult to remove due to endothelialization.

VVIR and VDD are the currently available modes for leadless pacemakers in Japan, neither of which can provide atrial stimulation. Transvenous pacemakers are recommended for patients who require atrial stimulation or atrioventricular synchronization, the benefits of which outweigh the risks of atrial lead insertion, or for those who require conduction system pacing. The limitation of current systems is the loss of atrioventricular synchrony during exercise in highly active patients with tachycardia >115 beats/min. In particular, a transvenous pacemaker is recommended for the treatment of atrioventricular block during exercise, because, at present, leadless pacemakers do not maintain atrioventricular synchronization, and the possibility of pacemaker syndrome cannot be ruled out. The battery life of current pacemakers is approximately 12 years, but next‐generation pacemakers are expected to have a longer life. There are overseas reports of leadless implantation in children as a bridge to future transvenous pacemakers without the risk of lead breakage or venous obstruction, and future changes in indications are anticipated.

## 5 PACEMAKER THERAPY FOR REFLEX SYNCOPE

Pacemaker therapy for reflex syncope is recommended in Japan for patients aged ≥40 years with documented long cardiac arrest (>3 s symptomatic, >6 s asymptomatic) and when other therapies such as counterpressure maneuver and orthostatic training are ineffective.^5^.

Recently, the efficacy of a dual‐chamber pacemaker with a closed loop stimulation sensor (DDD‐CLS) in preventing recurrent syncope in patients with recurrent cardioinhibitory reflex syncope has been reported. The DDD‐CLS works with an algorithm that estimates myocardial contractility from changes in intracardiac impedance caused by right ventricular leads and adjusts the pacing rate.

A small, randomized open trial confirmed the efficacy of DDD‐CLS in reducing recurrent syncope,^108^, ^109^ and a double‐blind study reported that DDD‐CLS reduced recurrent syncope and prolonged the time to first syncope^110^, ^111^ and improved quality of life (QOL).^112^ In a retrospective study with 5‐year follow‐up, DDD‐CLS significantly reduced the risk of syncope compared with physiotherapy.^113^ A multicenter study of the head‐up tilt test after DDD pacemaker implantation showed that DDD‐CLS reduced syncope and hypotension caused by the head‐up tilt test compared with DDD.^114^ It is thought that the CLS sensor increases heart rate and maintains cardiac output from the early phase of reflex syncope, preventing syncope.

Based on the current evidence, this Focus Update recommends DDD‐CLS pacemaker therapy as recommended Class IIa in patients aged ≥40 years with recurrent cardioinhibitory syncope who have undergone a head‐up tilt test and demonstrated cardiac cardioinhibitory syncope. The long‐term results are unknown, and a large‐scale study is desirable in the future. Because the Head Up Tilt Study did not demonstrate the efficacy of conventional pacemakers in preventing reflex syncope with hypotensive reactions,^115^ we continue to recommend Class III as before (**Table** **6**).

**TABLE 6:** Recommendations and Levels of Evidence for Pacemaker Therapy for Reflex Syncope.

	COR	LOE
DDD‐CLS pacemaker should be considered for patients aged ≥40 years with recurrent cardioinhibitory reflex syncope who demonstrate cardioinhibitory response in the head‐up tilt test	IIa	C
DDD pacemaker therapy may be considered for patients aged ≥40 years with recurrent reflex syncope, with ECG evidence of cardioinhibitory spontaneous syncope (>3 s of cardiac arrest with symptoms, >6 s of cardiac arrest without symptoms), and in whom other treatment options have failed	IIb	C
Pacemaker therapy is not recommended for reflex syncope patients aged <40 years	III (No benefit)	C
Pacemaker therapy is not recommended for patients aged ≥40 years without ECG evidence of syncope, and a definite diagnosis of cardioinhibitory type cannot be made	III (No benefit)	C
Pacemaker therapy without CLS function is not recommended for patients with reflex syncope, age ≥40 years, without ECG documentation of spontaneous syncope, and vasopressor type on head‐up tilt test	III (No benefit)	C

COR, Class of Recommendation; DDD‐CLS, dual‐chamber pacemaker with a closed loop stimulation sensor; ECG, electrocardiogram; LOE, Level of Evidence.

## 6 FUTURE ADVANCES IN IMPLANTABLE CARDIAC ELECTRICAL DEVICES

Although a secondary analysis of PRAETORIAN showed that subcutaneous ICDs (S‐ICDs) reduce lead‐related complications by 30% compared with transvenous ICDs,^116^ the inability of S‐ICDs to provide pacing for bradycardia and antitachycardia pacing for VT has led some patients to abandon S‐ICD implantation. Recently, a solution was developed by combining an S‐ICD with a dedicated leadless pacemaker. With this system, when antitachycardia pacing is ineffective, defibrillation is performed by the S‐ICD. Animal studies have reported good communication between the S‐ICD and the leadless pacemaker, as well as the success rates of antitachycardia pacing.^117^, ^118^, ^119^.

A multicenter, prospective, single‐arm study in humans is ongoing as of February 2024, and results on the safety and efficacy of treatment with a combined S‐ICD and leadless pacemaker are expected to be evaluated.

The S‐ICD is recommended Class I in Japan for patients who are eligible for transvenous ICD implantation, have difficult venous access or are at high risk for infection and do not require bradycardia pacing, antitachycardia pacing for VT or CRT.^5^ In addition to the S‐ICD, an extravascular ICD (EV‐ICD) with a substernal lead has been developed and is undergoing clinical trials in Japan as of May 2023. However, it is not suitable for patients who require continuous pacing because the pacing threshold is higher than that of transvenous ICDs. When placing a lead under the sternum, its position should be confirmed by multidirectional fluoroscopic imaging to avoid myocardial injury and pneumothorax. Because the lead has 2 coils and 2 ring electrodes, multiple sensing and pacing vectors can be selected.

In a multicenter prospective single‐arm study (316 patients),^120^ the success rate of defibrillation during EV‐ICD implantation was 98.7% (median energy 15 J) with no intraoperative complications. The success rate of antitachycardia pacing was 50.8%. Complications at 6 months after implantation were hematoma, infection, pain, wound dehiscence, lead migration, and inappropriate therapy in 7.3% of patients. Inappropriate therapy occurred in 29 patients, with P‐wave oversensing being the most common.^121^ In unsuccessful defibrillation cases, studies analyzing CT images have suggested anatomic factors such as a large rib cage width, myocardium extending very posteriorly, and a caudal heart position in the chest, but multivariate analysis showed no significant differences.^122^ Further studies on EV‐ICDs are needed to accumulate evidence.

## 7 CONDUCTION SYSTEM PACING (CSP)

**FIGURE 4:**
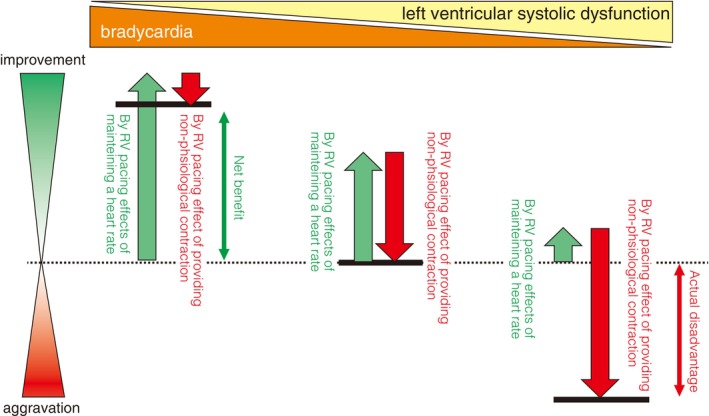
Benefits and disadvantages of right ventricular (RV) pacing. When bradycardia is the primary pathology, the induction of dyssynchronous contractions by RV pacing does not provide a major problem because the rate‐maintaining effect of pacing is large. On the other hand, when left ventricular systolic dysfunction is the main pathology, dyssynchronous contractions (the “harmful effect”) induced by RV pacing greatly outweigh the heart rate maintenance effect and worsen the heart failure. (**Vertical axis**) Improvement or worsening of the patient's condition with RV pacing; (**Horizontal axis**) Ratio of bradycardia to left ventricular systolic dysfunction in the patient's condition (bradycardia should predominantly be present on the left and systolic dysfunction on the right); (**Black horizontal line**) Net benefit or disadvantage of RV pacing.

When bradycardia is the primary pathology, hemodynamic improvement is delivered predominantly by heart rate maintenance; thus, dyssynchronous contraction (the “harmful effect”) by right ventricular apical pacing (RVP) is unlikely to be a major concern. In contrast, when left ventricular systolic dysfunction coexists, dyssynchronous contractions induced by RVP greatly outweigh the benefit of heart rate maintenance, resulting in a worsening of the condition (**Figure** **4**). Substantial RVP (pacing burden >20–40%) has been reported to increase cardiovascular events such as deterioration of LVEF and heart failure hospitalization.^122^, ^123^, ^124^ Right ventricular high septal pacing, which captures the myocardium closer to the conduction system, has been attempted as an alternative to RVP, but did not protect left ventricular function.^125^.

Pacing‐induced cardiomyopathy, a condition in which LVEF decreases over time under RVP, occurs in 12–20% of patients after pacemaker implantation.^126^ Previous studies demonstrated that a higher pacing burden, paced QRS duration >160 ms, and low preoperative LVEF were risk factors for pacing‐induced cardiomyopathy, especially in patients with mild‐to‐moderate LV dysfunction.^127^, ^128^.

**FIGURE 5:**
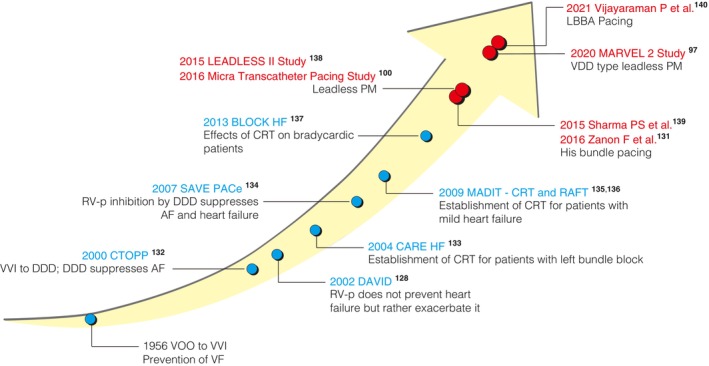
History of cardiac pacing development. The history of the development of cardiac pacing over the past 60 years is presented. Originally developed for patients with bradycardia, cardiac pacing has now expanded to include the treatment of heart failure in patients with systolic dysfunction. Although there are other clinical trials that have had a similar impact, this figure focuses on representative large clinical trials. Red circles: Articles referred to in preparing this Focus Update. AF, atrial fibrillation; CRT, cardiac resynchronization therapy; LBBA, left bundle branch area; PM, pacemaker; VF, ventricular fibrillation.

His bundle pacing (HBP), which directly captures the conduction system rather than the local myocardium, was expected to retain the physiological activation pattern in animal models^129^ and clinical cases.^130^ However, the low procedural success rate of HBP remains a major issue.^131^ In recent years, a delivery catheter system for implantation of a lead has become available, resulting in an increase in the procedural success rate. The clinical efficacy of CSP has gradually become evident, and not only HBP but also left bundle branch area pacing (LBBAP) is again attracting attention^97^, ^100^, ^128^, ^131^, ^132^, ^133^, ^134^, ^135^, ^136^, ^137^, ^138^, ^139^, ^140^ (**Figures** **4**,**5** and **Table** **7**).

**TABLE 7:** Recommendations and Levels of Evidence for CSP.

	COR	LOE
**Indication for CSP for bradyarrhythmias**		
CSP should be considered for patients with an indication for permanent pacing and with mild to moderate left ventricular dysfunction (LVEF 35–50%) in whom substantial ventricular pacing (>20%) is anticipated	IIa	C
CSP may be considered to avoid pacing‐induced cardiomyopathy for patients with an indication for permanent pacing and with normal left ventricular function in whom substantial ventricular pacing (>20%) is anticipated	IIb	C
CSP may be considered for patients requiring AV junction ablation	IIb	C
**Indications for CSP as an alternative therapy to CRT**		
CSP should be considered for patients with an indication of CRT due to LBBB or substantial ventricular pacing when CRT is ineffective or cannot be established for any reason	IIa	C

AV, atrioventricular; COR, Class of Recommendation; CRT, cardiac resynchronization therapy; CSP, conduction system pacing; LBBB, left bundle branch block; LOE, Level of Evidence; LVEF, left ventricular ejection fraction.

### 7.1 Definition of CSP

CSP is a pacing technique that captures the conduction system (His bundle, right bundle branch, left bundle branch, and left bundle branch fascicles), and is characterized by output‐dependent changes in QRS morphology due to the different pacing thresholds between the conduction system and local myocardium.

HBP captures the His bundle directly by implanting a lead on the atrial or ventricular side of the tricuspid annulus, providing the most physiological activation. Selective HBP, or non‐selective HBP, which captures the His bundle and local myocardium simultaneously, occurs when varying the pacing output.

LBBAP is a novel pacing technique achieved by deployment of a lead deep into the right ventricular septum that intends to capture the left bundle branch or fascicles beneath the left ventricular septal endocardium. Unlike HBP, the left bundle branch potential is not always evident. LBBAP is characterized by the presence of R waves in the terminal portion of lead V_1_, indicating delayed activation of the right ventricle.

The currently recognized criteria for direct left bundle branch capture are: output‐dependent changes in QRS morphology, the interval from LBB potential to V_6_RWPT (R‐wave peak time in lead V_6_) equals the interval from pacing stimulus to V_6_RWPT (±10 ms),^141^ the interval from pacing stimulus to V_6_RWPT is <75 ms (in patients with narrow QRS or isolated right bundle branch block) or <80 ms (in patients with more advanced ventricular conduction system disease),^142^ and V_6_–V_1_ interpeak interval is >44 ms.^143^ In addition, an abrupt decrease in the interval from pacing stimulus to V_6_RWPT >10 ms by varying pacing output is a helpful maneuver to distinguish between nonselective LBBAP (simultaneous capture of left bundle branch and left ventricular septum) and left ventricular septal pacing.^144^ However, it is still unclear whether the long‐term prognosis differs between left bundle branch capture and left ventricular septal pacing.

Currently, left ventricular septal pacing without direct left bundle branch capture is also categorized as LBBAP.^142^, ^145^.

### 7.2 Features and Differences Between HBP and LBBAP

**FIGURE 6:**
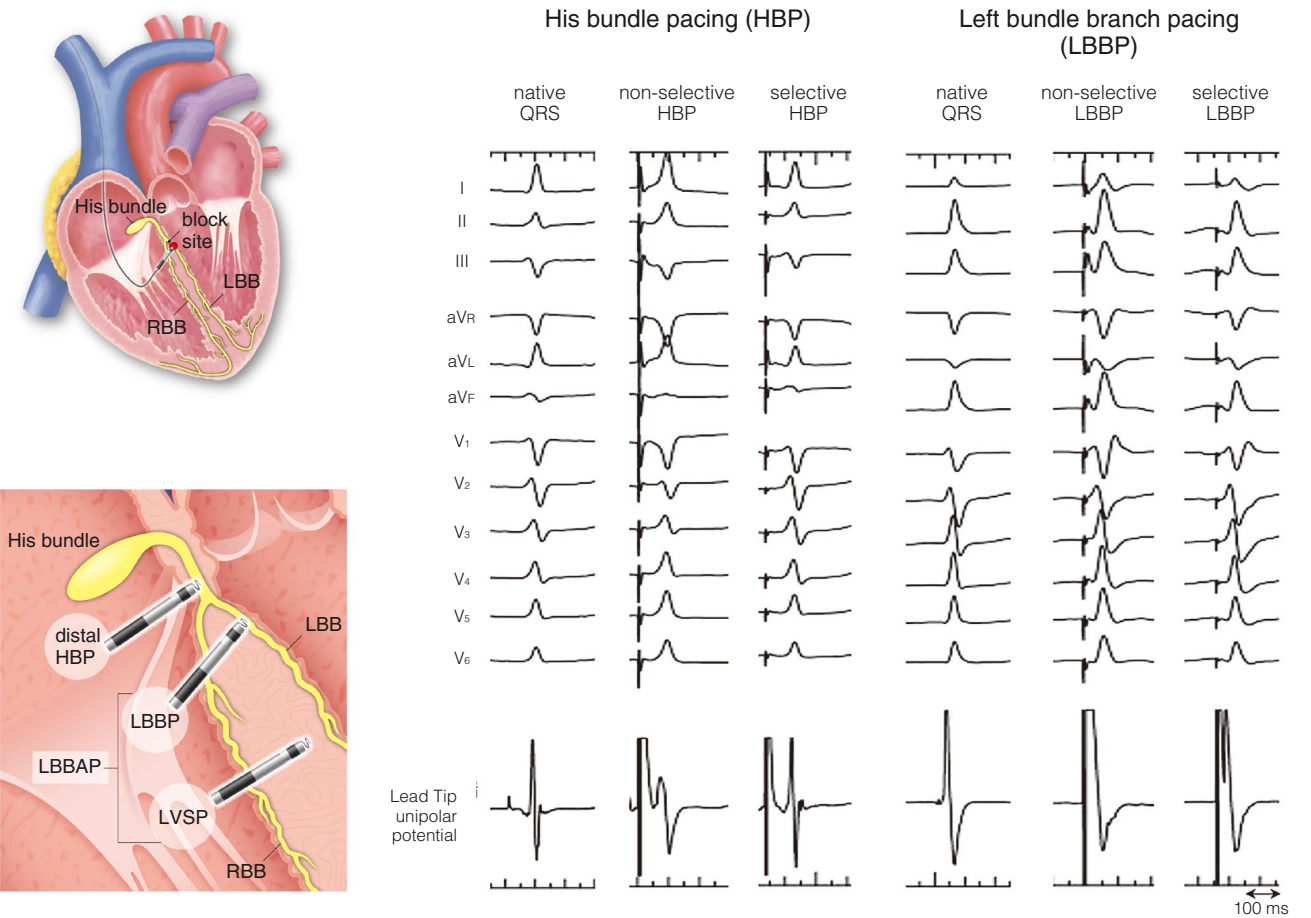
Schematic diagrams of pacing sites in conduction system pacing (**Left panel**) and 12‐lead QRS morphologies of nonselective and selective pacing of the conduction system (**Right panel**). In non‐selective pacing, the conduction system (His bundle or left bundle branch) and the local myocardium are captured simultaneously, whereas in selective pacing, the conduction system is captured directly.

Although HBP is the most physiologic pacing technique, the target area for HBP lead placement is narrow, and the lead should be located distal to the conduction block site (**Figure** **6**). Therefore, implementing HBP is generally considered a difficult procedure, but it does preserve not only left ventricular but also right ventricular physiologic activation.^146^ Previous study has shown that HBP shortens the QRS duration and improves LVEF even in patients with right bundle branch block.^147^ The introduction of a delivery catheter system for HBP has improved the procedural success rate by almost 92%.^131^ However, sensing failure (oversensing of atrial potentials and undersensing of ventricular potentials) and increased capture thresholds in the early and remote postoperative periods, and the need for lead replacement (7–11%) are still major concerns with HBP.^148^, ^149^, ^150^ Thus, additional leads for backup pacing may be indicated when HBP is attempted in pacing‐dependent patients.^92^, ^145^, ^146^, ^147^, ^148^, ^149^, ^150^.

LBBAP is characterized by a wider target area for lead implantation, high ventricular R wave, and low capture threshold, superior to HBP. Left ventricular activation time by LBBAP is comparable to that by HBP,^146^ and the procedural success rate of LBBAP is relatively high at almost 90–98%.^151^, ^152^, ^153^, ^154^.

In a Japanese initial multicenter observational study of LBBAP implantation for bradycardia, the success rate was 86.7%. The study reported that the presence of interventricular septal thickness >11 mm, intraventricular conduction disturbance, and severe tricuspid regurgitation were predictors of implantation failure.^155^ Furthermore, the success rate of HBP and LBBAP in patients with an indication of heart failure was 10–20% lower than that in patients with an indication of bradycardia. Therefore, evaluation of cardiac morphology and pacing indication is important for procedural success.^153^, ^154^, ^155^, ^156^, ^157^.

LBBAP has specific complications such as interventricular septal perforation, intraseptal hematoma, and coronary artery injury. In a European multicenter registry, LBBAP‐related complications occurred in 8.3% of patients, although the number of patients who required invasive intervention was limited.^153^ In a Japanese multicenter study, perioperative LBBAP‐related complications were observed in 4.0% of patients (all with interventricular septal perforation), but therapeutic intervention was not required and lead re‐implantation was safely achieved in all patients.^155^ When performing LBBAP, it is important to understand the specific behavior of the lead associated with deep deployment in the interventricular septum,^158^ changes in the unipolar signal on the distal electrode,^159^, ^160^ lead parameters throughout the follow‐up, and possible complications.

### 7.3 Systems Used for CSP

A thin (4.1Fr) lumenless lead (SelectSecure^TM^ lead), with the unique structure of an exposed helix electrode, is most commonly used for CSP implantation. Meanwhile, several studies have shown that procedural outcomes of CSP using stylet‐driven leads were equivalent or superior to lumenless leads.^153^, ^161^ On the other hand, a single‐center observational study showed that using stylet‐driven leads resulted in significantly higher postoperative loss of left bundle branch area capture (32% vs. 12%) compared with lumenless leads.^162^ Fractures of the helix electrode and distal conductor have also been reported for LBBAP using stylet‐driven leads.^163^, ^164^.

Regarding the extraction of CSP leads, it was reported that lumenless leads were extracted with a high success rate (97%) at mid‐term follow‐up (25±18 months) after HBP implantation.^165^ On the other hand, there are limited reports of LBBAP lead extraction,^166^ so the safety of extraction of the LBBAP lead located deeply in the septum for a long time is unclear and needs further evaluation, regardless of the lead design. It should be noted that LBBAP using either lumenless or styled‐driven leads is not covered by Japan's National Health Insurance as of February 2024.

### 7.4 Indications for CSP in Patients With Bradycardia

#### 7.4.1 Patients With Bradycardia and Normal Cardiac Function

Several observational studies have evaluated the benefit of CSP for patients with bradycardia and normal cardiac function in whom substantial ventricular pacing is anticipated. Early reports have shown that CSP reduced heart failure hospitalizations compared with conventional RVP in patients with ventricular pacing burden >40%.^139^, ^148^ More recently, CSP has been shown to reduce the composite outcomes (heart failure hospitalization, upgrade to CRT, and all‐cause mortality) by 47% in patients with ventricular pacing burden >20%.^167^ In an observational study of LBBAP, LBBAP also reduced the same composite outcomes by 54% compared with RVP.^168^.

Accordingly, CSP may be considered for patients with an indication for permanent pacing with normal left ventricular function in whom substantial ventricular pacing (>20%) is anticipated to avoid pacing‐induced cardiomyopathy. However, it should be noted that there are complications in performing CSP, including conduction system injury, worsening of tricuspid regurgitation, delayed interventricular septal perforation, and thromboembolism.^152^, ^153^, ^169^ Moreover, the clinical outcome in the long‐term follow‐up is not yet fully explored. Therefore, CSP is not recommended for patients in whom an increased ventricular pacing is not anticipated.

#### 7.4.2 Patients With Bradycardia and Mild‐to‐Moderate Left Ventricular Dysfunction (LVEF 35–50%)

The Biventricular Versus Right Ventricular Pacing in Heart Failure Patients with Atrioventricular Block (BLOCK HF) trial^137^ evaluated the efficacy of CRT in patients who had indications for pacing and heart failure with reduced LV function (LVEF ≤50%). The results demonstrated that patients randomly assigned to CRT had a lower incidence of the primary endpoints (all‐cause death, worsening heart failure requiring intravenous therapy, or an increase in the left ventricular end‐systolic volume index of ≥15%) compared with RVP. On the other hand, observational studies and meta‐analyses have shown that CSP has no adverse effect on LVEF and significantly improves LVEF.^131^, ^170^ In small randomized controlled trials (RCTs) and observational studies enrolling patients with atrioventricular block and mild‐to‐moderate left ventricular dysfunction, CSP significantly improved LVEF compared with RVP.^171^, ^172^ Moreover, a meta‐analysis assessing patients who upgraded from RVP either to CRT or CSP revealed that CSP significantly increased LVEF compared with RVP, and the extent of LVEF improvement was comparable to that of CRT.^173^ The advantages of selecting CSP over CRT are that CSP with only 2 transvenous leads may reduce the incidence of device infection and venous occlusion, and extend the longevity of the device.^174^, ^175^ Therefore, CSP should be considered for patients with an indication for permanent pacing and mild‐to‐moderate left ventricular dysfunction (LVEF 35–50%) in whom substantial ventricular pacing (>20%) is anticipated.

### 7.5 Patients With Conduction System Disturbance and Heart Failure (CRT Indication)

Both observational studies and randomized crossover trials have shown that HBP significantly improves intraventricular conduction and hemodynamic parameters, resulting in greater LVEF improvement and reverse remodeling of the left ventricle compared with CRT.^157^, ^176^, ^177^, ^178^, ^179^, ^180^ Regarding LBBAP, its procedural success rate in CRT‐indicated patients has been relatively high (82–97%).^181^, ^182^, ^183^ A previous study conducting a within‐patient comparison of HBP, LBBAP and CRT demonstrated that greater improvements in left ventricular electrical dyssynchrony and acute hemodynamic response were seen with HBP and LBBAP compared with CRT, with no difference between HBP and LBBAP.^146^ In addition, multiple observational studies, small randomized crossover trials and meta‐analyses in CRT‐indicated patients showed that LBBAP resulted in shorter paced QRS duration, more remarkable improvement in LVEF, and significant reduction in all‐cause death and heart failure hospitalizations compared with CRT.^184^, ^185^, ^186^, ^187^, ^188^, ^189^, ^190^, ^191^.

In an observational study comparing CSP as a first‐line therapy with CRT in CRT‐indicated patients, CSP significantly reduced heart failure hospitalizations compared with CRT, but the procedural success rate of CSP was significantly lower (84.4% vs. 94.7%).^192^ The potential role of CSP for CRT‐indicated patients had been reported, but there have been no large RCTs comparing CSP with CRT. Moreover, most of the clinical trials evaluating the efficacy of CSP for CRT have been conducted in patients with LBBB or those requiring substantial right ventricular pacing, and the efficacy of CSP for patients with non‐LBBB has not been fully elucidated.^147^, ^193^.

Importantly, CRT demonstrated prognostic improvement in heart failure patients with wide QRS in several RCTs.^136^, ^194^, ^195^ Therefore, CRT is an established treatment and the current first‐line therapy for CRT‐indicated patients (especially for LBBB patients).^5^ However, several factors limit the benefit of CRT: an absence of optimal coronary veins for implantation of a left ventricular lead, failure of left ventricular lead placement due to technical issues, difficulty in continuing CRT due to phrenic nerve stimulation or high pacing threshold, and an insufficient response to CRT (CRT non‐responders).

Sharma et al. evaluated the feasibility of HBP as an alternative therapy for CRT in patients with failed left ventricular lead placement or non‐response to CRT.^170^ Their study presented a high procedural success rate for HBP, shortened paced QRS duration, and improvements in LVEF and heart failure symptoms. LBBAP showed similar results in a multicenter observational study.^196^ Therefore, in CRT‐indicated patients with LBBB or substantial ventricular pacing, CSP should be considered when CRT is ineffective or cannot be established for any reason. CSP combined with left ventricular lead (His‐optimized CRT: HOT‐CRT, LBB‐optimized CRT: LOT‐CRT) has been attempted in patients in whom CRT or CSP alone failed to improve electrical dyssynchrony.^197^, ^198^ This technique has been shown to achieve better resynchronization and is expected to be a novel therapeutic option for CRT non‐responders, and a biventricular pacemaker (CRT‐P) device should be selected for performing CSP. When a CRT‐D device is considered, a specific configuration of leads and ports^142^ is required (not covered by Japan's National Health Insurance as of February 2024).

### 7.6 Patients Requiring Atrioventricular Junction Ablation

In several RCTs, CRT has preserved LVEF after atrioventricular junction ablation compared with RVP.^199^, ^200^ CSP also demonstrated similar effects in a RCT.^201^ In addition, CSP significantly improved LVEF compared with CRT in heart failure patients with chronic atrial fibrillation and LVEF ≤40%.^202^.

Therefore, CSP may be considered in patients requiring atrioventricular junction ablation. When device implantation precedes atrioventricular junction ablation, an LBBAP lead may reduce lead‐related adverse events in the acute and chronic phases and is associated with a higher success rate in creating atrioventricular block compared with HBP.^203^.

## 8 CRT FOR MID‐RANGE QRS

CRT has been shown in multiple RCTs to be effective in patients with moderate to severe heart failure with reduced LVEF despite optimal medical therapy and a QRS duration ≥120 ms.^204^, ^205^, ^206^, ^207^, ^208^, ^209^ In these RCTs and meta‐analyses, complete left bundle branch block (CLBBB) waveform, and wide QRS (>150 ms) predicted the benefit of CRT,^204^, ^205^, ^206^, ^207^, ^208^, ^209^, ^210^, ^211^ and mid‐range QRS duration between 120 and 150 ms (120 ms≤QRS duration<150 ms) showed insufficient benefit of CRT, so‐called “nonresponders”.^210^, ^211^ On the other hand, clinical characteristics for higher CRT efficacy have been proposed, such as sex, body size (including racial differences), and heart size, and if these are taken into account, CRT may be effectively used for mid‐range QRS cases.^133^, ^194^, ^212^, ^213^, ^214^, ^215^, ^216^.

However, there is no consensus on the interpretation of these clinical characteristics, and there are currently differences in the definitions of mid‐range QRS and recommended classes of CRT in various societies’ guidelines.^92^, ^145^, ^217^, ^218^ (**Table** **8**). In preparing this Focus Update, we reviewed the recommended classifications based on the results of studies reported since the JCS/JHRS 2019 Guidelines on the Nonpharmacotherapy of Cardiac Arrhythmias.

**TABLE 8:** Recommendations for CRT Implantation for Mid‐Range QRS in Each Society's Guidelines (for Sinus Rhythm).

Guideline	COR	LOE	QRS morphology	LVEF (%)	QRS duration (ms)	NYHA functional class	Other
2023 HRS/APHRS/LAHRS^145^	I	A	LBBB	≤35	120–149	II–IV	Female, etc.
	IIa	B‐R	LBBB	≤35	120–149	II–IV	
	IIb	B‐NR	Non‐LBBB	≤35	120–149	III–IV	
	III	B‐R	Non‐LBBB	≤35	120–149	I–II	
2022 AHA/ACC/HFSA^217^	IIa	B‐NR	LBBB	≤35	120–149	II–IV	
	IIb	B‐NR	Non‐LBBB	≤35	120–149	III–IV	
2021 ESC^92^	IIa	B	LBBB	≤35	130–149		
	IIb	B	Non‐LBBB	≤35	130–149		
2017 CCS^218^	I	A	LBBB	<35	130–149	II–IV	
	III	–	LBBB	<35	<120–129	II–IV	

Level of Evidence A: substantiated by multiple RCTs or meta‐analyses; B: substantiated by a single RCT or large clinical trial that is not a randomized intervention; B‐R: moderate‐quality evidence from ≥1 RCT; B‐NR: moderate‐quality evidence from ≥1 well‐designed and conducted non‐RCT, observational trial, or case–control study. ACC, American College of Cardiology; AHA, American Heart Association; APHRS, Asian Pacific Heart Rhythm Society; CCS, Canadian Cardiovascular Society; COR, Class of Recommendation; ESC, European Society of Cardiology; HFSA, Heart Failure Society of America; HRS, Heart Rhythm Society; LAHRS, Latin American Heart Rhythm Society; LBBB, left bundle branch block; LOE, Level of Evidence; LVEF, left ventricular ejection fraction; NYHA, New York Heart Association; RCT, randomized controlled trial.

### 8.1 Lower Limit of QRS Duration for CRT Indication

The lower limit of mid‐range QRS in heart failure patients for whom CRT should be recommended has been controversial. Yu et al. reported that among patients with LVEF ≤35% and narrow QRS, there were cases of dyssynchrony on echocardiography (tissue Doppler), suggesting that these patients may be responders for CRT.^219^, ^220^ However, a subsequent multicenter prospective study (PROSPECT) reported that echocardiographic dyssynchrony is unlikely to predict CRT responders at high rates.^221^.

In 2013, the results of EchoCRT, an RCT of patients with LVEF ≤35%, QRS duration ≤130 ms, and dyssynchrony on echocardiography, were presented.^222^ In the study, a CRT device was implanted in all patients, and 2 groups, CRT‐on and CRT‐off, were compared. There was no significant difference in the incidence of the primary endpoint (heart failure hospitalization and death) between groups (HR 1.2, CI 0.92–1.57, P=0.15), and the mortality rate was significantly higher in the CRT‐on group (HR 1.81, CI 1.11, P=0.02). Based on these results, the efficacy of CRT in patients with QRS duration <130 ms was judged to be low, and the ESC and CCS guidelines recommend Class III.^92^, ^218^.

On the other hand, after the publication of EchoCRT, some reports were published suggesting the efficacy of CRT even in patients with 120 ms≤QRS duration<130 ms. De Pooter et al. examined the incidence of septal flush, which is considered the best echocardiographic predictor of CRT responsiveness in LBBB patients, and reported that more than 60% of women with mid‐range QRS had this finding.^223^ Furthermore, a subanalysis of EchoCRT showed the usefulness of CRT for patients with small left ventricular end‐diastolic volume,^213^ suggesting that sex, body size, and left ventricular size are useful in the process of selecting CRT.

Several clinical trials have reported that the response to CRT may be different in Japan, where many heart failure patients are smaller in stature than those in Europe and the USA. Oka et al. retrospectively analyzed the event rate (composite of all‐cause death or heart failure hospitalization) and responder rate by echocardiographic measures.^224^ Patients with LBBB and QRS duration ≥150 ms had the lowest event rate (28.9%) and highest responder rate (74%). In comparison, they also reported that although responder rates were significantly lower in patients with 120 ms≤QRS duration<150 ms than those with QRS duration ≥150 ms, good responses were observed in more than half (51% of LBBB and 52% of non‐LBBB patients) of the cases.

Varma et al. analyzed CRT in CLBBB cases and showed that QRS duration modified by left ventricular volume defined by echocardiography correlated with the efficacy of CRT in women.^212^ Other studies have reported that QRS duration modified by left ventricular end‐diastolic volume was significantly associated with prognosis in CRT patients, especially in women with small body size.^215^ A meta‐analysis of 5 RCTs also suggested that sex, QRS duration, etiology of heart failure, left ventricular end‐diastolic diameter and body height influenced all‐cause mortality and first hospitalization rates for heart failure.^206^.

Varma et al. also analyzed data from Advanced CRT, a registry of 251 Asian CRT cases (27% of the registry), including Japanese patients, and reported that the CRT responder rate, as defined by symptom score, was significantly higher in Asians than in non‐Asians in both the 120 ms≤QRS duration<150 ms and QRS duration ≥150 ms groups.^216^ Furthermore, when cardiac death or heart failure events were used as the endpoints, the benefit of CRT was demonstrated in Asians with 120 ms≤QRS duration<150 ms, and QRS duration modified by height was strongly associated with CRT efficacy.

The lower limit of QRS duration for recommending CRT is still controversial. However, considering that many RCTs have demonstrated the efficacy of CRT by including patients with QRS duration ≥120 ms, and that mid‐range QRS patients can be expected to be responsive in Japan, where there are many patients with small body size, the lower limit of QRS duration in this Focus Update has been set at 120 ms. However, it should be noted that there is strong evidence for the efficacy of CRT at QRS duration ≥130 ms.^225^.

### 8.2 Recommendations

Recommendations for CRT indications in mid‐range QRS in sinus rhythm are shown in **Table** **9** and a list of conditions is shown in **Table** **10**.

**TABLE 9:** Recommendations and Levels of Evidence for CRT Implantation for Mid‐Range QRS (for Sinus Rhythm).

	COR	LOE
CRT is recommended in HF patients with LVEF ≤35%, NYHA functional class II–IV, 120 ms≤QRS duration <150 ms and LBBB and female sex	I	A
CRT should be considered in HF patients with LVEF ≤35%, NYHA functional class II–IV, 120 ms≤QRS duration<150 ms and LBBB	IIa	B
CRT may be considered in HF patients with LVEF ≤35%, NYHA functional class III–IV, 120 ms≤QRS duration <150 ms and non‐LBBB	IIb	B
CRT may be considered in HF patients with LVEF ≤30%, NYHA functional class II, HF, 120 ms≤QRS duration<150 ms and non‐LBBB	IIb	B

COR, Class of Recommendation; CRT, cardiac resynchronization therapy; HF, heart failure; LBBB, left bundle branch block; LOE, Level of Evidence; LVEF, left ventricular ejection fraction; NYHA, New York Heart Association.

**TABLE 10:** Recommendations for CRT Implantation for Mid‐Range QRS (in Sinus Rhythm).

COR	LOE	QRS morphology	LVEF (%)	QRS duration (ms)	NYHA functional class	Other
I	A	LBBB	≤35	120–149	II–IV	Female, etc.
IIa	B‐R	LBBB	≤35	120–149	II–IV	
IIb	B‐NR	Non‐LBBB	≤35	120–149	III–IV	
IIb	B‐R	Non‐LBBB	≤30	120–149	II	

Level of Evidence A: substantiated by multiple RCTs or meta‐analyses; B‐R: moderate‐quality evidence from ≥1 RCT; B‐NR: moderate‐quality evidence from ≥1 well‐designed and conducted non‐RCT, observational trial, or case–control study. COR, Class of Recommendation; CRT, cardiac resynchronization therapy; LBBB, left bundle branch block; LOE, Level of Evidence; LVEF, left ventricular ejection fraction; NYHA, New York Heart Association.

#### 8.2.1 Mid‐Range QRS and LBBB

As noted earlier, it has been suggested that sex differences affect the efficacy of CRT, and to analyze this effect, a meta‐analysis using RAFT, MADIT‐CRT, and REVERSE, which showed a benefit of CRT on heart failure hospitalization or death, stratified LBBB patients with a QRS duration of 120–180 ms in 10 ms increments.^224^ The results showed no sex difference in the 120 ms≤QRS duration <130 ms group, but the benefit was significantly higher in both the 130 ms≤QRS duration<140 ms group and the 140 ms≤QRS duration<150 ms group only in women (relative risk reduction 85% and 69%, respectively). However, in the QRS duration ≥150 ms group, CRT significantly reduced the incidence of both heart failure and death, as well as death alone, without a sex difference, suggesting that a potential mechanism for sex differences in response to CRT may be related to anatomic differences, particularly body height (with a greater effect seen with shorter height).^212^, ^215^, ^226^, ^227^, ^228^, ^229^, ^230^, ^231^, ^232^, ^233^.

Based on these results, CRT implantation in heart failure patients with NYHA functional class II or higher, with mid‐range QRS of 120 ms≤QRS duration<150 ms and LBBB, is recommended as Class I for women and Class IIa for men (**Table** **9**).

#### 8.2.2 Mid‐Range QRS and Non‐LBBB

Clinical studies demonstrating the efficacy of CRT with mid‐range QRS in non‐LBBB patients are still limited. In an observational study of 99 heart failure patients (LVEF <35% and NYHA functional class II or higher) with non‐LBBB (right bundle branch block in 22.2% and intraventricular conduction disturbance in 77.8%) and QRS duration ≥120 ms, CRT improved LVEF by 4% over a 13‐month observation period.^234^ Subsequently, subanalyses of non‐LBBB patients were conducted in 2 large RCTs (MADIT‐CRT, RAFT) that enrolled a large number of patients with NYHA cardiac function class II,^135^, ^136^ and neither showed a benefit of CRT.

Based on these results, the recommended class of CRT for mid‐range QRS (120 ms≤QRS duration<150 ms) in non‐LBBB is unchanged from the JCS/JHRS 2019 Guidelines on the Nonpharmacotherapy of Cardiac Arrhythmias, with LVEF ≤35% in NYHA cardiac function class III or higher and LVEF ≤30% as a condition for Recommendation Class IIb.

However, the efficacy of CRT for patients with QRS duration <120 ms or <130 ms varies among reports,^92^, ^145^, ^217^, ^218^ so the indication for CRT should be carefully considered in each case.

## 9 CATHETER ABLATION

## 1 CATHETER ABLATION PROCEDURES FOR ATRIAL FIBRILLATION IN ADDITION TO PULMONARY VEIN ISOLATION (TABLE 11)

**TABLE 11:** Recommendations and Levels of Evidence for AF Ablation Procedures in Addition to PVI.

	COR	LOE
For initial ablation of persistent AF, left atrial posterior wall isolation may be considered in addition to PVI	IIb	B
In persistent AF with LVA in the left atrium, ablation of LVA may be considered in addition to PVI	IIb	B
VOM ethanol infusion may be considered for atrial tachycardia in which the VOM is part of a circuit that makes it difficult to ablate the arrhythmia by other methods of catheter ablation	IIb	C
In catheter ablation for long‐standing persistent AF, the addition of VOM ethanol injection to conventional PVI may be considered	IIb	B

AF, atrial fibrillation; COR, Class of Recommendation; LOE, Level of Evidence; LVA, low‐voltage areas; PVI, pulmonary vein isolation; VOM, vein of Marshall.

In catheter ablation of atrial fibrillation (AF), pulmonary vein isolation (PVI) alone is not effective in maintaining sinus rhythm in some cases, especially in patients with persistent AF. In addition to PVI, various techniques for ablation of non‐pulmonary veins substrates (beyond PVI) have been proposed, and many randomized controlled trials (RCTs) have investigated the efficacy of beyond PVI in maintaining sinus rhythm. This Focus Update offers a comprehensive review of these updates.

### 1.1 Additional Ablation to PVI for Non‐PV Substrates

The STAR‐AF2 study, which focused on catheter ablation for persistent AF, evaluated the effectiveness of additional intervention to PVI such as left atrial linear ablation (including the left atrial roof line and mitral isthmus line) or ablation of complex fractionated atrial electrograms (CFAEs) during AF.^235^ Contrary to widespread expectations, the study did not confirm the benefits of additional ablation. Similarly, the concurrent CHASE‐AF trial also yielded comparable results,^236^ casting doubt on the value of incorporating extra ablation methods in addition to PVI in the treatment of persistent AF.

On the other hand, the EARNEST‐PVI trial, which aimed to establish the non‐inferiority of PVI alone vs. PVI plus additional ablation (including linear ablation or CFAE ablation) in cases of persistent AF, did not demonstrate non‐inferiority of PVI alone,^237^ which was evident in the higher tendency of recurrence in the group receiving PVI alone.

The main causes of recurrence following AF catheter ablation include reconnection of the isolated PVs and the persistence of non‐PV arrhythmic substrates. During the time of the STAR‐AF2 and CHASE‐AF trials, reconnection of the PVs was frequently observed and was considered the primary cause of recurrence. However, by the time of the EARNEST‐PVI trial, the quality of PVI had improved, which might have made the effects of additional ablation more apparent. In recent years, there has been widespread adoption of ablation techniques including contact force and stability at the tip of the ablation catheter, and cryoballoon ablation. Consequently, there has been an increase in cases of no reconduction of PV potentials upon recurrence.^238^ The mechanism of recurrence in these cases is thought to involve non‐PV arrhythmic substrates, which may indicate that the effects of additional ablation have become more pronounced. This shift highlights a crucial aspect in evaluating the efficacy of catheter ablation: the impact of technological advancements in ablation catheters and related equipment on the outcomes of additional ablation of non‐PV substrates. The evolution of these technologies significantly influences the effectiveness of additional ablation strategies, underscoring the importance of considering technological progress in the assessment of treatment efficacy.

Furthermore, a post‐hoc analysis of the EARNEST‐PVI trial investigated the characteristics of the patient groups for whom additional ablation was effective vs. those for whom it was not. In that study, patients were stratified using the DR‐FLASH score, a predictor of left atrial low‐voltage areas (LVA) (diabetes, renal impairment, female sex, left atrial enlargement, age, hypertension, persistent AF). Additional ablation proved effective in patients with a higher likelihood of arrhythmic substrates.^239^ That finding underscored the importance of careful consideration in clinical practice regarding how to approach patient selection, which types of additional ablation techniques to apply, and which ablation technologies to use.

### 1.2 Left Atrial Posterior Wall Isolation

Posterior wall isolation is a popular additional ablation in catheter ablation for AF and involves augmenting PVI with a left atrial roof line and a line along the bottom of the left posterior wall. However, recent RCTs examining the effects of left atrial posterior wall isolation have yielded inconsistent results.

The CAPLA trial, for instance, evaluated the efficacy of adding left atrial posterior wall isolation to PVI in the initial catheter ablation of persistent AF.^240^ The study observed a tendency for increased recurrence of atrial tachycardia. In the group that underwent posterior wall isolation, reconnection of the conduction block created by additional linear ablation may have formed circuits for iatrogenic atrial tachycardia, causing recurrence and negating the therapeutic benefit. Similarly, the RILI trial explored the effectiveness of posterior wall isolation in conjunction with PV re‐isolation in patients experiencing PV reconnection during repeat catheter ablation for AF.^241^ That study also reported suboptimal results that raised the possibility that the impact of PV reconnection may have obscured any additional benefits derived from posterior wall isolation.

On the other hand, some trials have demonstrated the efficacy of left atrial posterior wall isolation. There are reports that the additional posterior wall isolation improved outcomes in patients with persistent AF who did not have low voltage area in the left atrium and in whom atrial arrhythmias were induced by continuous stimulation.^242^ There are also reports that the addition of posterior wall isolation to PVI improved outcomes in cryoballoon ablation for persistent AF were enhanced by adding posterior wall isolation to PVI,^243^, ^244^ although we should note that in Japan, as of February 2024, insurance does not cover cryoballoon ablation for left atrial posterior wall isolation. A meta‐analysis of the effects of additional left atrial posterior wall isolation to PVI indicated that posterior wall isolation might not be effective for paroxysmal AF, but could be beneficial for persistent AF.^245^.

Thus, routine implementation of posterior wall isolation is not yet substantiated by sufficient evidence from RCTs, but selectively applying this technique to certain patients might prove effective. It is also important to consider potential complications associated with left atrial posterior wall isolation, such as esophageal‐related complications, including left atrial–esophageal fistula,^246^ highlighting the need for careful patient selection.

### 1.3 Low Voltage Area Ablation

Left atrial LVA have attracted attention as indicators of myocardial damage that can serve as a substrate for arrhythmias. Several recent RCTs have explored ablation of LVA, but the results have been inconsistent, with some studies reporting limited additional benefits and others showing effectiveness.^247^, ^248^, ^249^, ^250^, ^251^, ^252^.

The ERASE‐AF trial showed that adding LVA ablation to PVI reduced atrial arrhythmia recurrence from 50% to 35% at 12 months in persistent AF, improving outcomes beyond PVI alone (P=0.006).^251^ In the STABLE‐SR‐III trial, an RCT conducted among patients aged >65 years with paroxysmal AF found that the addition of LVA ablation significantly reduced recurrence rates, particularly in patients with LVA ablation (hazard ratio [HR] 0.49, 95% confidence interval [CI] 0.25–0.94, P=0.03).^252^ Meta‐analyses have indicated that although LVA ablation does not show significant benefits in paroxysmal AF, it is associated with a notably higher non‐recurrence rate in persistent AF.^253^.

These trials examining the efficacy of LVA ablation showed variability in the definition of the LVA (low‐potential cutoff value or multipoint mapping electrode catheter used), ablation endpoint (homogenization of the LVA or completion of linear ablation such as posterior wall isolation or anterior wall line), and patient background (proportion of patients with LVA). Establishing a standardized method for ablation of LVA is an important issue. Furthermore, the presence of LVA may reflect overall atrial myocardial fibrosis, and in such cases further progression of the arrhythmic substrate post‐ablation can be anticipated,^254^ which raises concerns about the long‐term preventive effects against recurrence, even if short‐term efficacy is achieved. Given these considerations, despite recent meta‐analyses and multiple RCTs gradually building evidence for the effectiveness of LVA ablation in persistent AF, it is not yet considered a fully established method. Considering the potential adverse effects on left atrial function due to extensive ablation and the possibility of creating substrates for atrial tachycardia, this Focus Update recommends LVA ablation as a Class IIb indication in cases of persistent AF with LVA.

### 1.4 Chemical Ablation of the Vein of Marshall

The vein of Marshall (VOM), a remnant of the embryonic left superior vena cava, is susceptible to sympathetic and parasympathetic influences and has been implicated in the initiation and maintenance of AF.^255^ Additionally, the VOM itself forms part of the arrhythmic circuit, causing difficulty in block formation during linear ablation of the mitral annulus and atrial tachycardia that is difficult to ablate from the endocardial side (VOM‐related atrial tachycardia).

Retrograde balloon cannulation and ethanol injection into the VOM (VOM‐EI) creates a chemical ablation lesion in the area vascularized by the VOM. VOM‐EI ablates the myocardium of the VOM, eliminating AT circuits, AF triggers, and parasympathetic innervation in this region. Additionally, it induces endocardial injury to the myocardium surrounding the mitral annulus, which the VOM vascularizes. It has been proposed that the VOM‐EI may contribute to the treatment of AF.^256^.

The VENUS trial, which focused on catheter ablation for long‐standing persistent AF, found that adding VOMEI to PVI significantly improved recurrence‐free rates compared with conventional treatment (49% vs. 38%, P=0.04).^257^ In a meta‐analysis that included this trial, the VOM‐EI group had significantly better outcomes.^258^ VOM‐EI is effective in cases of atrial tachycardia in which the VOM is part of the circuit and in cases of refractory long‐standing persistent AF, and VOM‐EI for these patients is considered reasonable.

However, this approach requires unique technical skills and experience, and controlling the extent of tissue damage from ethanol injection is difficult, posing potential risks. Additionally, as of February 2024, there is a lack of specialized equipment for this purpose, and existing medical devices need to be used off‐label, which is not yet approved for insurance coverage. Therefore, while promising, several issues still need to be addressed in the application of VOM‐EI.

### 1.5 Other Additional Ablation Strategy

Various methods and devices for identifying additional non‐PV ablation sites are being proposed, including focal impulse and rotor modulation (FIRM) mapping for rotor ablation, CardioInsight^TM^, and ExTRaMap^TM^. However, since the publication of the 2021 JCS/JHRS Guideline Focus Update Edition on Arrhythmia Nonpharmacological Treatment, there has been limited evidence for these techniques.

As new ablation methods, there are approaches such as targeting fractionated signal areas in the atrial muscle (FAAM) during sinus rhythm, which are considered the source of non‐PV triggers,^259^ and techniques focusing on spatiotemporal electrogram dispersion as drivers of AF.^260^ However, there is currently insufficient evidence to validate the effectiveness of these methods. Additionally, the BELIEF trial reported the effectiveness of left atrial appendage isolation adding to PVI.^261^ Furthermore, the isolation of the left atrium in cases of extensive LVA has also been proposed.^262^ However, these isolation techniques are challenging to perform, and there is an increased risk of thrombosis post‐isolation. Given the limited evidence supporting these additional ablation techniques, the risk of adverse outcomes, and the level of expertise required, their implementation should be carefully considered.

## 2 EXPANDED INDICATION FOR ATRIAL FIBRILLATION CATHETER ABLATION

### 2.1 Atrial Fibrillation Catheter Ablation as First‐Line Treatment

Catheter ablation as first‐line treatment for paroxysmal atrial fibrillation is considered a recommended Class IIa in the Arrhythmia nonpharmacologic treatment guidelines (revised 2019).^5^ Recently, 3 RCTs investigated the efficacy of cryoballoon ablation as first‐line treatment for paroxysmal AF^263^, ^264^, ^265^ (**Table** **12**).

**TABLE 12:** Results of RCTs Showing the Effectiveness of Cryoballoon Ablation as First‐Line Treatment.

Trial name	Country in which the law is being enforced	No. of registered patients (persons)	Target AF type (%)	Mean follow‐up (months)	Arrhythmia detection method	**Recurrence rate (%)** *
STOP‐AF First^263^	USA	203	Paroxysmal 100	12	• 12‐lead ECG. • ECG telemonitoring (weekly). • Holter ECG (after 6 and 12 months).	25.4/55.0
EARLY‐AF^264^	Canada	303	Paroxysmal 95	12	Implantable loop recorder	42.9/67.8
Cryo‐FIRST^265^	Australia, Europe, South America	218	Paroxysmal 100	12	• Ambulatory ECG. • 7‐day Holter ECG.	17.8/32.4

* Ablation/medication. AF, atrial fibrillation; ECG, electrocardiogram; RCT, randomized controlled trial.

The STOP AF First trial^263^ compared cryoballoon PVI with antiarrhythmic agents (groups I or III) in 203 patients with symptomatic paroxysmal AF at 24 centers in the USA. After 12 months of follow‐up, treatment success rates (successful procedure, non‐recurrence of atrial arrhythmia, etc.) were significantly higher in the ablation group (74.6% vs. 45.0%, P<0.001). Only 2 patients in the ablation group had procedure‐related complications (pericardial effusion and myocardial infarction), but the authors concluded that serious complications were rare.

The Early‐AF trial^264^ compared cryoballoon PVI with antiarrhythmic drug rhythm control in 303 patients with untreated and symptomatic paroxysmal AF at 18 Canadian centers. All patients underwent arrhythmia detection with an implantable ECG and were followed for 12 months. Recurrence of atrial tachyarrhythmia (AF, atrial flutter, atrial tachycardia) was significantly lower in the ablation group (42.9 vs. 67.8%, P<0.001). Serious complications occurred in 5 patients in the ablation group (3.2%, 3 patients with transverse paralysis, 2 patients with symptomatic bradycardia) and 6 patients in the antiarrhythmic drug group (4.0%, 2 patients with wide‐QRS tachycardia, 1 patient with syncope, 1 patient with worsening heart failure, 2 patients with symptomatic bradycardia), but there was no significant difference between the 2 groups.

The Cryo‐FIRST trial^265^ compared cryoballoon ablation with antiarrhythmic drug therapy in 218 patients with untreated or symptomatic paroxysmal AF. The 12‐month follow‐up showed a significantly lower rate of recurrent atrial tachyarrhythmias in the ablation group (17.8% vs. 32.4%, P=0.01), but no significant difference in the incidence of serious complications between the 2 groups.

A meta‐analysis of these 3 RCTs has also been reported.^266^ When initial treatment for AF was compared between cryoballoon ablation and medical therapy, ablation was associated with significantly fewer recurrent atrial tachyarrhythmias, better improvement in symptoms and quality of life (QOL), and reduced medical resource utilization (hospitalization) compared with medical therapy. Serious side effects were similar between groups.

These results indicate that cryoballoon ablation is superior to medical therapy as the first‐line treatment for symptomatic and recurrent paroxysmal AF. In this Focus Update, cryoballoon ablation is recommended as the first‐line treatment for symptomatic recurrent paroxysmal AF (**Table** **13**). It should be noted that all 3 RCTs were conducted in experienced centers known as “high‐volume centers,” and the long‐term efficacy of cryoballoon ablation is unknown because of the short‐term results (1 year).

**TABLE 13:** Recommendation and Level of Evidence for Cryoballoon Ablation for Symptomatic Paroxysmal Recurrent AF.

	COR	LOE
Catheter ablation with cryoballoon is recommended as first‐line treatment for symptomatic recurrent paroxysmal AF (selected after patient requests ablation and decision should be made after providing a thorough explanation of other options and the risks associated with the treatment)	I	A

AF, atrial fibrillation; COR, Class of Recommendation; LOE, Level of Evidence.

### 2.2 Indications for Catheter Ablation of Asymptomatic Atrial Fibrillation

Because AF is not immediately life‐threatening, catheter ablation has been performed to improve patients’ QOL by maintaining sinus rhythm. In other words, the indication for catheter ablation of AF is symptomatic AF, and European and American guidelines do not describe the indication for asymptomatic AF.

In the Guidelines for the Nonpharmacologic Treatment of Arrhythmias (2019 revision), only asymptomatic paroxysmal AF with recurrent episodes is considered a recommended Class IIb.^5^ The indications for catheter ablation in clinical practice are expanding beyond symptomatic AF, and new evidence reported in recent years is presented.

#### 2.2.1 Impact of Early Rhythm Control

The EAST‐AFNET trial^267^ is the first RCT to report the impact of early rhythm control treatment on outcomes in patients with AF. Patients with AF within 1 year of diagnosis were randomized to early rhythm control (antiarrhythmic drug therapy or catheter ablation) or conventional therapy (rate control). After a mean follow‐up of 5.1 years, study was terminated early because the primary endpoint (cardiovascular death, stroke, heart failure, or hospitalization for worsening acute coronary syndrome) was significantly higher in the conventional therapy group (3.9/100 patient‐years vs. 5.0/100 patient‐years, P=0.005).

Although the ablation rate in that study was relatively low (19.4% in the early rhythm control group and 7.0% in the conventional therapy group), it is meaningful that it was the first study to demonstrate that early rhythm control is associated with improved prognosis in patients with AF. Subsequent subanalysis additionally reported similar results in asymptomatic and symptomatic patients.^268^ An additional analysis showed that the prognostic benefit of early rhythm control was only observed in the high‐embolic risk group with a CHA_2_DS_2_‐VASc score ≥4 points.^269^.

#### 2.2.2 Comparison of Symptomatic and Asymptomatic Patients

The CODE‐AF trial^270^ is a prospective multicenter observational study in Korea in which 1,515 patients with AF (64% paroxysmal) were divided into 2 groups (symptomatic and asymptomatic), and their prognoses (primary endpoint: heart failure hospitalization, stroke, and cardiac death) were compared. Results showed that the symptomatic AF group had a poorer prognosis than the asymptomatic AF group (P=0.04), and rhythm control had a significantly lower incidence of primary endpoints than rate control, regardless of the presence or absence of symptoms. In the asymptomatic group, paroxysmal AF, left atrial diameter <50 mm, and CHA_2_DS_2_‐VASc score ≥3 were associated with improved prognosis.

#### 2.2.3 Catheter Ablation to Reduce Progression of Atrial Fibrillation

The ATTEST trial^271^ investigated whether radiofrequency catheter ablation could prevent progression from paroxysmal to persistent AF compared with antiarrhythmic drug therapy. A total of 255 patients were randomized 1 : 1 and followed for 3 years. Results showed that progression to persistent AF (or atrial tachycardia) occurred in 2.4% of the ablation group, compared with 17.5% in the antiarrhythmic drug treatment group, which demonstrated a reduction in AF progression by catheter ablation.

A subanalysis of EARLY‐AF^272^ reported that progression to persistent AF was significantly reduced in patients treated with cryoballoon ablation as first‐line therapy for paroxysmal AF compared with those treated with antiarrhythmic drugs (1.9% in the ablation group vs. 7.4% in antiarrhythmic drug group. HR 0.25, 95% CI 0.09–0.70). In addition, the study showed a significant improvement in QOL in the ablation group and a 69% lower rate of hospitalization compared with the antiarrhythmic drug group.^272^.

These 2 RCTs are significant because they demonstrate for the first time that catheter ablation inhibits the progression of AF and is not performed only for symptomatic improvement.

#### 2.2.4 Improving Patient Outcomes With Catheter Ablation

As described in the 2021 JCS/JHRS Guideline Focus Update for Nonpharmacologic Treatment of Arrhythmias,^6^ CABANA, a large RCT comparing whether AF catheter ablation improves patient outcomes compared with medical therapy did not clearly demonstrate an advantage of catheter ablation. Intention‐to‐treat analysis of the primary endpoint showed no significant difference between the groups, but per‐protocol analysis showed a significant improvement in the ablation group compared with medical therapy (P=0.046).^273^.

#### 2.2.5 Summary

Although there are no published RCTs that clearly demonstrate that catheter ablation improves the prognosis of patients with asymptomatic AF, new evidence is accumulating that (1) early SR maintenance therapy is associated with prognosis in patients with AF, and (2) catheter ablation prevents the progression of AF.

Considering that the purpose of AF catheter ablation is not only to improve patient symptoms and QOL, but also to meet the reality and demand in the field, this Focus Update is based on the above evidence, and recommends to consider catheter ablation for asymptomatic paroxysmal recurrent AF with a CHA_2_DS_2_‐VASc score 3 as Class of IIa (**Table** **14**).

**TABLE 14:** Recommendation and Level of Evidence for Catheter Ablation for Patients With Asymptomatic Paroxysmal Recurrent AF.

	COR	LOE
Catheter ablation should be considered for patients with asymptomatic recurrent paroxysmal AF and CHA_2_DS_2_‐VASc score ≥3 points	IIa	B

AF, atrial fibrillation; COR, Class of Recommendation; LOE, Level of Evidence.

### 2.3 Catheter Ablation for Atrial Fibrillation in the Patients With Heart Failure

Recently, a meta‐analysis of RCTs has shown the efficacy of catheter ablation in patients with heart failure (HF) complicated by AF.^274^, ^275^ Most of the RCTs included patients with HF with low left ventricular ejection function (HFrEF), in which catheter ablation reduced the all‐cause mortality rate and improved the LVEF, 6‐minute walk, and QOL compared with medical therapy. However, the study designs, including patient population, ablation methods and follow‐up duration, were not uniform among the RCTs, so the results should be interpreted with extra caution^276^, ^277^, ^278^, ^279^, ^280^, ^281^, ^282^, ^283^ (**Table** **15**).

**TABLE 15:** Recent RCTs on the Treatment of AF With HF (Ablation Therapy vs. Medical Therapy).

Trial name (year)	No of patients	Age (years)	AF phenotype	NYHA	LAD (mm)	LVEF (%)	Follow‐up (months)	Primary endpoint (vs. medical therapy)	Other results (vs. medical therapy)
RAFT‐AF^276^ (2022)	411	67	PAF/PsAF	II–III	46	30	37	Reduction of all‐cause mortality/HF, HR 0.71 (P=0.066)	Improvement in LVEF and QOL, decrease of NT‐proBNP
AMICA^277^ (2019)	202	65	PsAF	II–III	50	26	12	No significant improvement in LVEF (P=0.36)	Higher SR maintenance rate, reduction of AF/AT burden, no significant improvement in 6MWD, QOL or BNP
CABANA^278^ (2019)	778	68	PAF/PsAF	II–IV	–	55	49	Reduction of death/stroke/hemorrhage/cardiac arrest, HR 0.64 (95% CI 0.41–0.99)	Reduction in mortality rate, improvement in QOL
CASTLE‐AF^279^ (2018)	363	64	PAF/PsAF	II–IV	48	32	37.6	Reduction of all‐cause mortality/HF, HR 0.62 (P=0.007)	Improvement in LVEF/no improvement in 6MWD
CAMERA‐MRI^280^ (2017)	68	61	PsAF	II–IV	48	33	6	Improvement in LVEF (P<0.0001)	Decrease of LVESV, LA volume and BNP, improvement in NYHA, 6MWD was improved but not significantly
AATAC^281^ (2016)	203	61	PsAF	II–III	47	30	24	Higher SR maintenance rate (P<0.0001)	Reduction of hospitalization and death, improvement in LVEF, 6MWD and QOL
CAMTAF^282^ (2014)	50	58	PsAF	II–III	51	33	6	Improvement in LVEF (P<0.001)	Reduction of LVESD, improvement in V˙O_2max_ and QOL, decrease of BNP
ARC‐HF^283^ (2013)	52	63	PsAF	II–III	48	24	12	Increase of peak V˙O_2_ (P=0.018)	Decrease of BNP, improvement in QOL, V˙O_2max_ and 6MWD, LVEF improved but not significantly

6MWD, 6‐minute walk distance; AF, atrial fibrillation; BNP, B‐type natriuretic peptide; COR, Class of Recommendation; HF, heart failure; LAD, left atrial dimension; LOE, Level of Evidence; LVEF, left ventricular ejection fraction; LVESD, left ventricular end‐systolic dimension; LVESV, left ventricular end‐systolic volume; NT‐proBNP, N‐terminal pro‐BNP; QOL, quality of life; RCT, randomized controlled trial; SR, sinus rhythm; V˙O_2max_, maximum oxygen consumption.

The RAFT‐AF^276^ trial is the largest RCT in recent years, and showed a trend towards reduced all‐cause mortality and HF hospitalization rates in the ablation therapy group compared with the medical therapy (rate control) group (P=0.066). Although the difference was not statistically significant, most of the events were seen after 18 months of enrollment, suggesting that the observation period may have been inadequate. The relatively large trials of CASTLE‐AF,^279^ and AATAC,^281^ and the CABANA subanalysis^278^ demonstrated a significant reduction in deaths in the ablation therapy group compared with the medical therapy group, suggesting that prognostic efficacy of catheter ablation in patients with HFrEF is high.

In terms of sufficient observation period, the CABANA trial with 49 months of follow‐up is noteworthy, and in a subanalysis focusing on patients with HF (35%),^278^ ablation therapy significantly reduced the primary composite endpoint of death, severe stroke, major bleeding and cardiac arrest compared with medical therapy (HR 0.64, 95% CI 0.41–0.99). In addition, catheter ablation therapy significantly reduced both AF recurrence (56% vs. 72%, HR 0.56, 95% CI 0.42–0.74) and AF burden, and also improved QOL.^278^ It should be noted, however, that the study included patients with mild HF (76% of patients were NYHA II and median LVEF was 55%). Long‐term results (7.8 years of follow‐up) of the combined population of the CAMTAF^282^ and ARC‐AF^283^ trials were also recently reported. There was no significant difference in death or cardiovascular hospitalization between the ablation therapy group and the medical therapy group, but 54% of the medical therapy group had undergone ablation at the end of the study. Treatment‐based prognostic analysis showed that the ablation therapy group had significantly lower mortality (HR 0.43, 95% CI 0.20–0.91, P=0.028) and mortality/cardiovascular hospitalization (HR 0.48, 95% CI 0.24–0.94, P=0.031) rates compared with the drug treatment group.^284^.

The AMICA trial assessed improvement in cardiac function (left ventricular ejection fraction: LVEF) as a primary endpoint in patients with persistent AF and LVEF <35%, and the trial found that the improvement in LVEF was similarly observed in both groups, although the rate of sinus rhythm (SR) maintenance at 1 year was significantly higher in the ablation therapy group (73.5% vs. 50%, P=0.001).^277^ These conflicting results may be due to shorter follow‐up duration (1 year) and more severe population compared with other trials. In fact, the AMICA trial included patients with severe low LVEF (26%) and severe HF (60% of patients with NYHA III, and 43% of patients with CRT‐D implantation). The ARC‐HF trial also did not show a significant difference in LVEF improvement, but patients with a markedly low LVEF of 24% were included.^283^.

The CAMERA‐MRI trial, an evaluation of cardiac function by magnetic resonance imaging (MRI), showed significant improvement in LVEF in the ablation therapy group. In particular, the late gadolinium enhancement (LGE)‐negative group had a significantly greater improvement in LVEF compared with the LGE‐positive group (22.3% vs. 11.6%, P=0.0069) and LGE‐negative patients were more likely to normalize LVEF (EF≥50%) (73% vs. 29%, P=0.0093).^280^ The results were similarly observed after 4 years’ follow‐up.^285^ A subanalysis of the same study investigated the effect of catheter ablation on cardiac function in patients with persistent AF and idiopathic low LVEF, and found that myocardial T1 time, a surrogate of diffuse fibrosis, was significantly decreased and LVEF had significantly improved in the ablation therapy group compared with the medical therapy group.^286^ That result indicated that catheter ablation therapy is an effective treatment for AF‐induced cardiomyopathy. Based on these results, catheter ablation is strongly recommended for patients with AF‐induced cardiomyopathy caused by cardiac dysfunction secondary to tachycardia or irregular and asynchronous myocardial contractions, or cardiac dysfunction that recovers by SR restoration, because catheter ablation can be highly expected to restore cardiac dysfunction in such patients.^286^, ^287^, ^288^, ^289^.

On the other hand, the CASTLE‐AF trial could not show a significant benefit of catheter ablation therapy over medical therapy in patients with NYHA III or LVEF <25%.^279^ In light of the results of the AMICA trial^277^ and ARC‐HF trials,^283^ it should be noted that both the cause and severity of LV dysfunction, as well as the severity of HF, may affect the clinical outcome.

In August 2023, results from the CASTLE‐HTx trial, which assessed the efficacy of catheter ablation therapy with medical therapy in patients with symptomatic AF and endstage HF who were referred for heart transplantation evaluation, were published.^290^ The study showed a significant reduction in the composite primary endpoint (all‐cause death, heart transplantation, left ventricular assist device implantation) in the combination of catheter ablation and medical therapy group compared with the medical therapy alone group during 18 months’ follow‐up (8% vs. 30%, HR 0.24, 95% CI 0.11–0.52, P<0.001). This result was supported by a significant reduction in AF burden (reduction rate: 30.8%/year vs. 8.3%/year) and a significant improvement in cardiac function (LVEF improvement rate: 7.8%/year vs. 1.4%/year) in the combination of catheter ablation and medical therapy group. These results suggest that catheter ablation in combination with medical therapy is worthwhile for patients with AF and endstage HF if their condition is stable. However, the study did not demonstrate the efficacy of catheter ablation in patients with LVEF <25%.

In conclusion, catheter ablation therapy in patients with AF with HFrEF should be considered after careful consideration of the patient's background, and the etiology and severity of HF.

The efficacy of catheter ablation in patients with AF with HF with preserved EF (HFpEF) has been reported in many single‐center studies,^291^ and meta‐analyses showed that the rate of SR maintenance after catheter ablation therapy in patients with HFpEF was comparable to those without and those with HFrEF, and also demonstrated that catheter ablation therapy was associated with higher rate of SR maintenance, lower incidence of HF hospitalization and improvement in QOL compared with medical therapy.^292^, ^293^ Although a large‐scale RCT is still lacking, a post‐hoc analysis of the CABANA trial limited to patients with LVEF >50% found that catheter ablation therapy was associated with a 60% reduction in death compared with medical therapy (3.3% vs. 8.6%, HR 0.40, 95% CI 0.18–0.88).^278^ Furthermore, a recent RCT comparing the efficacy of catheter ablation (16 patients) and medical therapy (15 patients) in AF patients with HFpEF demonstrated that catheter ablation significantly improved hemodynamic parameters (pulmonary capillary wedge pressure [PCWP], cardiac output [CO]), exercise tolerance (peak V˙O_2_) and QOL at 6 months compared with medical therapy, although the study population was quite limited. In particular, HF was hemodynamically improved in 75% of AF ablation patients who successfully maintained SR, indicating the importance of maintaining SR in patients with HFpEF.^294^.

As noted, catheter ablation is a highly effective therapy in AF patients with HF; however, the indication for catheter ablation should be carefully judged based on the patient's background, including cardiac function, NYHA, underlying cardiac disease and AF duration. In particular, catheter ablation therapy may worsen the prognosis in patients with severe HF and advanced AF. In addition, complex procedures, older patients and multiple comorbidities (HF, renal dysfunction, hypertension, etc.) increase the risk of perioperative complications related to catheter ablation procedures, and therefore, careful handling of each case is recommended.^295^, ^296^.

Based on this evidence, we have made some changes in this Focus Update from the previous update regarding the recommendation of catheter ablation in AF patients with HF as follows. “Catheter ablation is recommended to reverse LV dysfunction in AF patients when AF induced cardiomyopathy is highly probable” (classified as Class I), and “Catheter ablation is considered in AF patients with HF with preserved LVEF (HFpEF) without comorbidities that contribute to HF to reduce mortality and HF hospitalization” (classified as Class IIb) (**Table** **16**).

**TABLE 16:** Recommendations and Levels of Evidence for Catheter Ablation of AF With HF.

	COR	LOE
Catheter ablation is recommended to reverse LV dysfunction in AF patients when AF induced cardiomyopathy is highly probable	I	C
Catheter ablation should be considered in selected patients with AF and HFrEF who are receiving guideline‐directed medical therapy for HF, to reduce mortality and hospitalization rates	IIa	A
Catheter ablation may be considered in patients with AF who have HF with HFpEF and no comorbidities contributing to HF, to reduce mortality and hospitalization rates	IIb	B

AF, atrial fibrillation; COR, Class of Recommendation; HF, heart failure; HFpEF, HF with preserved left ventricular ejection fraction; HFrEF, HF with reduced left ventricular ejection fraction; LOE, Level of Evidence; LV, left ventricular.

**CQ 1. Should Atrial Fibrillation Catheter Ablation Be Performed in Older Patients (>80 Years Old)?**

**Recommendation**

We recommend that the option of catheter ablation for symptomatic AF not be ruled out solely because of advanced age (defined in this Focus Update as 80 years).

Catheter ablation for asymptomatic AF in the very old, aimed at improving prognosis, is not recommended.

**Supplementary Item**

1. First, evaluate symptoms. Check for HF symptoms such as palpitations and shortness of breath, and whether there is a decrease in QOL or daily living activities due to the decline in cardiac function caused by AF.
2. There is considerable individual variation in the overall condition of the older patient (frailty, cognitive abilities, comorbidities). Evaluate the general condition in each case, considering the progression of AF (duration and left atrial remodeling), and decide on ablation treatment through shared decision‐making with the patient, weighing the benefits (symptom improvement) and risks.

**Background and Priority of This CQ**

Aging is a major risk factor for the development of AF, and its prevalence is high in older patients. Although AF catheter ablation is an invasive procedure and should be performed cautiously in older patients with low physical reserve and high comorbidity, the proportion of older patients undergoing real‐world AF catheter ablation is increasing significantly.

According to the registries conducted by the Japanese Heart Rhythm Society, the percentage of patients aged 75 years undergoing AF catheter ablation increased from 8.5% in 2011 (J‐CARAF Registry) to 28.3% in 2021 (J‐AB Registry).^297^ It is considered an important clinical issue to verify whether such a rapid spread of AF ablation in older patients is appropriate. However, because there are no RCTs specifically for this age group, evidence must be determined from registry studies and subanalyses of RCTs.

**Evidence Summary**

PICO

P : Older patients with AF

I : Catheter Ablation

C : Young patients with AF

O : Outcome

Significant outcomes related to benefit: recurrence‐free rate, QOL, prognosis.

Significant outcomes related to harm: complications associated with the procedure.

**Safety of Ablation in Older Patients With Atrial Fibrillation**

A meta‐analysis comparing the safety of catheter ablation in older and younger patients in registered studies has been conducted.^298^, ^299^, ^300^ In these studies, age was consistently an independent predictor of complications. A study using the Japanese DPC database also found that age was associated with complications, with even a significant difference in complication rates between those aged <60 years and those aged 60–64 years.^296^ The complication rate in the ≥85 age group (6.8%) was approximately 2.8‐fold higher than that in the <60 age group (2.5%). Considering that the overall complication rate was 5.8% in the 2011 J‐CARAF survey,^301^ the current rate in the group aged ≥85 years is not prohibitively high, but more cautious decision‐making is required compared with younger patients.

**Efficacy of Ablation in Older Patients With Atrial Fibrillation**

A meta‐analysis comparing recurrence rates after catheter ablation in older and younger patients in a registry study was inconsistent, with some studies reporting that age was associated with recurrence,^298^, ^299^ and others reporting no significant difference in recurrence rates between age groups.^300^ This result indicates that reasonable outcomes can be expected with appropriate patient selection.

**Quality of Life Improvement Effects of Ablation in Older Patients With Atrial Fibrillation**

Catheter ablation has been shown to significantly improve QOL for patients with symptomatic AF.^302^, ^303^ In an age‐specific subanalysis, the advantage of ablation over conservative treatment was consistent between older and younger patients, suggesting that catheter ablation may improve QOL in symptomatic AF regardless of age.^303^.

**Prognosis Improvement Effects of Ablation in Older Patients With Atrial Fibrillation**

Catheter ablation has not been conclusively proven to improve prognosis for the general patient population with AF.^273^ The CABANA trial, a RCT comparing catheter ablation with drug therapy for the treatment of AF with risk factors, found no significant difference between treatments. However, in a subanalysis by age, the catheter ablation group showed better prognosis in younger patients, but not in older patients, indicating a significant interaction.^304^.

The CASTLE‐AF trial, which showed that AF catheter ablation can significantly reduce all‐cause death and HF hospitalization in patients with HF, did not show such an effect in the older subgroup (≥65 years old).^279^ In older patients, factors other than AF may influence prognosis. Therefore, catheter ablation of asymptomatic AF for the primary purpose of improving prognosis is generally not recommended.

**Conclusion**

Although advanced age does present a significant risk for complications in AF ablation, the reported incidence of these complications is not excessively high. Furthermore, efficacy does not show a significant difference compared with younger patients. Therefore, age alone should not be the sole criterion for deeming ablation unsuitable. Although ablation's effect in maintaining SR can lead to improved QOL, its impact on overall prognosis remains unclear. Therefore, AF ablation in older patients should be considered for those who are presumed to be at low risk from the procedure based on their overall health status. The primary objective should be to improve QOL and daily living activities that have been diminished due to symptomatic AF.

### 2.4 Angioplasty for Pulmonary Vein Stenosis After Atrial Fibrillation Catheter Ablation

Pulmonary vein stenosis (PVS) is a well‐known complication of AF catheter ablation, although its incidence of PVS is recognized as relatively rare (3.4–42.4%), and 0.7–3.6% of patients require interventional treatment due to some symptoms.^305^, ^306^, ^307^, ^308^ The incidence has reported to be relatively high when segmental PV ostial isolation was performed for AF, but the incidence decreased once the wide‐area circumferential ablation technique became popular for PVI. However, with the spread of balloon technology, the incidence has increased again, and caution is needed. The wide variation in incidence is due to different methods for detecting PVS. Some reports assessed only symptomatic patients, while others prospectively evaluated all patients by computed tomography (CT) scan after catheter ablation.

Symptoms were variable, including cough, shortness of breath, dyspnea, chest pain, bloody sputum (hemoptysis), and recurrent pneumonia, and these symptoms usually occurred about 100 days after the procedure.^307^ In general, if stenosis is limited to a single PV, the patient is often asymptomatic, but if the stenosis involves multiple PVs or acutely progresses, some symptoms are more likely to occur.^308^ In particular, stenosis of the ipsilateral upper and lower PVs is likely to cause severe symptoms, and stenosis of 3–4 PVs may be life‐threatening.

**FIGURE 7:**
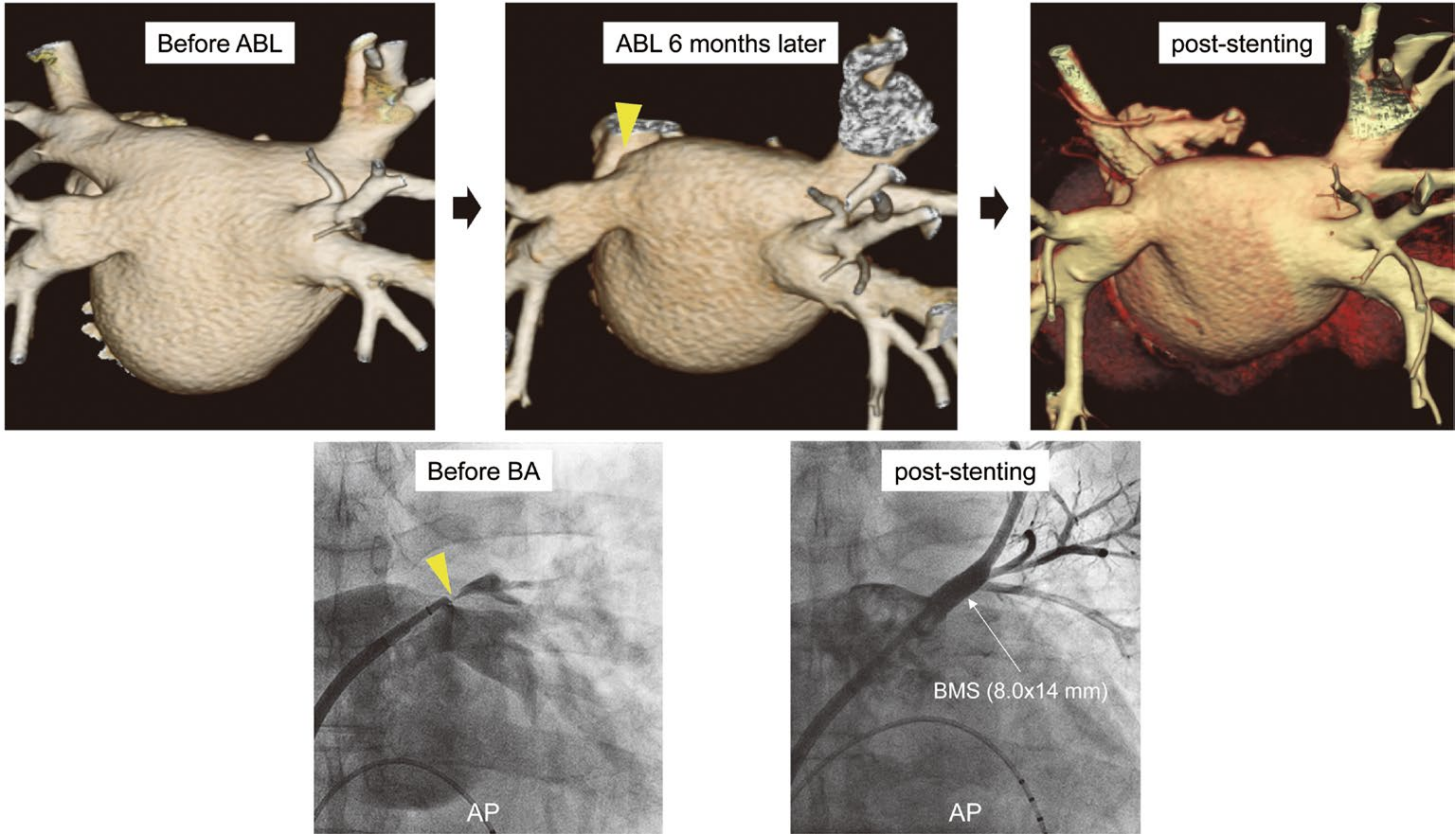
Percutaneous transluminal pulmonary vein angioplasty for severe pulmonary vein stenosis after catheter ablation for atrial fibrillation. Balloon dilatation and stenting were performed for severe stenosis of the left upper pulmonary vein. ABL, ablation; AP, anteroposterior view; BA, balloon angioplasty; BMS, bare metal stent.

There were no guidelines regarding treatment of PVS in the past. Percutaneous transluminal PV angioplasty has been reported as an effective treatment for PVS or PV occlusion in Japan^309^ and other countries.^310^ In Japan, percutaneous transluminal PV angioplasty has not been approved by insurance as of February 2024 (**Figure** **7**).

Because there are no devices specifically designed for the PVs (balloon and stent), percutaneous transluminal PV angioplasty is performed mainly using devices for the lower limb vessels, and its efficacy and safety have been reported at the single‐center level.^309^, ^310^, ^311^, ^312^ Complications associated with this procedure, including cardiac tamponade, stent loss, and cerebral infarction, are estimated to be 3–4%,^313^ but no complications have been reported in Japan.^309^ In addition to complications, restenosis after stent dilation should be considered with this procedure. The restenosis rate after stenting has been reported to be significantly lower than that of balloon dilation alone, so it is important to obtain a large dilation diameter.^311^, ^312^ There are only scattered reports of surgical intervention for PVS,^314^ and efficacy and safety are not clear.

The number of cases of PVS after AF ablation is small, and the efficacy and safety of this technique have not yet been established. In the clinical setting, it is currently performed out of necessity in cases of PVS or occlusion with symptoms or lung dysfunction. Therefore, we describe the current status of this treatment in this Focus Update. It is recommended that the indication of PV angioplasty must be thoroughly assessed, informed consent be given by the patient, and the procedure should be performed under the backup of cardiovascular surgery and the cooperation of an experienced arrhythmia specialist and a percutaneous coronary intervention specialist.

## 3 NEW ABLATION THERAPY FOR ATRIAL FIBRILLATION: PULSED FIELD ABLATION

### 3.1 Principles and Properties

When an electric field is externally applied to a cell by direct current with an extremely short pulse width (nanoseconds to microseconds), a force corresponding to the magnitude of the electric field intensity is applied to the cell membrane. When this force becomes larger than the level at which the cell membrane can maintain its structure, small pores are created in the lipid bilayer of the membrane. If the applied voltage is near a critical voltage (i.e., the voltage that provides an electric field strength just high enough to disrupt the cell membrane) for a very short time, the holes formed in the cell membrane are small and the membrane can be spontaneously repaired (reversible electroporation).

However, when an electric field far exceeding a critical voltage is applied, a large irreparable hole is created in the membrane, leading to cell death (irreversible electroporation). Pulsed field ablation (PFA) uses a catheter to induce irreversible electroporation in targeted myocardial cells, creating lesions.^315^.

The threshold of the electric field for irreversible electroporation of cardiomyocytes is much lower than that of vascular smooth muscle, endothelial cells, and nerves. Current thermal energy‐mediated catheter ablation procedures cause damage to surrounding tissues (pulmonary vein stenosis, phrenic nerve palsy, left atrial esophageal fistula, gastric dysmotility, etc.), but PFA selectively injures target myocardial cells, so the risk of adjacent tissue damage is extremely low.^315^.

PFA does not need to generate contact force between the catheter and the target, as is the case with radiofrequency catheter ablation, and the effect of PFA is not weakened by insufficient contact force. The durability of PVI may be maintained if the catheter and target site are not too far apart. Thus, PFA is expected to greatly improve both the efficacy and safety of current catheter ablation techniques.

### 3.2 Clinical Data

The first in‐human clinical trial of PFA (IMPULSE/PEFCAT) was performed in 81 patients with paroxysmal AF.^316^ The catheter was a 12Fr over‐the‐wire type (FARAWAVE^TM^) with a basket‐ or petal‐shaped tip that fitted the shape of the pulmonary vein entry. PFA delivery time for PVI was <3 min, and total procedure time was 1.5 h, including 3‐dimensional mapping of the left atrium (av. 18 min).

The output waveform was modified from monophasic to biphasic 1, 2, and 3 throughout the study period, and the maintenance of PVI at 3 months improved from 18% in the monophasic setting to 100% in the biphasic 3 setting. The safety profile of the study included only 1 patient with cardiac tamponade, and no other adverse events (e.g., stroke, phrenic nerve palsy, pulmonary vein stenosis, or esophageal injury). A total of 121 patients were followed up, and at 1 year, the SR maintenance rate was 78.5% for all patients and 84.5% for patients with optimized waveforms.^317^.

Clinical trials of PFA catheters for PVI were performed, using several other manufacturers’ catheters besides FARAWAVE^TM^, and in 2023 the results of the PULSED AF trial using a loop‐type PFA catheter (PulseSelect^TM^, Medtronic, Inc.) were published.^318^ That study was a multicenter, prospective, observational study of 300 patients with AF (150 paroxysmal, 150 persistent) from 41 centers in 9 countries, including Japan. The 1‐year follow‐up showed that the primary efficacy endpoint (rate of acute procedural failure/recurrent arrhythmia/avoidance of antiarrhythmic drug escalation) was significantly higher for paroxysmal than for persistent AF (66.2% and 55.1%, respectively), and the primary safety endpoint (procedure‐ or device‐related adverse events) was 0.7% (1 cerebrovascular event/150 paroxysmal AF patients, 1 cardiac tamponade/150 persistent AF patients).

In 2023, the 1‐year follow‐up results of the VARIPULSE® variable loop catheter (Biosense Webster, Inc.) for PVI (inspire study) were also reported.^319^ The VARIPULSE® catheter is integrated with the CARTO system, a 3‐dimensional mapping system, enabling both mapping and PFA with the same catheter. Of the 226 patients with drug‐refractory symptomatic paroxysmal AF in the study, 83 patients reached 1‐year follow‐up. The non‐recurrence rate of symptomatic AF, atrial flutter, and atrial tachycardia was 78.9%, and no major adverse events were observed during the period.

All 3 trials were prospective studies of a PFA group alone, but in August 2023 the results of a prospective RCT (ADVENT) comparing a PFA group with a radiofrequency/cryoablation group were reported, showing that PFA was noninferior in both efficacy and safety.^320^.

### 3.3 Safety

Left atrial esophageal fistula is a rare but fatal complication. The myocardial selectivity of PFA is a promising feature for avoiding this injury. In an experiment using swine esophagi ablated from the inferior vena cava, injury (including 1 left atrial esophageal fistula) occurred in all subjects in the radiofrequency ablation (RFA) group (4 cases), but not in the PFA group (6 cases).^321^ Animal studies have also shown that PFA has minimal effect on the phrenic nerve.^322^ A single 200‐J PFA was delivered from the right atrium of swine to the phrenic nerve, which was captured in 17/19 cases immediately after ablation and in all 19 cases 30 min later; 15 cases were followed up 3–13 weeks later and showed no problems with the phrenic nerve. Clinically, transient phrenic nerve palsy immediately after PFA has been reported,^323^ but is not considered as a long‐term problem.

The effect of PFA on pulmonary vein stenosis was also examined in an animal study.^324^ Ten pigs underwent PFA in one pulmonary vein and RFA in another pulmonary vein, and the pulmonary vein diameters were evaluated angiographically before and after PFA and 3 months later. PFA showed an 11% decrease in pulmonary vein diameter immediately after ablation, but a 19% increase at 3 months. In contrast, RFA showed a 23% decrease immediately and a 7% decrease at 3 months. Similar results were clinically demonstrated in a subanalysis of the IMPULSE and PEFCAT studies,^325^ suggesting that PFA has no or very little effect on pulmonary vein stenosis.

Coronary artery spasm is currently considered the main concerning effect of PFA on the surrounding organs. A case of coronary spasm induced in the left circumflex artery after PFA to the mitral isthmus was reported,^326^ and its effects on coronary spasm have since been comprehensively studied.^327^ No coronary spasm was observed after PVI (25 patients) or posterior wall isolation (5 patients), but severe right coronary artery stenosis was induced in all 5 patients after ablation to the cavo‐tricuspid isthmus (CTI) and they recovered on average 5.5 min after nitroglycerin administration. Subsequently, PFA was performed to the CTI in 15 patients after coronary (5 patients) or intravenous (10 patients) nitroglycerin injection, and moderate and mild coronary stenosis was induced in 1 and 2 patients, respectively, but severe stenosis was not induced. Thus, coronary spasm should be kept in mind when PFA is performed in the immediate vicinity of a coronary artery.

### 3.4 Outlook

PFA injures the myocardium in a few seconds by direct current application, resulting in a shorter treatment time compared with conventional RFA or cryoenergy, and with less injury to surrounding organs due to its selective targeting of myocardial cells. Clinical studies to date suggest that efficacy is comparable, at least in the short term. Although PFA is expected to become the first choice for catheter ablation instead of thermal energy in the future, the evidence is still limited, and its efficacy and safety should continue to be carefully evaluated.

## 4 ADVANCES IN VENTRICULAR PREMATURE CONTRACTION (PVC) / VENTRICULAR TACHYCARDIA (VT) ABLATION

### 4.1 Evaluation of Arrhythmic Substrate in PVC / VT Patients

In the presence of ventricular arrhythmias, it is important to evaluate for structural heart disease, including coronary artery disease. The 2022 ESC guidelines^40^ recommend 12‐lead ECG, echocardiography, coronary evaluation with CT or coronary angiography, and cardiac magnetic resonance imaging (MRI) to investigate structural heart disease as a Class IIa indication. In cases of scar‐related VT, contrast‐enhanced MRI is useful for determining the ablation strategy, and in cases of suspected idiopathic VT, MRI is also recommended for identifying potential structural heart disease.

### 4.2 Indication and Timing

#### 4.2.1 Catheter Ablation for VT Associated With Structural Heart Disease

RCTs such as the SMASH VT,^328^ VTACH,^329^ SMS,^330^ and VANISH^331^ trials were conducted to investigate the efficacy of catheter ablation for the prevention of VT recurrence in patients after myocardial infarction and implantable cardioverter defibrillator (ICD) implantation. These studies have shown that catheter ablation is effective in preventing recurrent VT, especially in patients with ischemic heart disease who are taking amiodarone. Many RCTs conducted after the publication of the 2019 JCS/JHRS guidelines on the non‐pharmacotherapy of cardiac arrhythmias support the strategy of performing catheter ablation as the first‐line treatment for the prevention of recurrent VT.^5^.

Recent advances and widespread use of 3‐dimensional mapping systems and high‐density mapping using multipolar catheters have facilitated the identification of ablation target sites,^332^ thereby increasing the procedural success rate. The SURVIVE‐VT trial compared catheter ablation with antiarrhythmic drugs as a first‐line treatment for the prevention of recurrent VT associated with myocardial infarction. After 2 years of follow‐up, the ablation group had an improvement in the composite endpoint consisting of cardiovascular death, appropriate therapy by ICD, unscheduled hospitalization for heart failure, and treatment‐related serious complications (28.2% in the ablation group vs. 46.6% in the antiarrhythmic drug group, hazard ratio [HR] 0.52, 95% confidence interval [CI] 0.30–0.90, P=0.021). This difference was mainly driven by a significantly high incidence of drug side effects and serious drug treatment‐related complications such as the occurrence of slow VT below the VT detection zone of the ICD in the antiarrhythmic drug group. There was no difference in the incidence of cardiovascular death between the 2 groups. As of February 2024, the VANISH2 (NCT02830360) is ongoing to evaluate the superiority of catheter ablation as a first‐line treatment for sustained VT.

The PAUSE‐SCD trial is a multicenter RCT including Asian countries that compared catheter ablation prior to ICD implantation with medical therapy in patients with structural heart disease and a history of monomorphic VT.^333^ Patients with dilated cardiomyopathy and arrhythmogenic right ventricular cardiomyopathy as well as those with ischemic heart disease were included in the study, which is different from previous RCTs that mainly included patients with ischemic heart disease. During a mean follow‐up of 31.3 months, the incidence of primary endpoints (recurrent VT, hospitalization for cardiovascular events, and death) was significantly lower in the ablation group (49.3% in the ablation group vs. 65.5% in the control group, HR 0.58, 95% CI 0.35–0.96, P=0.04). This difference was mainly due to the reduction in VT recurrence in the ablation group, and there was no significant difference in the incidence of hospitalization for cardiovascular events or death between the 2 groups.

PARTITA trial^334^ is a European multicenter RCT published at the same time as the PAUSE‐SCD trial. It compared catheter ablation with medical therapy in patients who experienced the first appropriate shock after ICD implantation. Catheter ablation reduced ICD therapies and improved the composite endpoint (death or hospitalization for worsening heart failure: 4% in the ablation group vs. 42% in the control group, HR 0.11, 95% CI 0.01–0.85, P=0.034).

The BERLIN VT trial^335^ is another European multicenter RCT that aims to evaluate the optimal timing of VT ablation in patients with myocardial infarction and a history of sustained VT. It compared a prophylactic ablation before ICD/CRT‐D implantation with an elective ablation after at least 3 appropriate ICD shocks following ICD/CRT‐D implantation. The incidence of sustained VT was lower in the prophylactic ablation group; however, there was an increase in the incidence of hospitalizations for worsening heart failure and no improvement in the prognosis (32.9% in the prophylactic ablation group vs. 27.7% in the standby ablation group, HR 1.09, 95% CI 0.62–1.92, P=0.77). The results of the study did not validate VT ablation prior to device implantation to prevent VT recurrence.

Taken together, these results suggest that an early catheter ablation strategy significantly reduces recurrent VT and ICD therapies, especially in patients with ischemic heart disease, compared with antiarrhythmic drug therapy. However, there are conflicting results regarding whether catheter ablation reduces mortality and hospitalization for cardiovascular events, and future studies will be needed. It should be mentioned that complication rates were relatively high, ranging from 2.8% to 8.7%, in these RCTs, which were mainly conducted at experienced centers.^5^, ^328^, ^329^, ^330^, ^331^, ^332^, ^333^, ^334^, ^335^ With consideration of patient risk, ablation should be performed only at experienced centers.

#### 4.2.2 Idiopathic PVC / VT

Catheter ablation for idiopathic PVC/VT originating from the right ventricular outflow tract or left bundle branch has a high success rate with a low complication rate, and RCTs have shown that catheter ablation is more effective than antiarrhythmic drug therapy for idiopathic PVC/VT of the right ventricular outflow tract origin.^336^, ^337^ The 2022 ESC guidelines recommend catheter ablation as the first‐line treatment for symptomatic PVC/VT originating from the right ventricular outflow tract or left ventricular bundle branch as a Class I indication.^40^ Catheter ablation or flecainide is recommended as a Class IIa treatment for symptomatic PVC/VT of other origin. The success rate of catheter ablation for PVC/VT from sites other than the ventricular outflow tract is slightly less than that of originating from the ventricular outflow tract.^336^ In this Focus Update, we follow the 2019 JCS/JHRS guideline on non‐pharmacotherapy of cardiac arrhythmias.^5^ Catheter ablation is recommended as a Class I indication in patients with frequent PVC/non‐sustained ventricular tachycardia (NSVT; ≥10% of the total number of beats) who have serious symptoms or severe ventricular dysfunction due to tachycardia and for whom antiarrhythmic drugs are ineffective, not tolerated or not the patient's preference. In patients with symptomatic idiopathic PVCs originating from the right or left ventricular outflow tract and for whom antiarrhythmic drugs are ineffective, not tolerated or not the patient's preference, catheter ablation should be considered (Class IIa Recommendation). In patients with symptomatic idiopathic PVCs originating from sites other than the ventricular outflow tract and for whom antiarrhythmic drugs are ineffective, not tolerated or not the patient's preference, catheter ablation may be considered (Class IIb Recommendation).

#### 4.2.3 Epicardial Ablation for Brugada Syndrome

The effectiveness of endocardial or epicardial ablation of PVCs triggering VF, and epicardial ablation of an abnormal arrhythmogenic substrate in the right ventricular outflow tract have been reported in patients with Brugada syndrome with recurrent ICD shocks.^338^, ^339^ Successful epicardial ablation has already been reported, and the long‐term results are excellent.^340^.

The BRAVO registry^341^ reports the results of epicardial catheter ablation in 159 patients with Brugada syndrome. During a follow‐up period of approximately 4 years, the VF‐free survival rate after a single procedure was 81%, and the final success rate after repeat procedures was 96%. The 5‐year VF‐free survival rate was 98% in patients who did not develop type 1 ECG after a drug provocation test. Therefore, in this Focus Update, catheter ablation of VF‐triggering PVCs and epicardial ablation of the abnormal potential area in the right ventricular outflow tract are recommended as a Class IIa indication in patients with recurrent appropriate ICD shocks refractory to drug therapy (**Table** **17**). Currently, 2 RCTs investigating the efficacy of epicardial ablation for Brugada syndrome are ongoing (NCT03294278, NCT02704416).

**TABLE 17:** Recommendation and Level of Evidence for Catheter Ablation in Brugada Syndrome.

	COR	LOE
Catheter ablation of triggering PVCs and/or RVOT epicardial substrate should be considered in patients with Brugada syndrome with recurrent appropriate ICD shocks that are refractory to drug therapy or when pharmacological treatment is contraindicated due to adverse effects	IIa	B

COR, Class of Recommendation; ICD, implantable cardioverter defibrillator; LOE, Level of Evidence; PVC, premature ventricular contraction; RVOT, right ventricular outflow tract.

Recent studies have shown that there are some cases in which abnormal potential areas exist in the epicardial right inferior wall and left lateral wall as well as in the right ventricular outflow tract.^340^, ^341^ In particular, this is often observed in patients with an early repolarization pattern in the inferolateral leads on 12‐lead ECG.^340^ It has not been fully elucidated whether abnormal potentials found outside the right ventricular outflow tract are involved in the development of VF and should be ablated.

**CQ 2. What Is the Optimal Treatment for Asymptomatic Idiopathic PVC Without Evidence of Left Ventricular Dysfunction?**

**Recommendation**

In patients with idiopathic PVC who have no symptoms and no evidence of left ventricular dysfunction, it is recommended to follow‐up first and consider treatment after the evaluation described below.

**Supplementary Item**

2 The incidence of PVCs (the PVC burden), the presence of NSVT, and the presence of structural heart disease (echocardiography, cardiac contrast‐enhanced MRI) should be evaluated.
3 Regular follow‐up of ECG and cardiac function is recommended. When symptoms (palpitations, loss of consciousness, etc.) appear or cardiac function deteriorates, catheter ablation should be considered after shared decision‐making with the patient.

**Background and Priority of This CQ**

PVCs are often not accompanied by symptoms and prognosis is good in the absence of structural heart disease (i.e., idiopathic PVCs).^342^ Catheter ablation of idiopathic PVCs is highly effective with a low complication rate. Although rare, there is a concern about PVC‐induced cardiomyopathy and induction of lethal ventricular arrhythmias. The effectiveness of catheter ablation has been reported in such cases.^343^, ^344^ At present, there is no method to accurately predict the occurrence of PVC‐induced cardiomyopathy or lethal ventricular arrhythmias. It is important to evaluate whether ablation is beneficial for patients with asymptomatic idiopathic PVCs.

**Evidence Summary**

PICO

P : idiopathic PVC

I : Catheter Ablation

C : Observation

O : Outcome

Significant outcomes related to benefit: reduction in sudden cardiac death, worsening of LVEF, and hospitalization for worsening heart failure.

Significant outcomes related to harm: procedure‐related complications (cardiac tamponade).

**Risk Factors for PVC‐Induced Cardiomyopathy**

It is known that patients with a high incidence of PVCs (high PVC burden) are at a high risk for developing PVC‐induced cardiomyopathy. Baman et al. reported that the risk of developing PVC‐induced cardiomyopathy increased when the PVC burden was ≥24% of the total daily heartbeats on 24‐hour Holter ECG in 174 patients with idiopathic PVCs. On the other hand, they reported that there were no patients with LVEF worsening when the PVC burden was <10%.^345^.

There are intra‐day and daily variations in the incidence of PVCs. Hsia et al. suggested that 24–48 h of ECG monitoring is not sufficient for accurate assessment of PVC burden, and that prolonged ECG monitoring over ≥1 week is important.^346^ It is desirable to establish a more accurate risk assessment based on the PVC burden using long‐term ECG monitoring.

**Catheter Ablation for PVC‐Induced Cardiomyopathy**

According to 1 meta‐analysis including patients with a mean preoperative PVC burden of 24%, success rates of catheter ablation ranged from 66% to 90%, and mean LVEF improvement was 7.7% (95% CI 6.1–9.4%).^347^ In another report, the complication rate of catheter ablation was 2.4%, and no procedure‐related deaths occurred.^336^.

**Prognosis of PVC‐Induced Cardiomyopathy**

Lee et al. prospectively investigated the prognosis of 100 untreated and asymptomatic patients with idiopathic PVCs.^348^ Among patients with a mean PVC burden of 18.4% at enrollment, PVCs spontaneously resolved in 44 patients (44%) during a mean follow‐up of 15.4 months. The 4 patients (4%) without spontaneous resolution of PVCs had LVEF worsening (LVEF <50%), and 1 (1%) had heart failure. Niwano et al. followed 239 Japanese patients with PVCs ≥1,000 beats/day originating from the right or left ventricular outflow tract for an average of 5.6 years. They found that 13 patients (5.4%) had a decrease in LVEF of ≥6%; however, none of them manifested heart failure symptoms.^349^ In that study, deterioration of cardiac function was mainly observed in patients with PVCs of ≥20,000 beats/day. Based on the results of these studies, LVEF worsening is rare during the follow‐up of a patient with idiopathic PVCs, and it is even more rare to have a manifestation of heart failure symptoms. Even in cases of PVC‐induced cardiomyopathy, cardiac function restores within 4–6 months in many cases if PVCs are suppressed by catheter ablation, and the overall prognosis is considered favorable.^343^, ^350^.

There is no clear consensus on the therapeutic intervention for asymptomatic idiopathic PVCs, and clinical evidence that supports catheter ablation as the first‐line treatment is lacking. Regular follow‐up of ECGs and cardiac function is recommended.

When there is a risk of developing PVC‐induced cardiomyopathy, such as high PVC burden, catheter ablation may be considered after evaluation of the patient's background, the estimated success rate of ablation, and the risks associated with the procedure. The 2022 ESC guidelines recommend regular follow‐up of cardiac function in patients with asymptomatic idiopathic PVCs (Class I Recommendation). Catheter ablation is indicated only when the PVC burden exceeds 20%.^40^.

### 4.3 New Ablation Techniques

#### 4.3.1 New Mapping Methods of Detecting Arrhythmic Substrates

In scar‐related VT, it is sometimes necessary to identify the ablation target sites during baseline rhythm when monomorphic VT cannot be induced or hemodynamically unstable VT is induced. Because areas with low voltage and delayed/isolated potentials are associated with critical isthmuses of the VT circuits, ablation targeting these areas has been performed.^351^, ^352^ However, this can damage areas unrelated to the VT circuit.

Recently, the usefulness of functional substrate identification has been reported to estimate the location of the VT isthmus from activation maps obtained by high‐density mapping using catheters with multipolar electrodes during SR and pacing.^353^, ^354^, ^355^ The VT isthmus is highly correlated with the area where electrical propagation is relatively slow compared with other areas in the ventricle. Aziz et al. reported that the area of dense isochronal crowding (area of conduction delay in the ventricle: deceleration zone) revealed by the isochronal late activation map (ILAM) method correlated with the VT circuit. Ablation at the deceleration zone was highly successful in eliminating VT.^354^ Changing the direction of the wavefront in the ventricle by pacing may reveal areas of abnormal potentials that were not apparent during SR. Pacing from multiple directions is useful for identifying abnormal arrhythmic substrates. It has been reported that a pacing protocol using extrastimuli unmasked abnormal potentials that were not apparent during SR and that the areas with conduction delay during extrastimuli were associated with VT isthmuses.^356^, ^357^, ^358^.

#### 4.3.2 Special Ablation Techniques

Recent studies using high‐density mapping of the endocardium and epicardium during VT have shown that human VT is infrequently restricted to a single myocardial surface, but rather is characterized by complex 3‐dimensional activation.^359^ If the VT origin exists in the midmyocardium, endo‐ and epicardial ablations may be ineffective. Ethanol infusion into the coronary artery branch has been performed as an alternative approach to conventional RFA in such patients. Recently, the effectiveness of ethanol infusion into the coronary venous branch has been reported (not covered by insurance in Japan). Valderrábano et al. performed ethanol infusion into the coronary vein in patients with VT refractory to conventional catheter ablation and the 1‐year VT‐free survival rate was 84%.^360^ Stereotactic radiotherapy is expected to be a noninvasive treatment for refractory VT and many clinical studies are ongoing.^361^, ^362^ However, there are reports of serious complications such as pericardioesophageal fistula in the late phase, and studies evaluating the safety of stereotactic radiotherapy will be needed.^363^ As of February 2024, this treatment has not been approved in Japan.

Other approaches to treatment of VT with deep myocardial origin include ablation using low ionic irrigation (half‐normal saline),^364^ and long‐duration ablation (non‐approved therapy). The effectiveness of prolonged ablation with 20–35 W for >2 min (≤5 min) with careful observation of impedance drop has been reported for ventricular arrhythmias originating from the left ventricular summit.^365^ Because these approaches can increase the risk of steam pop and serious complications such as cardiac tamponade, the indications should be considered in light of efficacy and safety.

## 5 ATRIAL FIBRILLATION PHARMACOTHERAPY AND COMPREHENSIVE MANAGEMENT

## 1 JAPANESE ORIGINAL STROKE RISK ASSESSMENT TOOL: HELT‐E_2_S_2_ SCORE

### 1.1 HELT‐E_2_S_2_ Score Development Background

The CHADS_2_ and CHA_2_DS_2_‐VASc scores have been conventionally used for simple stroke risk assessment in patients with atrial fibrillation (AF). However, an integrated analysis of 3 Japanese registries (J‐RHYTHM registry, Fushimi AF registry, and Shinken Database) raised questions about their applicability in Japan.^366^ The analysis revealed that age (≥75 years), hypertension, and previous stroke were significant independent risk factors.^366^.

Further analysis in the J‐RISK study,^367^, ^368^ which included 2 additional registries (Hokuriku‐Plus AF registry and Keio Interhospital Cardiovascular Study), confirmed similar risk factors (age 75–84 years, hypertension, and previous stroke) and identified additional risks: age ≥85 years, body mass index (BMI) <18.5 kg/m^2^, and persistent/permanent AF.^367^ Conversely, diabetes, heart failure and vascular disease, components of the CHADS_2_ and CHA_2_DS_2_‐VASc scores, were not identified as independent risk factors (**Table** **18**).^368^.

**TABLE 18:** **HELT‐E**_**2**_ **S**_**2**_ **Score**.

Acronym	Risk factor	Score
H	Hypertension	1
E	Elderly, age 75–84 years	1
L	Low BMI (<18.5 kg/m^2^)	1
T	Type of AF (persistent/permanent)	1
E_2_	Extreme elderly, age ≥85 years	2
S_2_	Previous stroke	2

AF, atrial fibrillation; BMI, body mass index. (Modified from Okumura K, et al., 2021.^368^).

### 1.2 HELT‐E_2_S_2_ Score

**FIGURE 8:**
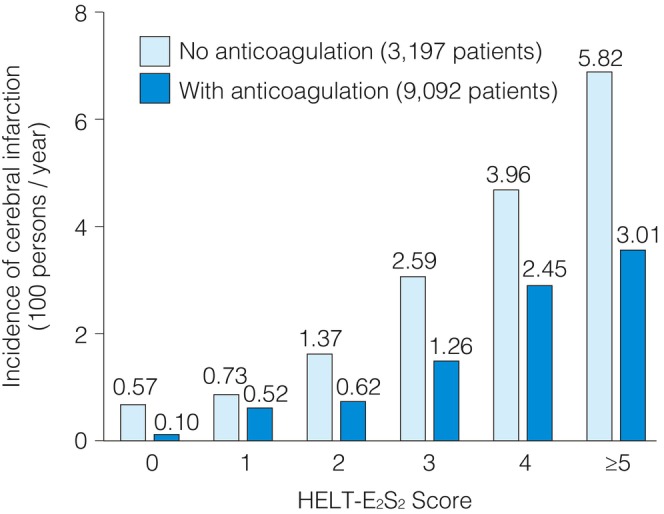
HELT‐E_2_S_2_ score and stroke incidence. (Adapted from Okumura K, et al, 2021.^368^)

**FIGURE 9:**
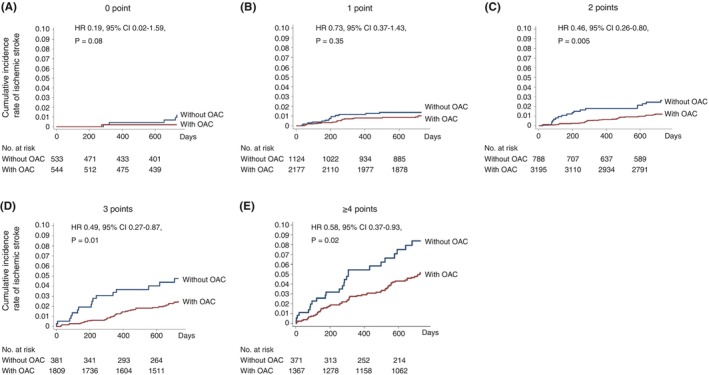
Stroke incidence by HELT‐E_2_S_2_ score in patients with and without anticoagulant (OAC). (Adapted from Okumura K, et al, 2021.^368^) CI, confidence interval; HR, hazard ratio.

In the J‐RISK study, 6 risk factors were weighted based on hazard ratios (HRs): 1 point for hypertension (H: Hypertension), age 75–84 years (E: Elderly), BMI <18.5 kg/m^2^ (L: Low BMI), persistent/permanent AF (T: Type of AF), and 2 points for age ≥85 years (E: Extreme elderly) and previous stroke (S: previous Stroke). Consequently, the HELT‐E_2_S_2_ score was developed, with a maximum of 7 points (**Table** **18**).^368^ The incidence of ischemic stroke, stratified by the HELT‐E_2_S_2_ score ranged from 0.57%/year for 0 points to 5.82%/year for ≥5 points in patients without anticoagulation therapy (**Figure** **8**).^368^ A significant reduction in the hazard of ischemic stroke was observed in patients with HELT‐E_2_S_2_ score ≥2 when comparing those with and without anticoagulation (**Figure** **9**).^368^ However, it is important to note that this difference in incidence does not necessarily reflect the efficacy of anticoagulant therapy, as the results were not adjusted for patient background (**Figures** **8**,**9**).^368^.

### 1.3 HELT‐E_2_S_2_ Score and Existing Risk Scores: Comparison and Consistency

In the J‐RISK study, the C‐statistic for the HELT‐E_2_S_2_ score in predicting incident stroke was 0.681, significantly higher than that of the CHADS_2_ score (0.647) and the CHA_2_DS_2_‐VASc score (0.641), with P values of 0.027 and 0.008, respectively.^368^ The C‐statistics in patients without/with anticoagulation were as follows: HELT‐E_2_S_2_ score 0.703/0.685, CHADS_2_ score 0.657/0.655 (comparison test for HELT‐E_2_S_2_ score, P=0.108/0.077) and CHA_2_DS_2_‐VASc score 0.655/0.646 (same comparison test, P=0.052/0.027).^368^.

An external validation using integrated data from the RAFFINE Study and SAKURA AF Registry in Japan found the C‐statistic for the HELT‐E_2_S_2_ score to be 0.661, slightly higher than the CHADS_2_ score (0.644) and CHA_2_DS_2_‐VASc score (0.650), though these differences were not statistically significant (P=0.15 and P=0.37, respectively).^369^ In the integrated analysis of these registries, all HELT‐E_2_S_2_ score components, except hypertension, were identified as independent risk factors.^369^.

Challenges remain in using the HELT‐E_2_S_2_ score for initiating anticoagulation therapy, which requires an analysis of the net clinical benefit, considering the balance between the risk of stroke and major bleeding. Currently, the JCS/JHRS 2020 Guideline on Pharmacotherapy of Cardiac Arrhythmias^4^ recommends starting anticoagulation therapy for patients with a CHADS_2_ score ≥1. The appropriateness of replacing this criterion with the HELT‐E_2_S_2_ score needs to be considered. The HELT‐E_2_S_2_ score includes 3 components of the CHADS_2_ score: age (≥75 years), hypertension, and previous stroke, but not diabetes or heart failure. Although diabetes and heart failure are not identified as independent risk factors, they do encompass high‐risk patients (see **Section** **1.3.3**). Therefore, the current guideline recommendation to initiate anticoagulation in patients with a CHADS_2_ score ≥1 remains valid. On the other hand, components of the HELT‐E_2_S_2_ score not included in the CHADS_2_ score, such as BMI <18.5 kg/m^2^ and persistent/permanent AF, are effectively considered under “other risks” in the JCS/JHRS 2020 Guideline on Pharmacotherapy of Cardiac Arrhythmias^3^ (now focusing on low body weight [≤50 kg] instead of BMI). Therefore, the components of the HELT‐E_2_S_2_ score align with the risk factors presented in the JCS/JHRS 2020 Guideline on Pharmacotherapy of Cardiac Arrhythmias.^3^ In this Focus Update, the flowchart for Recommendations for anticoagulation therapy in AF (**Figure** **12** in the 2020 Revision of the Guidelines for the pharmacological treatment of arrhythmias) has been left unchanged and further validation data should be accumulated in the future. The recommendations for each risk score in light of the HELT‐E_2_S_2_ score's emergence are shown in **Table** **19**.

**TABLE 19:** Recommendations and Levels of Evidence for Risk Assessment of Cardiogenic Embolism in Japanese Patients With AF.

	COR	LOE
Using the CHADS_2_ score is recommended	I	B
Using the HELT‐E_2_S_2_ score should be considered	IIa	B
Using the CHA_2_DS_2_‐VASc score may be considered	IIb	B

AF, atrial fibrillation; COR, Class of Recommendation; LOE, Level of Evidence.

#### 1.3.1 Hypertension

Hypertension is included in both the CHADS_2_ and CHA_2_DS_2_‐VASc scores, and was recognized as an independent risk factor for stroke in an integrated analysis of 3 Japanese registries.^366^.

In the J‐RISK study, patients with a baseline systolic blood pressure (SBP) ≥150 mmHg did not show a significantly different stroke risk than those with SBP <150 mmHg (HR 1.41, P=0.097).^370^ However, in the J‐RHYTHM registry, patients with a baseline SBP ≥136 mmHg (4th quartile) showed similar stroke risk to those with SBP <116 mmHg (1st quartile) (HR 1.01, P=0.968), but the risk was significantly increased when considering the SBP closest to the stroke event (HR 2.80, P<0.001).^371^ For diastolic blood pressure (DBP), patients in the 4th quartile (≥80 mmHg) were at increased risk compared with those in the 1st quartile (<65 mmHg) (HR 1.65, P=0.046).^371^.

Therefore, the risk of stroke in patients with hypertension is strongly influenced by blood pressure control throughout the disease course. The data from J‐RHYTHM registry suggests that maintaining SBP ≤136 mmHg and DBP <80 mmHg correlates with a lower risk of stroke.^371^ Conversely, poor control of either SBP or DBP is associated with a substantially higher risk of stroke. Moreover, inadequate blood pressure control is associated with an increased risk of cerebral hemorrhage, underscoring the importance of proper blood pressure control before initiating anticoagulation therapy.

#### 1.3.2 BMI <18.5 kg/m^2^ and Persistent / Permanent AF

The J‐RHYTHM registry reported a HR for embolism of 1.22 (95% CI 0.63–2.38) in patients with a BMI <18.5 kg/m^2^ compared with those with a BMI of 18.5–24.9 kg/m^2^, showing a trend towards increased risk, though not statistically significant.^372^ Meanwhile, the Fushimi AF registry identified a higher HR of 2.19 (P<0.01) for stroke or systemic embolism in patients weighing <50 kg.^373^ This suggests that low body weight or BMI may be a surrogate marker associated with conditions such as cancer and, or it could directly contribute to increased stroke risk through factors such enhanced neurohumoral activity and endothelial dysfunction.^373^.

It was traditionally believed that persistent/permanent AF posed a similar embolic risk as paroxysmal AF, based on subanalysis of large clinical trials.^374^ However, recent data suggest a higher stroke risk in persistent/permanent AF. For example, the ANAFIE registry reported an increased stroke risk in persistent/permanent AF compared with paroxysmal AF (HR 1.64/1.68, respectively, both P<0.001).^375^ Similarly, the Fushimi AF registry found the highest stroke risk when AF progressed from paroxysmal to persistent (HR 4.10, P<0.001 vs. paroxysmal AF that did not progress), and an elevated risk in already persistent/permanent AF (HR 2.20, P=0.025).^376^.

Persistent/permanent AF contributes to stroke risk through mechanisms such as progressive left atrial remodeling and endothelial damage (one of Virchow's triad). The duration of AF itself is associated with blood stagnation, another element of Virchow's triad, emphasizing the significant role of persistent/permanent AF in thrombus formation.

Reflecting on these insights, low body weight and persistent/permanent AF have been included as risk factors for considering anticoagulation therapy in the JCS/JHRS 2020 Guideline on Pharmacotherapy of Cardiac Arrhythmias.^3^.

#### 1.3.3 Diabetes Mellitus and Heart Failure

Diabetes and heart failure, included in the CHADS_2_ score, are not part of the HELT‐E_2_S_2_ score. However, it's important to note that both conditions encompass high‐risk patients for stroke, even if they are not identified as independent risk factors.

Studies examining stroke risk in AF patients with diabetes mellitus have shown varying results. In the ATRIA study, the stroke risk for AF patients with diabetes mellitus of <3 years duration was similar to those without diabetes.^377^ Another study found that in AF patients with diabetes, the ischemic stroke risk for those with diabetes duration of <5 years was comparable to that of patients with diabetes of 5–10 years’ (HR 1.20, P=0.12), but was significantly higher in patients with diabetes for ≥10 years (HR 1.45, P=0.001).^378^ Additionally, stroke risk in patients with HbA1c levels between 7% and 8% was similar to those with HbA1c between 6% and 7% (HR 1.08, P=0.47), increasing to HR 1.44 (P=0.004) in patients with HbA1c >8%.^378^.

In the ANAFIE registry, >90% of elderly Japanese AF patients were on anticoagulant drugs regardless of HbA1c level. The stroke/systemic embolism risk was comparable in patients with HbA1c between 7% and 8% to those with HbA1c <6% (HR 1.10) and slightly increased in patients with HbA1c ≥8% (HR 1.48, not statistically significant).^379^ The PREFER in AF study reported that diabetic patients on insulin therapy had a higher stroke risk (HR 2.19, P=0.009) than non‐diabetic patients, while those not treated with insulin had a similar risk to non‐diabetics (HR 0.93, P=0.80).^380^ Anticoagulation therapy should be considered for diabetic patients at a substantially high risk of ischemic stroke.

Regarding heart failure, the original CHADS_2_ score defines it as “recent heart failure”.^381^ European and American cohort studies report a 5–17‐fold increased stroke risk within 30 days following heart failure hospitalization.^382^, ^383^, ^384^ The Danish National Database Study found persistently elevated risk up to 30 years post‐hospitalization, remaining 1.5–2‐fold higher.^382^ Similarly, the Fushimi AF Registry observed the highest risk of stroke/systemic embolism within 30 days of heart failure hospitalization, with a sustained increase for up to 360 days post‐hospitalization (HR 3.94, 95% CI 2.42–6.17).^385^.

The phase III trial of direct oral anticoagulants (DOAC) defined heart failure as LVEF ≤40%, NYHA class ≥II, or recent heart failure symptoms within 3–6 months, a definition also used in the J‐RISK study.^367^ Although this definition is clear, patients with stable heart failure, adequately managed with modern heart failure medications, may not face a substantial stroke risk. However, a history of heart failure hospitalization is associated with a significant stroke risk.^385^.

In the Fushimi AF registry, elevated levels of B‐type natriuretic peptide (BNP) or NT‐proBNP (above median levels of ≥169.4 pg/mL or ≥1,457 pg/mL, respectively) were associated with an increased risk of stroke/systemic embolism (HR 1.97, P=0.03).^385^ Additionally, the Hokuriku‐Plus AF registry reported a higher thromboembolism risk, including ischemic stroke, in patients with BNP ≥170 pg/mL (vs. BNP <170 pg/mL; HR 3.86, P=0.0003).^386^.

**PQ 2. Which Patients Are Excluded From Anticoagulation Therapy?**

Although anticoagulation therapy has become widespread with the emergence of DOACs, certain patients are less likely to be eligible. For example, rivaroxaban, apixaban, and edoxaban are contraindicated in patients with a creatinine clearance (CCr) <15 mL/min, and dabigatran is contraindicated in patients with a CCr <30 mL/min. The Japanese Society for Dialysis Therapy (JSDT) generally advises against using warfarin in maintenance hemodialysis patients due to the increased risks of bleeding and embolism.^387^ This Focus Update follows the JSDT guidelines in contraindicating warfarin in maintenance hemodialysis patients.^387^ However, warfarin is necessarily used in certain scenarios, such as the perioperative period of AF ablation and for conditions such as mechanical valve replacements and secondary prevention of ischemic stroke.^3^.

Even if renal function is within the range of indications for anticoagulation, continuing anticoagulation can be challenging in patients with recurrent bleeding or a history of life‐threatening bleeding. Resuming anticoagulation after a bleeding episode may be possible if the bleeding source is identified and controlled. However, in cases of an unmanageable bleeding source, such as diverticular hemorrhage, continuing anticoagulation therapy becomes difficult. Cognitive, intellectual and psychiatric disabilities in patients, especially when lacking support for medication management, also pose a challenge.

The decision to discontinue anticoagulation based solely on advanced age is not universally agreed upon. A meta‐analysis, primarily from the warfarin era, revealed that anticoagulation does not significantly affect efficacy or bleeding in patients in their 80s and 90s.^388^ In contrast, recent data from the DOAC era suggest that anticoagulation offers a net clinical benefit for these age groups.^389^.

In Japan, a subanalysis of the ANAFIE registry, which enrolled nearly 30,000 older AF patients (including approximately 8,000 aged ≥85 years), reported varying anticoagulation rates by age. The study observed anticoagulation rates >90% up to age 90, 80% for ages 95–99, and 50% for age ≥100.^390^ Although severe renal dysfunction (CCr <30 mL/min) was common in these age groups (>20% for ages 85–89 and >40% for age ≥90), a significant proportion maintained CCr ≥30 mL/min. The decision not to anticoagulate should be based on individual characteristics such as renal function and bleeding risk, rather than age alone. In principle, anticoagulation should be administered as described in the package insert, even for older patients.

In practice, anticoagulation may be withheld based on the physician's judgment and patient preference. A cluster analysis of 2,445 ANAFIE registry patients not on anticoagulation identified 2 distinct groups.^391^ One was a low‐risk group (1,388 patients) with a mean age of 80.9 years, 100% paroxysmal AF, 21.0% previous catheter ablation, a 1.08% annual rate of stroke/systemic embolism, 0.69% major bleeding per year, and 2.72% annual all‐cause mortality rate. The other was a high‐risk group (1,057 patients) with a mean age of 84.9 years, a history of bleeding in 10.8%, a 3.30% annual rate of stroke/systemic embolism, 1.19% major bleeding per year, and 8.81% annual all‐cause mortality rate.^391^ This analysis should be interpreted carefully, as it may suggest the need for careful monitoring or the need for a broader application of anticoagulation therapy in potentially beneficial patients. Low‐risk patients should be monitored closely because stroke risk factors and AF burden may worsen with age. The high‐risk group had a higher incidence of ischemic stroke and a lower than expected incidence of major bleeding, indicating that more patients could be considered for anticoagulation under careful management.

The decision not to administer anticoagulation therapy to older AF patients should be made carefully, considering contraindications, bleeding risks, and patient preference. Physicians should make informed decisions after thorough explanations and understanding from both the patient and their family.

### 1.4 ANTICOAGULATION FOR HIGH‐RISK OLDER PATIENTS

Anticoagulation therapy with DOAC for prevention of stroke in patients with AF has steadily become routine daily practice in Japan since the first DOAC, dabigatran, was introduced in 2011.^391a^ At the same time, data on older patients with various risks associated with anticoagulation therapy have accumulated,^375,391b^ and here we summarize the latest evidence on how to treat older patients at high risk for anticoagulation therapy.

### 1.5 Renal Dysfunction

AF and chronic kidney dysfunction (CKD) frequently coexist: AF exacerbates CKD, and conversely, the progression of CKD increases the incidence of AF. Given that CKD poses a dual risk for both ischemic stroke and major bleeding, appropriate anticoagulation therapy is essential, especially in patients with CKD. However, because all DOACs are excreted by the kidneys (renal excretion rates are 80% for dabigatran, 50% for edoxaban, 35% for rivaroxaban, and 27% for apixaban), patients with a CCr <25 or 30 mL/min have been excluded from the large‐scale RCTs of DOACs and real‐world data of patients with severe CKD are also scarce.

In the J‐ELD AF registry of 3,015 Japanese AF patients aged ≥75 years taking on‐label doses of apixaban, 455 (15.1%) exhibited 15≤CCr<30 mL/min and most of these patients (97.4%) met the dose reduction criteria of apixaban.^392^ The annual incidence of stroke or systemic embolism in this cohort was 1.67%, which was comparable to the 1.76% in patients with CCr ≥50 mL/min (1,165 patients, 38.6%). The annual incidence of bleeding requiring hospitalization in patients with 15≤CCr<30 mL/min was 3.13%, which was numerically but nonsignificantly higher than the 1.39% in patients with CCr ≥50 mL/min (HR 2.00, P=0.075). Annual all‐cause and cardiovascular mortality rates were 7.87% and 2.62%, respectively, in patients with 15≤CCr<30 mL/min. These rates were significantly higher than the 1.75% in all‐cause mortality and 0.46% in cardiovascular mortality rates in those with CCr ≥50 mL/min.

In a subanalysis of the ANAFIE registry, among 26,202 Japanese patients aged ≥75 years with nonvalvular AF, the percentages of patients with CCr ≥50 mL/min, 30≤CCr <50 mL/min, 15≤CCr<30 mL/min, and CCr <15 mL/min were 44.2%, 41.1%, 13.2%, and 1.5%, respectively.^393^ The incidences of both stroke/systemic embolism and major bleeding increased with progression of CKD. The annual incidence of stroke or systemic embolism was 2.6% for CCr ≥50 mL/min and 4.0% for 15≤CCr<30 mL/min (HR 1.31; P=0.032). Further, the annual incidence of major bleeding was 1.8% for CCr ≥50 mL/min and 2.8% for 15≤CCr<30 (HR 1.12, P=0.439). In a comparison of DOAC and warfarin groups, the annual incidence of stroke or systemic embolism in patients with 30≤CCr<50 mL/min was 2.7% vs. 3.8% (HR 0.75, P=0.024), and that of major bleeding was 1.7% vs. 2.8% (HR 0.64, P=0.003). The incidences of both stroke/systemic embolism and major bleeding in the DOAC group were significantly lower than those in the warfarin group. The annual incidence of stroke/systemic embolism in patients with 15≤CCr<30 mL/min was 3.6% with DOAC vs. 4.0% with warfarin (HR 0.89, P=0.541), and the incidence of major bleeding was 2.4% with DOAC vs. 3.5% with warfarin (HR 0.67, P=0.065), and both stroke/systemic embolism and major bleeding were similar between the DOAC and warfarin groups. In a comparison of a non‐anticoagulant group and warfarin group, the annual incidence of stroke/systemic embolism in patients with 15≤CCr<30 mL/min was 5.9% vs. 4.0% (HR 1.80, P=0.047), which was significantly higher in the non‐anticoagulant group. On the other hand, the incidence of major bleeding was 2.3% in the nontreated group and 3.5% in the warfarin group (HR 0.65, P=0.306), which was not significantly different.

Patients with CKD are often elderly and frail. They also have high rates of comorbidities such as hypertension, diabetes mellitus, ischemic heart disease, and heart failure. Not only do these comorbidities contribute to impaired drug metabolism, but polypharmacy also increases the risk of drug–drug interactions and elevated anticoagulant blood concentrations, making it difficult to maintain the international normalized ratio (INR) within the optimal range in patients treated with warfarin. In a meta‐analysis of large‐scale RCTs of DOACs, treatment with a DOAC was associated with a lower risk of stroke/systemic embolism and major bleeding than warfarin in the 30≤CCr<50 mL/min group, consistent with the ANAFIE registry results.^394^ Although there are no RCTs comparing DOACs and warfarin in patients with CCr <30 mL/min, the results of the ANAFIE registry showed that a DOAC was at least as effective and safe as warfarin in patients with CCr <30 mL/min. Given that apixaban and edoxaban showed a lower risk of major bleeding at 30≤CCr<50 mL/min in a subanalysis of renal function in a large‐scale RCT^395^, ^396^ it seems reasonable to select these drugs in patients with 15≤CCr<30 mL/min.

Thus, real‐world data on DOACs in patients with moderate to severe renal dysfunction are accumulating. The JCS/JHRS 2020 Guideline on Pharmacotherapy of Cardiac Arrhythmias stated that DOACs are contraindicated in patients on dialysis, and so is warfarin except in some cases, such as the perioperative period of AF ablation, mechanical valves, and for secondary stroke prevention. Based on the evidence so far, this Focus Update has established anticoagulation recommendations for each stage of renal dysfunction (**Table** **20**). Because 16.4% of patients taking anticoagulants experience a decrease in CCr of ≥20% within 1 year,^397^ and the estimated glomerular filtration rate (eGFR) decreases over time by 1–2 mL/min/ 1.73 m^2^/year in patients with renal dysfunction,^398^ it is essential to perform regular blood tests once every “CCr value/10” months (e.g., once every 3 months for CCr 30 mL/min), as well as careful checking of liver function and hemoglobin level.

**TABLE 20:** Recommendations and Levels of Evidence for Treating Older Patients With AF at High Risk for Anticoagulation Therapy (Renal Dysfunction).

	COR	LOE
Anticoagulation for mild to moderate renal dysfunction with 30≤CCr<50 mL/min (DOAC preferred over warfarin) is recommended	I	A
Anticoagulation with DOAC (except for dabigatran) for severe renal dysfunction with 15≤CCr<30 mL/min should be considered	IIa	B
Anticoagulation with warfarin may be considered for endstage renal dysfunction with CCr <30 mL/min and nondialysis	IIb	C
Warfarin is not recommended for patients on dialysis*	III (No benefit)	B

* Except for perioperative AF ablation, mechanical valve, previous stroke. AF, atrial fibrillation; CCr, creatinine clearance; COR, Class of Recommendation; DOAC, direct oral anticoagulant; LOE, Level of Evidence.

### 1.6 Low Body Weight

Low body weight in patients with AF is a risk for developing stroke. In the HELT‐E_2_S_2_ score, BMI <18.5 (HR 1.55) was identified as 1 of 6 independent risk factors.^368^ In addition, a higher incidence of all‐cause and cardiovascular death has been reported in AF patients with low body weight.^372^.

Underweight is often associated with other risk factors and comorbidities such as advanced age, frailty, CKD, and cancer.^399^ As a consequence, anticoagulation is often withheld in underweight patients. When warfarin is used, it is often difficult to maintain an optimal INR range,^373^, ^400^ and even when a DOAC is used, there is concern that blood levels may become elevated, making the patient more prone to major bleeding.^401^.

In a Korean observational study comparing outcomes in underweight (≤60 kg) patients treated with a DOAC or warfarin, the DOAC (14,013 patients) was associated with a lower rate of ischemic stroke (HR 0.591, P<0.0001), major bleeding (HR 0.705, P<0.0001), intracranial bleeding (HR 0.554, P<0.0001), and all‐cause death (HR 0.705, P<0.0001), compared with warfarin (7,576 patients) after propensity score matching to adjust for confounding factors. Moreover, the superiority of the DOAC over warfarin was consistent in patients ≤50 kg.^402^.

In the J‐ELD AF registry, 1,019 (33.7%) weighed >60 kg, 1,126 (37.2%) were 50–60 kg, and 880 (29.1%) were <50 kg. Although the annual incidences of stroke/systemic embolism for each body weight group were 1.69%, 1.82%, and 1.23% (P=0.6), respectively, those of bleeding requiring hospitalization were 1.37%, 1.73%, and 2.73% (P=0.154). After adjusting for patient background in a multivariate analysis, body weight <50 kg was not a significant risk for either stroke/systemic embolism or bleeding requiring hospitalization.^403^.

The use of DOACs is preferable because of their superiority over warfarin regarding both efficacy and safety (**Table** **21**). In such cases, the CCr, which tends to be lower than the eGFR value in patients with low body weight, should be accurately ascertained. In addition, because some DOACs include body weight as a criterion for dose reduction, care should be taken to avoid inappropriate overdoses.

**TABLE 21:** Recommendations and Levels of Evidence for Treating Older Patients With AF at High Risk for Anticoagulation Therapy (Low Body Weight, Frail Patients, Patients With Dementia, and Polypharmacy).

	COR	LOE
Minimizing the number of drugs as much as possible in consideration of the need to prevent cardiovascular disease is recommended when anticoagulating patients with polypharmacy. Avoiding the use of drugs that may increase the risk of bleeding such as antiplatelet agents and NSAIDs is recommended when anticoagulating patients with polypharmacy	I	B
Anticoagulation should be considered regardless of the presence of low body weight	IIa	B
Anticoagulation should be considered regardless of frailty status	IIa	B
Anticoagulation should be considered regardless of the presence of cognitive decline (MMSE ≤23 points)	IIa	B

AF, atrial fibrillation; COR, Class of Recommendation; LOE, Level of Evidence; MMSE, Mini‐Mental State Examination; NSAIDs, nonsteroidal anti‐inflammatory drugs.

### 1.7 Frailty

Frailty is defined as a state of general weakness caused by an age‐related decline in physiological function. The prevalence of frailty increases with age, and it is reported to be 35.1% in those aged ≥85 years.^404^ Frail patients are more likely to have a variety of coexisting chronic diseases, including heart failure, dementia, COPD, diabetes, and CKD. They also have a high risk of falls, low nutritional intake, and polypharmacy. Therefore, it is generally believed that anticoagulation therapy is difficult for frail patients with such backgrounds, and as a result, anticoagulation therapy has tended to be avoided in this group of patients.

In the ANAFIE registry, 2,951 older AF patients who were assessed for frailty using the Kihon Checklist defined by the Ministry of Health, Labour and Welfare^405^ were divided into a healthy group (959 patients who scored ≤8 on the Kihon Checklist), a pre‐frail group (924 patients who scored 9–14), and a frail group (1,068 patients who scored ≥15).^406^ Anticoagulant drugs were administered to 95.6%, 94.7%, and 94.1% of patients in each respective group. The annual all‐cause mortality rates for these groups were 1.45%, 2.56%, and 7.15%, respectively (P<0.001), reflecting the patients’ backgrounds. The respective annual stroke/systemic embolism rates were 1.20%, 1.67%, and 2.37% (P=0.025), and 0.76%, 0.63%, and 1.41% per year (P=0.029) for major bleeding. In the multivariate model, the adjusted HRs for stroke/systemic embolism and major bleeding were 1.05 (P=0.857) and 1.69 (P=0.155), respectively. This relationship between frailty and patient outcomes was generally similar to that observed in the ENGAGE‐AF study.^407^.

In the ANAFIE registry, 95% of patients with AF aged ≥75 years were on anticoagulant therapy (60% with DOACs) regardless of their frailty status. Data on the effect of anticoagulation in frail patients are limited, but given that anticoagulation generally reduces ischemic stroke by about one‐third,^408^ the stroke/systemic embolism rate without anticoagulation can be estimated to be approximately 3‐fold higher than the reported rates. Although frail patients are at a higher risk of bleeding due to their multiple comorbidities, it is important to reaffirm that the benefits of anticoagulation for the prevention of embolism are greater (**Table** **21**). For frail patients, DOACs often meet the criteria for dose reduction^406^ and may offer a safety advantage over warfarin.^407^.

### 1.8 Dementia

Dementia is a syndrome rather than a disease and cognitive decline in patients with AF can be problematic for anticoagulation therapy, because of the greater risk of intracranial hemorrhage due to falls and trauma, medication errors, and low adherence.^409^ Especially in the era when warfarin was the sole oral anticoagulant, managing anticoagulation therapy in patients with cognitive impairment was challenging due to the complexity of dose adjustment and the high risk of intracranial hemorrhage. However, with the emergence of DOACs, anticoagulation in older patients, including those with cognitive impairment, has become more manageable, prompting a reconsideration of the approach to therapy.

In the ANAFIE registry, the Mini‐Mental State Examination (MMSE) was administered to 2,963 patients at enrollment to create 2 groups: a normal cognitive function group (MMSE ≥24 points) and a cognitive impairment group (MMSE ≤23 points).^410^ The cognitive impairment group was older than the normal cognitive function group (83.9 years vs. 80.7 years, P<0.001), and the prevalence of heart failure (44.7% vs. 29.4%, P<0.001) and cerebrovascular disease (35.5% vs. 23.0%, P<0.001) was higher. The all‐cause mortality rate was approximately 4‐fold higher in the cognitive impairment group than in the normal cognitive function group (9.49% vs. 2.38% per year, P<0.001). However, there were no significant differences in the rates of stroke/systemic embolism (2.11% vs. 1.65 per year, P=0.307) and major bleeding (1.30% vs. 0.75% per year, P=0.090).

Similar to the data on frail patients, these results were obtained in patient populations where more than 90% were taking anticoagulants, both in the normal cognitive function and cognitive impairment groups. Although clear data on the efficacy of anticoagulation in cognitively impaired patients are lacking, the estimated 3‐fold increase in stroke rate^408^ without anticoagulation therapy suggests that the benefit of stroke prevention outweighs the risk of major bleeding. Therefore, anticoagulation therapy should be considered for cognitively impaired older patients with AF, using DOACs in particular because of their greater ease in administration (**Table** **21**). However, it is crucial to ensure a supportive environment for the patient around medication management, including assistance from family members, facilities and on‐site medication counseling.

### 1.9 Polypharmacy

Polypharmacy is not merely defined by the high number of medications taken, but can lead to increased risks of adverse drug events, medication errors, and poor medication adherence. AF is common in older persons and is often associated with various cardiovascular and lifestyle‐related diseases that increase the number of medications taken. There are no criteria for withholding anticoagulants or for reducing the dose of anticoagulants for AF patients with polypharmacy.

Although minimizing the number of medications being taken by older AF patients is often argued, using multiple medications is not exclusively negative. Management of cardiovascular risk factors such as hypertension, diabetes mellitus, and CKD, along with preventive measures for myocardial infarction and heart failure, can improve prognosis and alleviate symptoms in many older patients.

Subanalyses of large RCTs of DOACs in AF patients^411^, ^412^ showed no increase in stroke/systemic embolism with an increasing number of drugs, but there was an increase in major bleeding and all‐cause death, which reflected the high prevalence of both multimorbidities^413^ and frail patients^414^ among those with polypharmacy. On the other hand, the absence of an increase in thrombotic events suggests the effectiveness of the administered drugs in reducing such events.

In the ANAFIE registry,^415^ the median number of medications was 6, and patients receiving ≥5^416^ accounted for approximately 60% of patients. The patients were divided into groups based on the number of medications: 0–4, 5–8, and ≥9.^417^ More than 90% of patients in each group received anticoagulants and the incidence of stroke/systemic embolism was 1.5%, 1.7%, and 1.8% per year, respectively (P=0.780), but the incidence of major bleeding increased significantly with ≥9 drugs (0.8%, 1.1%, and 1.7% per year, respectively, P<0.001). Notably, in the ELDERCARE‐AF subanalysis,^418^, ^419^ the efficacy of anticoagulation for stroke/systemic embolism prevention was larger in the high‐risk group (older, renal dysfunction), though no significant interaction was observed, suggesting at least equal or greater preventive effects in high‐risk patients. Considering that polypharmacy patients are often older with impaired renal function, it is estimated that anticoagulation therapy reduces stroke/systemic embolism by about one‐third,^408^ or even more, in polypharmacy patients. Therefore, polypharmacy patients should be treated with anticoagulation, although careful attention should be paid for increased bleeding risks under the treatment (**Table** **21**).

Questions may arise regarding the need for special dose reduction when prescribing anticoagulants to patients on a large number of medications. Warfarin interacts with various drugs,^420^ but its dose is adjusted at each clinic visit to account for these interactions. On the other hand, DOACs have fewer interactions with other drugs, with exceptions such as P‐glycoprotein inhibitors (verapamil and amiodarone), particularly for dabigatran and edoxaban. For these interactions, dose reduction recommendation/criteria are already considered and for anticoagulation of AF patients, the response to fluctuations in blood levels due to the concomitant use of other drugs is already incorporated into the dosage regimens specified in the package inserts.

In the ANAFIE registry, the increased events in the group taking ≥9 drugs included gastrointestinal bleeding (1.3%, 2.0% vs. 3.1% per year, P<0.001) and fall fracture events (4.5%, 6.6% vs. 9.2% per year, P<0.001). Special attention should be paid to falls when using warfarin, which has a high incidence of intracranial hemorrhage. The concomitant use of drugs that may increase the risk of bleeding, such as antiplatelet agents and nonsteroidal anti‐inflammatory drugs (NSAIDs), should be avoided as much as possible to prevent gastrointestinal bleeding^421^ and intracranial bleeding due to falls.^422^.

In principle, anticoagulation therapy should be administered to older AF patients with polypharmacy as described in the package insert (**Table** **21**). However, it is important to minimize the number of drugs (and doses) as much as possible, while considering the need to prevent cardiovascular disease. It is also important to avoid drugs that may increase bleeding risk.

### 1.10 Concomitant Antiplatelet Agents (Table 22)

**TABLE 22:** Recommendations and Levels of Evidence for Older Patients With AF at High Risk of Anticoagulation (With Concomitant Antiplatelet Agents).

	COR	LOE
Antiplatelet agents should not be used*	III (Harm)	B

* Combination therapy with an anticoagulant plus an antiplatelet agent should be used within 1 year after percutaneous coronary intervention (PCI), but in other cases, the use of antiplatelet agents in older patients who are eligible for anticoagulant therapy is rather harmful. In a small number of cases with very high thrombotic risk (e.g., patients with previous stent thrombosis, PCI for complex lesions, and unstable warfarin control), concomitant use of antiplatelet agents may be necessary. If there is any doubt about the decision, consultation with a specialist is highly recommended. AF, atrial fibrillation; COR, Class of Recommendation; LOE, Level of Evidence.

The combination of an anticoagulant and antiplatelet agent was a standard therapy for patients with AF and concomitant atherosclerotic disease. The AFIRE trial, a large‐scale RCT conducted in Japan, compared anticoagulant monotherapy with a combination of anticoagulant and antiplatelet agent (in this trial, the anticoagulant was rivaroxaban) in patients with AF and stable coronary artery disease, and demonstrated the superiority of anticoagulant monotherapy.^423^ Clinical guidelines in Japan as well as in Europe and the USA now recommend the use of anticoagulant monotherapy in these patients.^423^.

The AFIRE trial showed a higher risk of all‐cause death in the combination group, possibly due in part to an increase in cardiovascular events after major bleeding, especially within 30 days after major bleeding.^424^ Subgroup analyses further demonstrated the superiority of anticoagulant monotherapy also in high thrombotic risk populations such as those with previous myocardial infarction or peripheral artery disease,^425^ those with previous heart failure,^426^ those with severe coronary artery disease such as multivessel disease or left main trunk disease,^427^ and for all stent types (bare metal/drug‐eluting 1st/2nd generation).^423^, ^428^ The superiority of anticoagulant monotherapy was also more pronounced in patients with a longer time since stenting,^428^ and de‐escalation to anticoagulant monotherapy is recommended even in patients who have been on the combination therapy for many years.

Aspirin has been shown to be ineffective in preventing stroke in patients with AF,^429^ and the ESC guidelines state that antiplatelet therapy alone is not recommended for stroke prevention in patients with AF (Class III).^430^ Combination therapy with an anticoagulant plus an antiplatelet agent should be used within 1 year after percutaneous coronary intervention (PCI), but in other cases, the use of antiplatelet agents in older patients who are eligible for anticoagulant therapy is rather harmful. Therefore, this Focus Update provides a general recommendation that, in principle, antiplatelet agents should not be administered to patients with AF. However, in a small number of cases of very high thrombotic risk (e.g., patients with previous stent thrombosis, PCI for complex lesions, and unstable warfarin control), concomitant use of antiplatelet agents may be necessary. If there is any doubt about the decision, consultation with a specialist is highly recommended.

### 1.11 Edoxaban 15 mg for the Very Old at High Bleeding Risk (Table 23)

**TABLE 23:** Recommendations and Levels of Evidence for Older Patients With AF at High Risk of Anticoagulation (With Very Old Patients With High Bleeding Risk).

	COR	LOE
Edoxaban 15 mg is recommended for very elderly patients with high risk of bleeding* who are unable to receive anticoagulants at approved doses	I	B

* ≥80 years of age and having any of the following 5 conditions: (1) low CCr (15 to 30 mL/min), (2) low body weight (≤45 kg), (3) history of bleeding from a critical area or organ (including cerebral hemorrhage), (4) current use of an antiplatelet agent, and (5) continuous use of NSAIDs. However, the necessity of (4) and (5) should be examined first. AF, atrial fibrillation; CCr, creatinine clearance; COR, Class of Recommendation; LOE, Level of Evidence; NSAIDs, nonsteroidal anti‐inflammatory drugs.

All 4 DOACs are available in standard and reduced doses, which are selected according to patient background. For edoxaban, in addition to 60 mg or 30 mg once daily, a very low dose of 15 mg once daily was added in August 2021 for very old patients at high risk of bleeding based on the results of the ELDERCARE‐AF trial.^431^.

ELDERCARE‐AF included very old Japanese patients (≥80 years old) with nonvalvular AF who had a CHADS_2_ score ≥2, who were at high risk of bleeding and were considered ineligible for oral anticoagulation at approved doses. “High bleeding risk” was defined as any of the following 5 conditions: (1) low CCr (15–30 mL/min), (2) low body weight (≤45 kg), (3) history of bleeding from a critical area or organ (including cerebral hemorrhage), (4) current use of an antiplatelet agent, and (5) continuous use of NSAIDs. These patients were randomized 1 : 1 to edoxaban 15 mg or placebo to evaluate efficacy and safety. In the edoxaban 15 mg group, the primary efficacy endpoint (stroke or systemic embolism) was significantly lower (HR 0.34, P<0.001), but the primary safety endpoint (ISTH criteria major bleeding) was numerically higher but not significantly different (HR 1.87, P=0.09), indicating a benefit of the use of edoxaban 15 mg.

Following the main analysis described above, the results of the subanalyses were also presented. The age subanalysis^418^ divided patients into 3 age groups (80–84, 85–89, and ≥90 years), and the results were generally consistent across all age groups. However, there was a trend toward more major bleeding in the edoxaban group among patients aged ≥90 years, although the difference was not significant. In a subanalysis of renal function,^419^ patients were divided into 3 groups according to CCr (15–29 mL/min, 30–49 mL/min, and >50 mL/min). The results were consistent across all groups in terms of efficacy, but most major bleeding events were concentrated in the 15–29 mL/min group. A subanalysis of frailty^432^ found no difference in the efficacy or safety of edoxaban 15 mg in patients with or without frailty. Another subanalysis^433^ subdivided patients according to B‐type natriuretic peptide (BNP) level at enrollment (<200, 200–400, and >400 pg/mL), and again no difference in efficacy or safety was apparent among the 3 groups. Finally, a subanalysis^434^ of risk factors for major bleeding with edoxaban 15 mg found CCr 15–29 mL/min, anemia, and prolonged prothrombin time as significant factors.

These results indicate that edoxaban 15 mg is beneficial in very old patients with AF at high risk of bleeding who meet the ELDERCARE‐AF enrollment criteria, regardless of their clinical background. However, caution should be exercised, especially in patients aged >90 years or with CCr <30 mL/min. Insurance coverage of edoxaban 15 mg follows the ELDERCARE‐AF enrollment criteria, and there is no evidence in patients who do not meet these criteria. Patient selection should be made in accordance with the package insert. Furthermore, ELDERCARE‐AF only included patients who had not been anticoagulated within 8 weeks prior to randomization. It should be noted that there is no evidence that it is appropriate to reduce the dose of DOACs from the on‐label dose to 15 mg because the patient meets the criteria for enrollment in ELDERCARE‐AF.

A subanalysis of the Fushimi AF registry, which examined how many AF patients in daily clinical practice met the ELDERCARE‐AF criteria,^435^ revealed that 12.8% of all AF patients, and 52.9% of AF patients aged ≥80 years with a CHADS_2_ score ≥2 matched the criteria. Those matched patients were older, had more comorbidities, and a significantly higher incidence of all events compared with the non‐matched patients. Among matched patients, 48.8% were prescribed anticoagulants at enrollment, but anticoagulants were discontinued at an annual rate of 15.5% over time. Similarly, in the ANAFIE registry,^436^ matched patients (22.0% of all patients) experienced more adverse events than non‐matched patients, including stroke/systemic embolism (2‐year cumulative 3.8% vs. 2.8%), major bleeding (2.8% vs. 1.8%), all‐cause death (12.5% vs. 5.4%), and cardiovascular death (4.3% vs. 1.4%). Anticoagulants were prescribed in 89.0% of the matched patients, and there was a trend toward fewer events in the DOAC group than in the warfarin group.

## 3 SPECIFIC NEUTRALIZERS FOR FACTOR XA INHIBITORS

**FIGURE 10:**
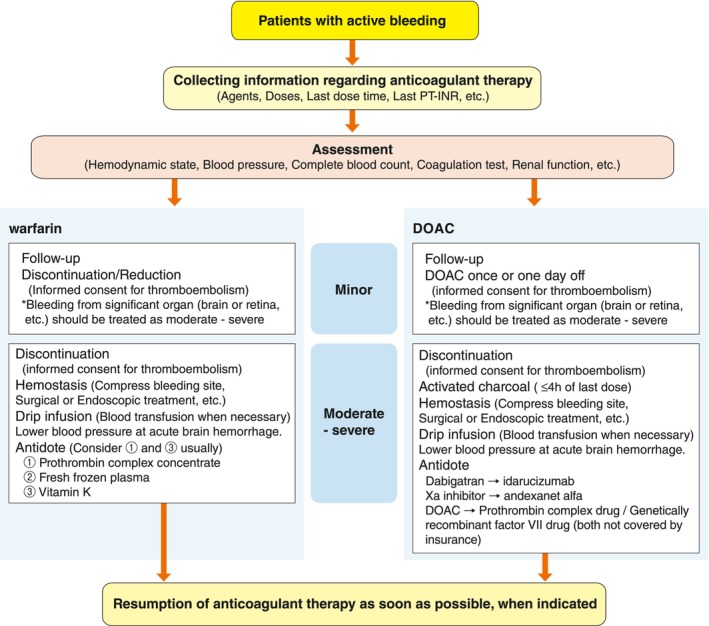
Response to active bleeding during anticoagulation therapy in patients with atrial fibrillation. DOAC, direct oral anticoagulant. (Adapted from Japanese Circulation Society. 2020.^3^)

All physicians, regardless of specialty, should be aware that andexanet alfa, a neutralizing agent for factor Xa (FXa) inhibitors (i.e., apixaban, edoxaban, and rivaroxaban), is now available (**Figure** **10**).^3^ Although the use of neutralizers for non‐major bleeding should be discouraged, all patients on oral anticoagulants should be appropriately given a neutralizing agent when life‐threatening bleeding or bleeding that is difficult to control occurs.

Andexanet alfa is a genetically engineered FXa decoy protein that has been modified to inactivate the prothrombin‐to‐thrombin catalytic activity of FXa. When andexanet alfa is administered, the FXa inhibitor binds to andexanet alfa rather than to its original target, FXa, which preserves FXa function and neutralizes the FXa inhibitory effect.

**FIGURE 11:**
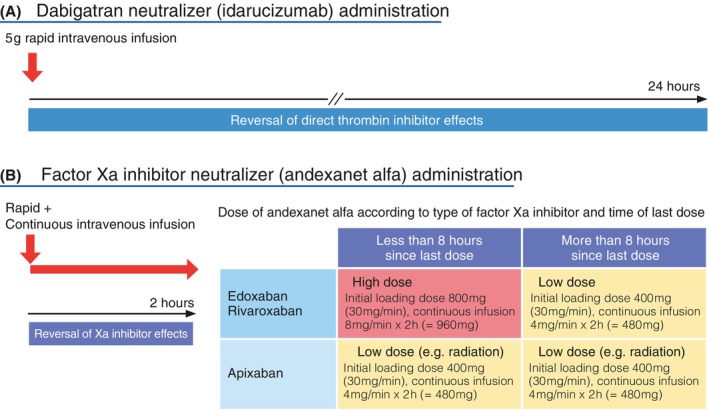
Application and effect of neutralizers for direct oral anticoagulants.

Andexanet alfa can act as a neutralizer of the 3 FXa inhibitors in a single drug when administered at high or low doses (**Figure** **11**). According to a final report^438^ of the international phase III ANNEXA‐4^437^ trial in patients with acute major bleeding within 18 h of taking an FXa inhibitor (479 patients including 19 Japanese), 93% of the apixaban group (n=172), 71% of the edoxaban group (n=28), and 94% of the rivaroxaban group (n=132) showed anti‐Xa inhibitory activity after rapid intravenous injection of andexanet alfa. The neutralizing effect was maintained until the end of 2‐h continuous intravenous infusion. Because the half‐life of andexanet alfa in blood is approximately 4 h, the neutralizing effect gradually diminished after the end of intravenous infusion, and 80% of patients achieved good hemostasis. Although 10% of patients had a post‐dose embolic event, all events occurred before the resumption of oral anticoagulant. This Focus Update recommends the use of andexanet alfa in patients with AF in the setting of life‐threatening or difficult‐to‐control bleeding that requires immediate correction of the FXa inhibitor effect (**Table** **24**).

**TABLE 24:** Recommendation and Level of Evidence for Neutralizers to FXa Inhibitors.

	COR	LOE
Administration of andexanet alfa is recommended in situations involving life‐threatening or uncontrollable bleeding, where urgent reversal of FXa inhibitor effects is required	I	B

COR, Class of Recommendation; FXa, factor Xa; LOE, Level of Evidence.

**TABLE 25:** Recommendations and Levels of Evidence for Pharmacological Treatment of Heart Rate Regulation Therapy for AF.

	COR	LOE
**Digitalis**		
If digoxin blood levels are checked regularly, oral digoxin may be considered to control heart rate or improve QOL	IIb	C

AF, atrial fibrillation; QOL, quality of life.

**Figure** **11** shows the administration method of andexanet alfa, as well as that of idarucizumab, a neutralizing agent for dabigatran that became available earlier for clinical use. In contrast to idarucizumab, which maintains its neutralizing effect for 24 h after rapid intravenous infusion, the neutralizing effect of andexanet alfa is achieved by rapid intravenous infusion followed by a 2‐h continuous infusion (**Figure** **11**). Specifically, when it is <8 h after the last dose, a higher dose is given to neutralize rivaroxaban or edoxaban, but a lower dose is given for apixaban. A lower dose is given for all FXa inhibitors if >8 h have elapsed since the last dose. Because andexanet alfa dose‐dependently inactivates the anti‐IIa and anti‐Xa activities of heparin, monitoring, such as activated clotting time (ACT), is required when using andexanet alfa under heparin administration.

Considering the circumstances in which andexanet alfa is used, any delay in administering it should be avoided. Start with a loading dose of 2 V (400 mg) at 30 mg/min, and check the appropriate dose (high or low) by the end of the loading dose administration. If the dose is high, repeat the same loading dose after completion of the initial loading dose. If the dose is low, continuous infusion is started just after completion of the initial loading dose.

A meta‐analysis of studies using idarucizumab, andexanet alfa, or a prothrombin complex concentrate at the onset of life‐threatening or difficult‐to‐control bleeding under DOAC treatment^439^ showed that 76.7% of patients in the idarucizumab group and 80.7% of patients in the andexanet alfa group achieved good hemostasis. The mortality rate was 17.4% in the idarucizumab group and 18.9% in the andexanet alfa group. The embolization rate was significantly lower in the idarucizumab group (3.8%) than in the andexanet alfa group (10.7%). In addition to neutralizers, the patient's individual risk of embolism, bleeding‐induced hypercoagulability, and withdrawal of anticoagulants can affect the incidence of embolism after major bleeding. For example, the rate of intracranial bleeding with a high risk of subsequent embolism was 69% in ANNEXA‐4^437^ with andexanet alfa, but 33% in RE‐VERSE AD,^440^ which tested the neutralizing effect of idarucizumab on dabigatran.

It is unclear whether andexanet alfa itself carries a risk of hypercoagulation and embolism in patients on FXa inhibitors who have a major bleeding event.^441^ The final results of the RCT comparing andexanet alfa to conventional therapy for intracranial bleeding in patients on Xa inhibitors (ANNEXA‐1 trial) will answer this question.

Physicians who may be involved in emergency treatment for major bleeding should confirm in advance the storage location of neutralizers for each anticoagulant and the shortest delivery route to the administration site. They should also simulate the administration method and be prepared to respond quickly and accurately when a neutralizing agent is needed. In the event of major bleeding under FXa inhibitor therapy, some institutions may not have ready access to andexanet alfa. In such cases, the use of a prothrombin complex concentrate may be considered, although it is not covered by insurance as of February 2024. In a meta‐analysis of patients with major bleeding under DOAC treatment, the prothrombin complex concentrate achieved hemostasis in 80.1%, death in 17.4%, and embolization in 4.3%, which were acceptable results compared with specific neutralizers. The study showed a 3.63‐fold increased risk of death in patients who did not achieve good hemostasis.^439^.

In Japan, where the use of DOACs is more prevalent than of warfarin, major bleeding is expected to increase in patients taking DOACs. When patients on anticoagulants develop life‐threatening bleeding or bleeding that is difficult to stop, we collect as much accurate information as possible about which anticoagulant was last taken, and use an appropriate neutralizing agent. It is important to keep in mind that anticoagulation therapy should be resumed to prevent subsequent embolisms when the patient enters a stable phase.

## 4 DIGITALIS AND ATRIAL FIBRILLATION

Digitalis has long been widely used as a heart rate regulator in AF patients. A meta‐analysis of 19 trials published between 1993 and 2014 reported that digitalis use was associated with increased mortality rates,^442^ especially in AF without heart failure (HF). Therefore, recent guidelines do not recommend the use of digitalis in patients with AF and preserved cardiac function.

Digitalis is often used clinically to control the heart rate in AF patients with reduced cardiac function, because its inotropic effects can be expected to improve cardiac function. However, previous clinical studies have reported that long‐term use of digitalis increases the mortality rate,^443^, ^444^, ^445^ and an additional analysis of the AF‐CHF trial also showed that digitalis use was related to all‐cause death, cardiac death, and arrhythmia‐related death.^443^ Based on these results, in the 2021 JCS/JHFS Guideline Focused Update on Diagnosis and Treatment of Acute and Chronic Heart Failure,^446^ long‐term use of digitalis is listed as Class III (harm). Additionally, because digitalis has an inferior effect on improving the prognosis as compared with *β*‐blockers,^445^ the JCS/JHRS 2020 Guideline on Pharmacotherapy of Cardiac Arrhythmias states that *β*‐blockers are the first choice for controlling heart rate in AF with reduced cardiac function, and digitalis is positioned as the second choice.^3^.

However, the RATE‐AF trial^447^ published in 2020 reported different outcomes.^448^ This randomized open‐label trial included 160 patients with persistent AF (mean heart rate 100±18 beats/min) with HF symptoms (NYHA class II or higher). The patients were divided into a digoxin group (mean 161 *μ*g/day) and a bisoprolol group (mean 3.2 mg/day). Doses were adjusted to achieve a heart rate of 100 beats/min or less (concomitant use of other drugs was allowed if the effect was poor), and the effects on improving quality of life (QOL) were compared. There was no significant difference in the resting heart rate (76.9±12.1 beats/min in the digoxin group vs. 74.8±11.6 beats/min in the bisoprolol group, P=0.40) at 6 months, and QOL was similar in both groups. At 12 months, the median NT‐proBNP was 960 pg/mL in the digoxin group and 1,250 pg/mL in the bisoprolol group (P=0.005), and the digoxin group exhibited better outcomes in various aspects, including NT‐proBNP level and sub‐items such as daily activity, treatment satisfaction, and NYHA class. Adverse events were also lower in the digoxin group (25% vs. 64%, P<0.001). Until now, there have been no reports showing the superiority of digitalis over *β*‐blockers in heart rate control in AF complicated by HF, but a meta‐analysis has cast doubt on the effectiveness of *β*‐blockers in improving the prognosis for AF patients complicated with HF.^449^ In view of this, they reported that the use of other drugs should be considered in a well‐balanced manner, rather than preferentially using *β*‐blockers.^448^ However, because this trial enrolled a small number of patients with only persistent AF, and evaluated the improvement of QOL and symptoms but not the long‐term prognostic efficacy, digitalis should not be simply regarded as a superior drug.

On the other hand, many of the reports that digitalis is associated with a poor prognosis have been observational studies or post‐hoc analysis of RCTs, and it has been pointed out that they may be looking at confounding between digitalis and the patients’ backgrounds.^449^ Furthermore, meta‐analyses in RCTs have shown that the digitalis has no effect on prognosis.^450^.

Considering all findings, despite the unexplored long‐term prognostic efficacy of digitalis, its recommendation level has been upgraded from Class III (harm) to Class IIb (usable) when digoxin blood levels are regularly checked (**Table** **25** ^3^).

### 4.1 Precautions in the Use of Digitalis

Digitalis, characterized by a long half‐life and renal excretion, demands therapeutic caution due to its narrow range of blood concentration. Older patients, who are often underweight and/or have poor renal function, are susceptible to increased drug effects. Therefore, the blood concentration of digitalis in those patients should be measured once or twice each year, and attending physicians should monitor for symptoms such as nausea and loss of appetite. A follow‐up analysis of an RCT involving patients with HF in sinus rhythm revealed a higher mortality rate associated with digoxin blood levels ≥1.2 ng/mL at 1 month after initiation.^448^ Maintaining the digoxin blood concentration within the range of 0.5–0.8 ng/mL was suggested to decrease the mortality rate.^448^ Therefore, for the prevention of adverse effects, measuring the blood concentration when using digoxin in AF patients is recommended. For long‐term users, regular monitoring of the digoxin blood concentration is desirable.

Digitalis is contraindicated in patients with underlying diseases such as cardiac amyloidosis or obstructive hypertrophic cardiomyopathy. The JCS 2020 Guideline on Diagnosis and Treatment of Cardiac Amyloidosis classifies digitalis usage as Class III,^451^ with the rationale being that digitalis binds to amyloid proteins, increasing the drug sensitivity and potentially leading to fatal arrhythmias. Additionally, because digitalis is a substrate of P‐glycoproteins, the concomitant use with drugs such as amiodarone and verapamil, and diuretics (spironolactone, tolvaptan) should be carefully monitored due to an increased blood concentration of those drugs.

## 5 ATRIAL FIBRILLATION AND LIFESTYLE MANAGEMENT / COMPREHENSIVE MANAGEMENT

### 5.1 Atrial Fibrillation and Lifestyle Management

AF is common not only among older adults but also among middle‐aged adults with lifestyle‐related diseases such as hypertension, and it causes complications such as thromboembolism, stroke, and HF.^430^ Therefore, it is important to manage and guide patients with AF not only to treat AF itself but also to reduce their risk of developing cardiovascular complications and diseases. The JCS/JHRS 2020 Guideline on Pharmacotherapy of Cardiac Arrhythmias^3^ notes the importance of management of comorbidities and lifestyle (HF, valvular heart disease, hypertension, diabetes mellitus, obstructive sleep disorder, CKD, obesity, and smoking) in patients with AF. This Focus Update adds recommendations regarding alcohol and caffeine, for which new evidence has been reported, and physical activity as lifestyle management strategies.

#### 5.1.1 Alcohol and Caffeine

Excessive alcohol consumption is a known risk factor for developing AF,^452^, ^453^, ^454^, ^455^ and is also a risk factor for bleeding during anticoagulation therapy.^456^ The JCS/JHRS 2020 Guideline on Pharmacotherapy of Cardiac Arrhythmias^3^ recommends that the risk of bleeding complications in patients with AF should be assessed using the HAS‐BLED score (Recommendation Class I), and heavy alcohol drinking is one of the components of the HAS‐BLED score. In addition, excessive alcohol consumption by patients with AF increases the risk of thromboembolism and death.^457^ A recent RCT reported that abstinence from alcohol reduces recurrent AF in patients with AF who are regular drinkers.^458^.

In this Focus Update, therefore, patients with AF who are being considered for prophylaxis and anticoagulation should be advised and managed to avoid excessive alcohol consumption (**Table** **26**, Recommendation Class IIa).

**TABLE 26:** Recommendation and Level of Evidence for Alcohol Consumption by Patients With AF.

	COR	LOE
Advice and management to avoid excessive alcohol consumption should be considered	IIa	B

AF, atrial fibrillation; COR, Class of Recommendation; LOE, Level of Evidence.

Caffeine intake is considered a risk factor for the development of supraventricular extrasystoles, which trigger the onset of AF.^459^ The JCS/JHRS 2020 Guideline on Pharmacotherapy of Cardiac Arrhythmias^3^ recommends limiting caffeine intake when supraventricular extrasystoles compromise QOL (Recommendation Class I). However, recent reports indicate that adequate caffeine intake does not increase the risk of AF,^460^, ^461^ and that habitual coffee consumption of 1–3 cups/day reduces the risk of developing AF.^462^ On the other hand, it should be noted that caffeine intake may increase palpitation symptoms unrelated to AF.^463^.

#### 5.1.2 Physical Activity

Many clinical studies have shown that moderate exercise and physical activity are beneficial to cardiovascular health.^464^ However, a higher incidence of AF can be seen in athletes, and several small clinical studies have reported that intense physical activity (mainly endurance sports) increases the incidence of AF.^465^, ^466^, ^467^ On the other hand, in a small number of studies (25 controls vs. 26 receiving exercise therapy), the cumulative duration of AF (AF burden) increased over time without exercise therapy in AF patients, whereas appropriate exercise therapy significantly suppressed the AF burden.^468^ In light of these findings, active exercise should be encouraged to prevent the development and recurrence of AF. However, excessive endurance exercise (e.g., marathons and long‐distance triathlons) with high cardiovascular load should be avoided, especially for those aged >50 years.

The benefits of cardiac rehabilitation for patients with chronic HF have been attracting attention. Interestingly, cardiac rehabilitation has been reported to improve exercise tolerance and QOL in patients with AF complicated by HF.^469^ The JCS/JACR 2021 Guideline on Rehabilitation in Patients With Cardiovascular Disease^470^ recommends that exercise therapy be considered to improve exercise tolerance and QOL in AF patients with reduced exercise tolerance or those with concomitant HF (Recommendation Class IIa). Note, however, that exercise therapy is relatively contraindicated in patients with AF tachycardia whose heart rate is not under control.^470^.

The degree to which exercise and physical activity are effective in patients with AF has not been fully elucidated. In general, it is recommended to maintain an appropriate intensity of exercise while monitoring the patient's heart rate, blood pressure response, and symptom onset. This Focus Update recommends that patients with AF should be instructed to engage in moderate physical activity to prevent the onset or recurrence of AF (**Table** **27**, Recommendation Class IIa).

**TABLE 27:** Recommendation and Level of Evidence for Physical Activity in Patients With AF.

	COR	LOE
Educating moderate physical activity to prevent onset or recurrence of AF (excluding excessive endurance exercise, which may induce AF) should be considered	IIa	C

AF, atrial fibrillation; COR, Class of Recommendation; LOE, Level of Evidence.

### 5.2 Comprehensive Management of Patients With Atrial Fibrillation

In 2050, the number of patients with AF in Japan is projected to be approximately 1.03 million, accounting for almost 1.1% of the total population.^3^ The increasing prevalence of AF is mainly due to the aging of society and the increase in risk factors and comorbidities. Complications caused by AF, such as cerebral infarction and HF, contribute to the strain on medical resources and rising medical costs.^430^, ^471^, ^472^.

To correct these problems, the following approaches are necessary: (1) early diagnosis of AF, (2) understanding the characteristics of individual patients with AF, and (3) comprehensive management. First, (1) the diagnosis of AF is based on ECG recordings, either 12‐lead ECG recordings or unipolar ECG recordings of ≥30 s. Next, (2) the risk of cerebral infarction, degree of symptoms, duration of AF including whether it is paroxysmal or persistent, cardiac status and cardiovascular risk factors that may cause the onset and progression of AF are evaluated in each patient. (3) The patient's comorbidities and lifestyle should be taken into account in the integrated therapeutic intervention. The JCS/JHRS 2020 Guideline on Pharmacotherapy of Cardiac Arrhythmias proposed 5 treatment steps for the acute and chronic management of patients with AF (Step 1: Acute rate and rhythm control, Step 2: Manage precipitating factors, Step 3: Assess stroke risk, Step 4: Assess heart rate, and Step 5: Assess symptoms).^3^.

Recently, the ESC proposed the ABC pathway (“A” Anticoagulation/Avoid stroke: anticoagulation and stroke prevention, “B” Better symptom management: symptom improvement, “C” Cardiovascular and Comorbidity optimization: detection and management of aggravating factors), which aims to provide integrated treatment for patients with AF.^430^ The implementation of the ABC pathway has been reported to improve all‐cause mortality rates and the composite of stroke/major bleeding/cardiovascular death, and first hospitalization,^473^ reduce the incidence of cardiovascular events,^474^, ^475^ and lower healthcare‐related costs.^476^.

Regarding rhythm vs. rate control, the AFFIRM study^477^ compared a rhythm control group that actively maintained sinus rhythm with antiarrhythmic drugs with a rate control group that underwent heart rate control to reduce symptoms about 20 years ago, and found no significant difference in all‐cause deaths (HR 1.15, 95% confidence interval [CI] 0.99–1.34, P=0.08). Rate control was recognized as a safe treatment option that was comparable to rhythm control. However, catheter ablation for AF was not widely used at that time, and this finding needs to be reevaluated now that catheter ablation is widely used. To address this issue, the EAST‐AFNET 4 trial^267^ (1,395 patients in the early rhythm control group vs. 1,394 patients in the rate control [usual care] group) compared the efficacy and safety of early rhythm control (with antiarrhythmic drugs or catheter ablation) with rate control in patients with early onset AF (<1 year after initial diagnosis), and revealed that the early rhythm control significantly reduced the incidence of cardiovascular event (composite of death from cardiovascular cause, stroke, or hospitalization with worsening of HF or acute coronary syndrome) (3.9/100 patient‐years vs. 5.0/100 patient‐years, HR 0.79, 96% CI 0.66–0.94, P=0.005). In addition, a Korean cohort study enrolling 22,635 patients with AF was followed for 2 years for composite endpoints of cardiovascular death, stroke, heart failure hospitalization, and acute myocardial infarction. Patients with early onset AF within 1 year had fewer composite endpoints in the rhythm control group than in the rate control group (HR 0.81, 95% CI 0.71–0.93, P=0.002), but there was no difference between the two groups for patients with AF more than 1 year after onset (HR 0.97, 95% CI 0.78–1.20, P=0.76).^478^ Such reports have recently pointed out the importance of early rhythm control before the development of adverse events such as atrial remodeling.^479^ However, it should be noted that in this trial,^267^ there were many first‐episode AF patients, and many patients in both groups were not receiving rhythm control therapy at 2 years (34.9% in the early rhythm control therapy group vs. 85.4% in the rate control [usual care] group).

In response to this recent reaffirmation of the importance of rhythm control, this Focus Update makes a minor revision to the 5‐step treatment for patients with AF in the JCS/JHRS 2020 Guideline on Pharmacotherapy of Cardiac Arrhythmias, and describes rhythm control and rate control in parallel as “Step 4: Improvement of symptoms”. The choice of treatment between rhythm control and rate control is determined on a case‐by‐case basis.

#### 5.2.1 4‐Step Management of Patients With Atrial Fibrillation (Step 1: Acute Management) (Figure 12)

**FIGURE 12:**
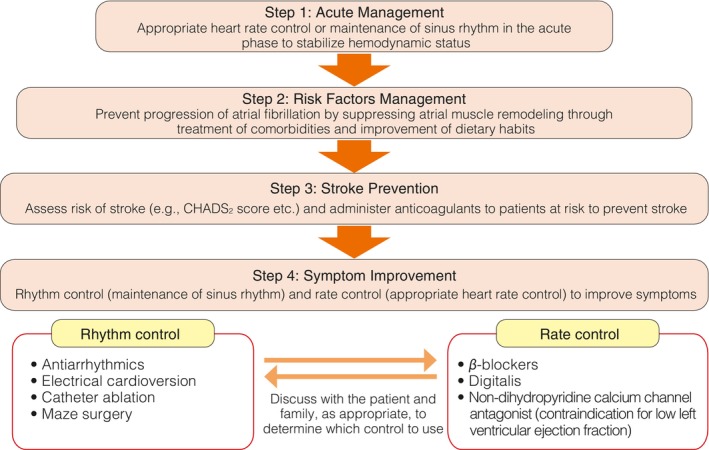
Comprehensive management of atrial fibrillation.

Appropriate heart rate control or maintenance of sinus rhythm should be performed during the acute phase to stabilize hemodynamics. If the patient is hemodynamically compromised, emergency electrical cardioversion should be performed to restore sinus rhythm.

#### 5.2.2 4‐Step Management of Patients With Atrial Fibrillation (Step 2: Risk Factors Management) (Figure 12)

Appropriate treatment of comorbidities and improved dietary habits reduce the risk of cardiovascular events associated with the development and progression of AF. Comorbidities (hypertension, HF, coronary artery disease, diabetes, sleep apnea, etc.) and unfavorable lifestyle habits (obesity, smoking, excessive alcohol intake, lack of physical activity, etc.) play a major role in the development of complications in patients with AF. Step 2 includes identification and appropriate management of these comorbidities and lifestyle habits.

For example, improving modifiable risk factors such as reducing weight, smoking cessation, reduction of excessive alcohol intake, and regular physical activity can reduce atrial remodeling as well as recurrent AF. Targeted interventions (119 patients) for risk factors such as hypertension, dyslipidemia, HF, diet, and cardiac rehabilitation as compared with the control group (126 patients) resulted in significantly higher maintenance of sinus rhythm (75% vs. 63%, odds ratio 1.765, 95% CI lower limit 1.021, P<0.042).^480^ In contrast, a study of aggressive blood pressure lowering alone in patients with AF after catheter ablation showed no effect on AF recurrence (HR 0.94, 95% CI 0.65–1.38, P=0.763),^481^ which suggests that comprehensive improvement, rather than correction of single risk factors, is needed to prevent recurrence and improve prognosis in AF.

In this Focus Update, we recommend the importance of treating comorbidities such as hypertension, diabetes, and sleep apnea, as well as improving lifestyle habits such as obesity, smoking, and excessive alcohol consumption, to reduce the risk of developing AF and recurrent AF (**Table** **28**, Recommendation Class I).

**TABLE 28:** Recommendations and Levels of Evidence for Rhythm Control in Patients With AF.

	COR	LOE
Treatment of comorbidities such as hypertension, diabetes, and sleep apnea, and modifications of lifestyle such as obesity, smoking, and excessive alcohol consumption to enhance the effectiveness of rhythm control are recommended	I	B
Rhythm control therapy in patients with early‐stage AF should be considered	IIa	A

AF, atrial fibrillation; COR, Class of Recommendation; LOE, Level of Evidence.

#### 5.2.3 4‐Step Management of Patients With Atrial Fibrillation (Step 3: Stroke Prevention) (Figure 12)

The risk of stroke is assessed, and patients at risk are given oral anticoagulants. AF increases the risk of stroke by approximately 5‐fold,^482^ but this risk is not uniform across patients with AF and is increased by the presence of stroke risk factors and their modifiers.^483^ Previously, it was recognized that the risk of thromboembolism was similar whether the AF was paroxysmal or persistent. However, a recent meta‐analysis found that persistent AF was associated with a higher risk of thromboembolism (HR 1.38, 95% CI 1.19–1.61, P<0.001) compared with paroxysmal AF.^484^ In the J‐RISK study^368^ conducted in Japan, persistent/permanent AF was also considered an independent risk factor contributing to the development of stroke (for details, see **Chapter III.1.1**). Assessment of stroke risk in patients with AF must strike a balance between simplicity, practicality, and accuracy.^485^, ^486^.

In the JCS/JHRS 2020 Guideline on Pharmacotherapy of Cardiac Arrhythmias,^3^ risk assessment for stroke was based on the CHADS_2_ score (HF, hypertension, age >75 years, diabetes, history of stroke or transient ischemic attack),^487^ but this Focus Update recommends the use of the CHADS_2_ score and HELT‐E_2_S_2_ score (**Table** **19**).

Oral anticoagulants reduce the risk of stroke and death in patients with AF.^488^ They include vitamin K antagonists (warfarin) and DOACs, but DOACs are preferred because of their ease of administration, stable efficacy, fewer interactions with diet and other drugs, and less intracranial bleeding.^430^, ^489^, ^490^ Warfarin should be used only when a DOAC is not available. On the other hand, the question of whether DOACs should be administered to patients at very high risk of bleeding has long been debated. To address this issue, the superiority of edoxaban 15 mg once daily over placebo in preventing stroke or systemic embolism in Japanese patients aged >80 years with nonvalvular AF who were unable to receive the approved doses of existing oral anticoagulants due to bleeding concerns was verified^431^ (see **Chapter III.2.7** for details). In patients who require long‐term anticoagulation but for whom anticoagulation is not appropriate due to high bleeding risk, percutaneous left atrial appendage closure or thoracoscopic left atrial appendage closure may be considered on a case‐by‐case basis.^491^.

#### 5.2.4 4‐Step Management of Patients With Atrial Fibrillation (Step 4: Symptom Improvement) (Figure 12)

Symptom improvement is achieved by maintaining sinus rhythm (Rhythm control) and/or appropriate heart rate control (Rate control). Rhythm control is a therapeutic strategy to return to and maintain sinus rhythm. Antiarrhythmic drugs, electrical cardioversion, and catheter ablation/Maze procedure are the primary means of rhythm control. Return to and maintenance of sinus rhythm are effective in improving symptoms of AF, exercise capacity,^282^ QOL,^492^ left ventricular ejection fraction (LVEF),^280^ and left atrial diameter,^283^ and reducing hospitalizations.^493^.

In recent years, the ablation technology for AF has evolved dramatically, and its efficacy and safety have improved markedly. In the CASTLE‐AF trial,^279^ catheter ablation in AF patients complicated by HF with reduced LVEF (HFrEF) significantly reduced rates of all‐cause mortality (HR 0.53, 95% CI 0.32–0.86, P=0.01) and hospitalization for worsening HF (HR 0.62, 95% CI 0.43–0.87, P=0.007) compared with medical treatment (Rate control). On the other hand, in the aforementioned EAST‐AFNET 4 trial,^267^ the rate of catheter ablation at 2 years after allocation was not so high (19.4%), and the proportion of patients receiving antiarrhythmic drugs was 45.7%, indicating the usefulness of antiarrhythmic drugs for early‐stage AF.

**Table** **29** shows patient profile for atrial fibrillation in which rhythm control is considered preferable. Rhythm control with catheter ablation and/or antiarrhythmic drugs should be aggressively considered for these patients. With regard to age, we note that in a Korean cohort study^494^ the benefit of early rhythm control waned with increasing age and was more beneficial in patients with AF who were younger than 75 years. Given the results of the EAST‐AFNET 4 trial, rhythm control should be a priority, at least in patients with early‐stage AF, and this Focus Update recommends that rhythm control therapy be considered for patients with early‐stage AF to improve symptoms and QOL (**Table** **28**, Recommendation Class IIa).

**TABLE 29:** Patient Profile for AF in Which Rhythm Control Is Considered Preferable.

(1) Severe symptoms (palpitations, syncope, chest discomfort, etc.)
(2) Persistence of AF which can lead to the onset or worsening of heart failure
(3) Relatively few comorbidities related to the development of AF
(4) No significant severe left atrial enlargement and minimal intra‐atrial conduction delay (mild atrial remodeling)
(5) Patient preference for rhythm control

AF, atrial fibrillation.

On the other hand, rate control is intended to prevent the onset or worsening of palpitations and HF by appropriately controlling heart rate during AF. Patients with persistent or permanent AF who are considered to have difficulty maintaining sinus rhythm are the main target. Drugs used include *β*‐blockers, digitalis, and nondihydropyridine calcium antagonists (contraindication for patients with low cardiac function).

## APPENDIX 1

### Details of Members

**Chairs**

- Yu‐ki Iwasaki, Department of Cardiovascular Medicine, Nippon Medical School
- Takashi Noda, Department of Cardiology, Tohoku University Hospital (Deputy Chief)

.

**Members**

- Masaharu Akao, Department of Cardiology, National Hospital Organization Kyoto Medical Center
- Tadashi Fujino, Department of Cardiovascular Medicine, Toho University Faculty of Medicine
- Teruyuki Hirano, Department of Stroke Medicine, Kyorin University School of Medicine
- Koichi Inoue, Department of Cardiology, National Hospital Organization Osaka National Hospital
- Takashi Kurita, Division of Cardiovascular Center, Kindai University School of Medicine
- Kengo Kusano, Department of Cardiovascular Medicine, National Cerebral and Cardiovascular Center
- Toshiyuki Nagai, Department of Cardiovascular Medicine, Faculty of Medicine and Graduate School of Medicine, Hokkaido University
- Kazuhiro Satomi, Department of Cardiology, Tokyo Medical University Hospital
- Wataru Shimizu, Department of Cardiovascular Medicine, Nippon Medical School
- Tetsuji Shinohara, Department of Cardiology and Clinical Examination, Faculty of Medicine, Oita University
- Kyoko Soejima, Department of Cardiovascular Medicine, Kyorin University School of Medicine
- Yohei Sotomi, Department of Cardiovascular Medicine, Osaka University Graduate School of Medicine
- Shinya Suzuki, Department of Cardiovascular Medicine, The Cardiovascular Institute
- Hiroshi Tada, Department of Cardiovascular Medicine, Faculty of Medical Sciences, University of Fukui
- Teiichi Yamane, Department of Cardiology, The Jikei University School of Medicine

.

**Collaborators**

- Tsukasa Kamakura, Department of Cardiovascular Medicine, National Cerebral and Cardiovascular Center
- Hiroyuki Kato, Department of Cardiology, Japan Community Healthcare Organization Chukyo Hospital
- Arimi Katsume, Department of Cardiovascular Medicine, Kyorin University School of Medicine
- Yusuke Kondo, Department of Cardiovascular Medicine, Chiba University Graduate School of Medicine
- Kenji Kuroki, Department of Cardiology, Faculty of Medicine, University of Yamanashi
- Hisaki Makimoto, Division of Cardiovascular Medicine, Department of Internal Medicine, Data Science Center, Jichi Medical University
- Hiroshige Murata, Department of Cardiology, Nippon Medical School Hospital
- Takafumi Oka, Department of Cardiovascular Medicine, Osaka University Graduate School of Medicine
- Nobuaki Tanaka, Department of Cardiology, Cardiovascular Center, Sakurabashi Watanabe Hospital
- Nobuhiko Ueda, Department of Cardiovascular Medicine, National Cerebral and Cardiovascular Center
- Hiro Yamasaki, Department of Cardiology, Institute of Medicine, University of Tsukuba
- Seigo Yamashita, Department of Cardiology, The Jikei University School of Medicine
- Ryobun Yasuoka, Department of Cardiology, Kindai University School of Medicine
- Kenji Yodogawa, Department of Cardiology, Nippon Medical School Hospital

.

**Independent Assessment Committee**

- Kazutaka Aonuma, Department of Cardiology, Mito Saiseikai General Hospital
- Takanori Ikeda, Department of Cardiology, Toho University Medical Center Omori Hospital
- Toru Minamino, Department of Cardiovascular Medicine, Juntendo University Graduate School of Medicine
- Hideo Mitamura, National Public Service Mutual Aid Federation Tachikawa Hospital
- Akihiko Nogami, Tokyo Heart Summit Tokyo Cardiac Arrhythmia Hospital
- Ken Okumura, Department of Cardiology, Cardiovascular Center, Saiseikai Kumamoto Hospital

.

(Listed in alphabetical order; affiliations as of March 2024).

## APPENDIX 2

### DISCLOSURE OF POTENTIAL CONFLICTS OF INTEREST (COI): JCS/JHRS 2024 GUIDELINE FOCUSED UPDATE ON MANAGEMENT OF CARDIAC ARRHYTHMIAS (2021/1/1–2023/12/31)

**Table:**

Author	Member's own declaration items									COI of the marital partner, first‐degree family members, or those who share income and property			COI of the head of the organization/department to which the member belongs (if the member is in a position to collaborate with the head of the organization/department)	
	Employer/leadership position (private company)	Stakeholder	Patent royalty	Honorarium	Payment for manuscripts	Research grant	Scholarship (educational) grant	Endowed chair	Other rewards	Employer/leadership position (private company)	Stakeholder	Patent royalty	Research grant	Scholarship (educational) grant
Chair: Takashi Noda (Deputy Chief)				Medtronic Japan Co., Ltd. BIOTRONIK Japan, Inc. Bayer Yakuhin, Ltd.		Johnson & Johnson K.K.								
Members: Masaharu Akao*				Daiichi Sankyo Company, Limited. Bayer Yakuhin, Ltd. Bristol‐Myers Squibb.			Daiichi Sankyo Company, Limited. Bayer Yakuhin, Ltd.							
Members: Teruyuki Hirano*				Daiichi Sankyo Company, Limited. Bayer Yakuhin, Ltd. Pfizer Japan Inc. Nippon Boehringer Ingelheim Co., Ltd. Otsuka Pharmaceutical Co., Ltd.										
Members: Koichi Inoue				Johnson & Johnson K.K. Medtronic Japan Co., Ltd. Japan Lifeline Co.,Ltd. Nippon Boehringer Ingelheim Co., Ltd. Bayer Yakuhin, Ltd. Pfizer Japan Inc. Boston Scientific Japan K.K. Daiichi Sankyo Company, Limited. Bristol‐Myers Squibb.										
Members: Takashi Kurita*				Medtronic Japan Co., Ltd. Bayer Yakuhin, Ltd. BIOTRONIK Japan, Inc. Daiichi Sankyo Company, Limited. TOA EIYO LTD. Abbott Medical Japan LLC. Japan Lifeline Co.,Ltd.										
Members: Kengo Kusano*				Medtronic Japan Co., Ltd. Daiichi Sankyo Company, Limited. Bayer Yakuhin, Ltd. Nippon Boehringer Ingelheim Co., Ltd. Pfizer Japan Inc.		Hitachi, Ltd. Daiichi Sankyo Company, Limited. Medtronic Japan Co., Ltd. BIOTRONIK Japan, Inc. Mebix, Inc. JSR Corporation. IQVIA Services Japan K.K. Boston Scientific Japan K.K. Abbott Medical Japan LLC. GE Precision Healthcare. EPS Corporation.								
Members: Toshiyuki Nagai				Kyowa Kirin Co., Ltd. Bayer Yakuhin, Ltd. Viatris Inc. Nippon Boehringer Ingelheim Co., Ltd.			Mitsubishi Tanabe Pharma Corporation							
Members: Kazuhiro Satomi*				Medtronic Japan Co., Ltd. Abbott Medical Japan LLC. Japan Lifeline Co.,Ltd. TERUMO CORPORATION. Bayer Yakuhin, Ltd. Boston Scientific Japan K.K.		Johnson & Johnson K.K.	Abbott Medical Japan LLC. Nippon Boehringer Ingelheim Co., Ltd.							
Members: Wataru Shimizu				Daiichi Sankyo Company, Limited. Nippon Boehringer Ingelheim Co., Ltd. Bayer Yakuhin, Ltd. Bristol‐Myers Squibb. Pfizer Japan Inc. Ono Pharmaceutical Co., Ltd. Novartis Pharma K.K. Otsuka Pharmaceutical Co., Ltd. Medtronic Japan Co., Ltd. Abbott Medical Japan LLC. Johnson & Johnson K.K.										
Members: Kyoko Soejima*				Daiichi Sankyo Company, Limited. Abbott Medical Japan LLC. Medtronic Japan Co., Ltd. Johnson & Johnson K.K. Nippon Boehringer Ingelheim Co., Ltd. BIOTRONIK Japan, Inc. Boston Scientific Japan K.K.										
Members: Yohei Sotomi*				Bristol‐Myers Squibb. Abbott Medical Japan LLC. Boston Scientific Japan K.K.		Abbott Medical Japan LLC. TOA EIYO LTD. Johnson & Johnson K.K. Roche Diagnostics K.K. Bristol‐Myers Squibb. Nipro Corporation.		TOA EIYO LTD. TERUMO CORPORATION. Nipro Corporation. SHIMADZU CORPORATION. ASAHI INTECC CO., LTD.						
Members: Shinya Suzuki				Bristol‐Myers Squibb. Daiichi Sankyo Company, Limited.										
Members: Hiroshi Tada				Medtronic Japan Co., Ltd. BIOTRONIK Japan, Inc. Boston Scientific Japan K.K. Daiichi Sankyo Company, Limited. Bristol‐Myers Squibb. Novartis Pharma K.K.			Abbott Medical Japan LLC. DAIICHI SANKYO COMPANY, Ltd. Nippon Boehringer Ingelheim Co., Ltd. Otsuka Pharmaceutical Co., Ltd. Eli Lilly Japan K.K. Marubun Tsusyo K.K.							
Members: Teiichi Yamane				Medtronic Japan Co., Ltd. Abbott Medical Japan LLC. BEG Co.,Ltd.			Japan Lifeline Co.,Ltd.							
Collaborators: Tsukasa Kamakura				Johnson & Johnson K.K. Medtronic Japan Co., Ltd. BIOTRONIK Japan, Inc.										
Collaborators: Hiroyuki Kato				Medtronic Japan Co., Ltd. Abbott Medical Japan LLC.										
Collaborators: Yusuke Kondo				Boston Scientific Japan K.K. Abbott Medical Japan LLC. BIOTRONIK Japan, Inc. Daiichi Sankyo Company, Limited. Bayer Yakuhin, Ltd.										
Collaborators: Kenji Kuroki						Medtronic Japan Co., Ltd. Abbott Medical Japan LLC.								
Collaborators: Hisaki Makimoto						Mitsubishi Electric Corporation								
Collaborators: Hiroshige Murata				Pfizer Japan Inc.										
Collaborators: Nobuaki Tanaka				Bayer Yakuhin, Ltd. Johnson & Johnson K.K. Nippon Boehringer Ingelheim Co., Ltd. AstraZeneca K.K. Medtronic Japan Co., Ltd.										
Collaborators: Nobuhiko Ueda				Medtronic Japan Co., Ltd.										
Collaborators: Hiro Yamasaki				Medtronic Japan Co., Ltd. Abbott Medical Japan LLC. Boston Scientific Japan K.K.	TORAY INDUSTRIES, INC.									
Independent Assessment Committee: Takanori Ikeda				Bayer Yakuhin, Ltd. Daiichi Sankyo Company, Limited. Pfizer Japan Inc.										
Independent Assessment Committee: Toru Minamino	Fukuda Denshi Co., Ltd.			Bayer Yakuhin, Ltd. Daiichi Sankyo Company, Limited. Nippon Boehringer Ingelheim Co., Ltd. AstraZeneca K.K. Novo Nordisk Pharma Ltd. Novartis Pharma K.K. Sumitomo Pharma Co., Ltd. Kowa Company, Ltd.			Medical Hearts co.,Ltd. Crosswill Medical co.,Ltd. Eisai Co., Ltd. BIOTRONIK Japan, Inc. Daiichi Sankyo Company, Limited. Boston Scientific Japan K.K. Otsuka Pharmaceutical Co., Ltd. PPD‐SNBL K.K. MC, Inc. Nippon Boehringer Ingelheim Co., Ltd. Active Medical Co.,Ltd. Medtronic Japan Co., Ltd. Abbott Medical Japan LLC. Japan Lifeline Co.,Ltd. ALVAUS Inc. Shionogi & Co., Ltd. Mitsubishi Tanabe Pharma Corporation. Roche Diagnostics K.K. Ono Medical Research Foundation.							
Independent Assessment Committee: Akihiko Nogami				Johnson & Johnson K.K. Abbott Medical Japan LLC. Nippon Boehringer Ingelheim Co., Ltd. Medtronic Japan Co., Ltd.				Medtronic Japan Co., Ltd. DVx Inc.						
Independent Assessment Committee: Ken Okumura	Japan Lifeline Co.,Ltd. Medtronic Japan Co., Ltd.			Daiichi Sankyo Company, Limited. Bristol‐Myers Squibb. Nippon Boehringer Ingelheim Co., Ltd. Johnson & Johnson K.K. Bayer Yakuhin, Ltd.										

Notation of corporation is omitted.

The following persons have no conflict of interest to declare:

Chair: Yu‐ki Iwasaki.

Members: Tadashi Fujino.

Members: Tetsuji Shinohara.

Collaborators: Arimi Katsume.

Collaborators: Takafumi Oka.

Collaborators: Seigo Yamashita.

Collaborators: Ryobun Yasuoka.

Collaborators: Kenji Yodogawa.

Independent Assessment Committee: Kazutaka Aonuma.

Independent Assessment Committee: Hideo Mitamura.

In accordance with “The JAMS COI Management Guidance on Eligibility Criteria for Clinical Practice Guideline Formulation 2023”, all members have submitted their COIs for the past 3 years.

Some members (*) reported under the category of “Amount Category 3” or “Endowed departments established through donations by a company” and therefore do not have a vote in the guideline formulation process, to ensure fairness and transparency of the guidelines.

All costs associated with the development of this guideline were borne by the Japanese Circulation Society, and no funding was received from any private companies or for‐profit organizations

.
